# Systematic Review of Antimicrobial Combination Options for Pandrug-Resistant *Acinetobacter baumannii*

**DOI:** 10.3390/antibiotics10111344

**Published:** 2021-11-03

**Authors:** Stamatis Karakonstantis, Petros Ioannou, George Samonis, Diamantis P. Kofteridis

**Affiliations:** Department of Internal Medicine & Infectious Diseases, University Hospital of Heraklion, 71110 Heraklion, Crete, Greece; petros_io@hotmail.com (P.I.); samonis@med.uoc.gr (G.S.); kofterid@med.uoc.gr (D.P.K.)

**Keywords:** *Acinetobacter*, pandrug-resistant, antimicrobial combinations, synergy

## Abstract

Antimicrobial combinations are at the moment the only potential treatment option for pandrug-resistant *A. baumannii*. A systematic review was conducted in PubMed and Scopus for studies reporting the activity of antimicrobial combinations against *A. baumannii* resistant to all components of the combination. The clinical relevance of synergistic combinations was assessed based on concentrations achieving synergy and PK/PD models. Eighty-four studies were retrieved including 818 eligible isolates. A variety of combinations (*n* = 141 double, *n* = 9 triple) were tested, with a variety of methods. Polymyxin-based combinations were the most studied, either as double or triple combinations with cell-wall acting agents (including sulbactam, carbapenems, glycopeptides), rifamycins and fosfomycin. Non-polymyxin combinations were predominantly based on rifampicin, fosfomycin, sulbactam and avibactam. Several combinations were synergistic at clinically relevant concentrations, while triple combinations appeared more active than the double ones. However, no combination was consistently synergistic against all strains tested. Notably, several studies reported synergy but at concentrations unlikely to be clinically relevant, or the concentration that synergy was observed was unclear. Selecting the most appropriate combinations is likely strain-specific and should be guided by in vitro synergy evaluation. Furthermore, there is an urgent need for clinical studies on the efficacy and safety of such combinations.

## 1. Introduction

Pandrug-resistant (PDR) Gram-negative bacteria, resistant to all currently available antibiotics, including carbapenems, aminoglycosides, polymyxins and tigecycline, have been increasingly reported worldwide [1]. Especially problematic is the management of infections by PDR *A. baumannii* (PDRAB), since there are no monotherapy treatment options and associated mortality is very high [2]. Cefiderocol, where available, is a last resort option [3]. However, resistance to cefiderocol is already being reported and is likely to increase, considering the high prevalence of heteroresistance to this agent [4], as has occurred with polymyxins [5]. Therefore, pending approval of new antimicrobials, synergistic combinations are at the moment the only potential treatment option for PDRAB [6].

Combination antimicrobial therapy compared to monotherapy has not so far been proven in most studies to lead to better clinical outcomes of *A. baumannii* infections [7,8,9,10,11]. However, the available studies are predominantly based on combinations including at least one active antimicrobial and a potential benefit in PDRAB infections, with no monotherapy treatment options, should not be excluded [6,12,13]. Similar to clinical studies, prior systematic reviews that have assessed the in vitro synergy of various combinations (based on polymyxins [14,15,16], rifampin [14,16], meropenem [16,17] or tigecycline [16,18]) against *A. baumannii*, were predominantly based on studies testing combinations including at least one active antimicrobial. However, synergy testing may be most useful to identify combinations for salvage therapy of infections by bacteria resistant to all monotherapy treatment options [19].

Therefore, the purpose of this systematic review is to identify synergistic combinations that may be used for treatment of infections caused by PDRAB, i.e., combinations based on antimicrobials to which *A. baumannii* is resistant. Furthermore, it was evaluated whether the identified combinations were synergistic at concentrations achievable in vivo, a major consideration when assessing the in vivo relevance of in vitro synergy [20], especially when referring to PDRAB. These data aim to aid microbiology laboratories and infectious disease clinicians to prioritize the potential combination options for evaluation for synergy against the local PDRAB strains.

## 2. Methods

### 2.1. Search Strategy

The following search was conducted in PubMed from inception to 20 April 2021: (*Acinetobacter* [ti] OR baumannii [ti] OR “*Acinetobacter*” [Mesh] OR “*Acinetobacter baumannii*” [Mesh]) AND (synerg* [ti] OR combin* [ti] OR “Drug Combinations” [Mesh] OR “Drug Synergism” [Mesh] OR “Drug Therapy, Combination” [Mesh]). The same search, without the MESH terms, was also conducted in Scopus.

### 2.2. Eligibility Criteria

Any study (including in vitro, animal models, and clinical studies) evaluating the activity of antimicrobial combinations against clinical *A. baumannii* isolates was eligible, provided that the *A. baumannii* isolates tested were resistant to all components of the antimicrobial combinations assessed. The following exclusion criteria were applied: (1) studies including only noneligible isolates (see below definition for eligibility), (2) studies including both eligible and noneligible isolates, but not possible to extract data for eligible isolates, (3) combinations of antimicrobials with adjuvant, nonantibiotic agents, or with investigational agents (not currently in use for the treatment of infections). (4) Clinical studies without any information on synergy. (5) Studies written in languages other than English (little impact [21,22], often at higher risk of bias [23], and data extraction can be inaccurate [23]). Deduplication and screening for eligibility of the retrieved articles was conducted by the first author using the Rayyan online platform [24].

### 2.3. Data Extraction

The following data were extracted from each eligible article: country where the study was conducted, number of participating hospitals, methods of synergy testing (readers are referred to relevant references for a more detailed overview of the different methods [19,20,25,26,27]), list of antimicrobials tested for synergy, number of eligible strains (as defined below), number of eligible strains against which each combination demonstrated synergy and antimicrobial concentrations achieving synergy. Data were extracted by the first author in duplicate.

### 2.4. Definition of Eligible Strains

*A. baumannii* isolates were eligible for this review if resistant to all components of the antimicrobial combinations tested. The following breakpoints were used to define resistance based on CLSI [28] or EUCAST [29] clinical breakpoints (whichever was higher): amikacin > 32 mg/L, ampicillin-sulbactam > 16/8 mg/L, cefepime > 16 mg/L, cefiderocol > 8 mg/L, ceftazidime > 16 mg/L, ciprofloxacin > 2 mg/L, colistin > 2 mg/L, gentamicin > 8 mg/L, imipenem > 4 mg/L, levofloxacin > 4 mg/L, meropenem > 8 mg/L, minocycline > 8 mg/L, piperacillin > 64 mg/L, piperacillin/tazobactam > 64/4 mg/L, polymyxin B > 2 mg/L, tobramycin > 8 mg/L, trimethoprim-sulfamethoxazole > 2/38 mg/L. For antibiotics without established breakpoints by either EUCAST or CLSI the following cut-offs were applied: azithromycin > 4 mg/L (based on CLSI breakpoints for *Staphylococci* [12,28]), aztreonam >16 mg/L (based on breakpoints for *P. aeruginosa* [28,29]), cefoperazone/sulbactam > 32/16 mg/L [30], ceftazidime/avibactam > 8/4 mg/dl (based on breakpoints for *P. aeruginosa* [28,29]), chloramphenicol > 16 mg/L (based on breakpoints for Enterobacterales [28]), fosfomycin > 32 mg/L (based on EUCAST breakpoints for Enterobacterales and *Staphylococcus* spp [29]), fusidic acid > 1 mg/L (based on EUCAST breakpoints for *Staphylococcus* spp [29]), moxifloxacin > 0.25 mg/L (based on EUCAST breakpoints for Enterobacterales [29]), plazomicin > 4 mg/L (FDA interpretive criteria for Enterobacteriaceae [31]), rifampicin > 2 mg/L (based on CLSI breakpoints for *Staphylococci* [28], although much lower cut-offs have been proposed for *A. baumannii* [32]), tigecycline > 2 mg/L [33], trimethoprim > 8 mg/L (based on CLSI breakpoints for Enterobacterales [28]), vancomycin > 20 mg/L (based on clinically achievable concentrations [34,35,36], noting that the CLSI breakpoints for coagulase-negative *Staphylococci* is > 16 mg/L [28]).

### 2.5. Evaluation of In Vivo Feasibility of the Identified Combinations

In vivo feasibility of each synergistic combination was assessed based on the following: (1) synergy present in vitro at concentrations equal to or lower than established breakpoints of resistance (as defined above) for all antimicrobials used in the combination, or (2) synergy demonstrated in dynamic drug concentration-time experiments (such as the hollow-fiber infection model, or animal infection models) simulating the pharmacokinetics of human treatment regimens, or (3) clinically-achievable synergy based on pharmacokinetic/pharmacodynamic (PK/PD) modelling and Monte Carlo simulations [37].

### 2.6. Data Synthesis and Analysis

A qualitative synthesis of the data was conducted. Meta-analysis of the data was not pursued (a post hoc decision), based on the following findings of the review; methodological heterogeneity in synergy testing methods and interpretation, small number of studies and eligible isolates per combination, clonal relatedness of *A. baumannii* isolates from single-center studies, potential differences between different *A. baumannii* strains (i.e., synergy against *A. baumannii* strains isolated from one institution does not necessarily predict synergy against different strains, with different mechanisms and level of resistance), potential for publication bias (studies with negative results are less likely to be published), selective performance of more cumbersome synergy testing methods (such as time-kill assay or animal models) only against strains for which synergy had been demonstrated by other methods (such as checkerboard), questionable clinical relevance of synergy in many studies (synergy present only at high antimicrobial concentrations, likely not relevant for in vivo use, or at unclear concentrations).

## 3. Results

### 3.1. Summary and Characteristics of Reviewed Studies

**Figure 1:**
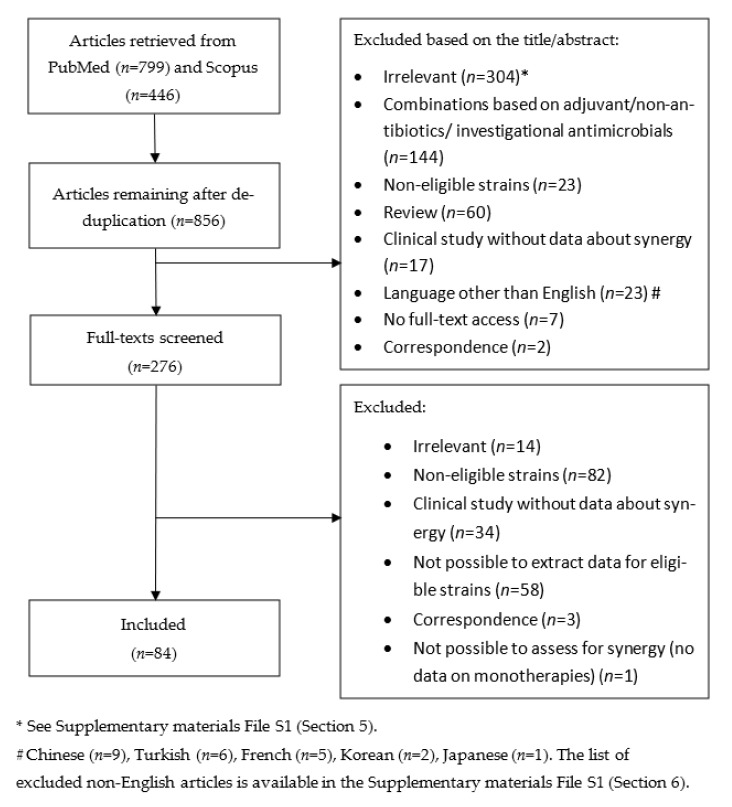
Flow chart of the review.

A flow chart of the review is depicted in Figure 1. Eighty-four relevant publications [12,35,36,37,38,39,40,41,42,43,44,45,46,47,48,49,50,51,52,53,54,55,56,57,58,59,60,61,62,63,64,65,66,67,68,69,70,71,72,73,74,75,76,77,78,79,80,81,82,83,84,85,86,87,88,89,90,91,92,93,94,95,96,97,98,99,100,101,102,103,104,105,106,107,108,109,110,111,112,113,114,115,116,117] were retrieved including 818 eligible *A. baumannii* isolates. The characteristics of the reviewed studies are summarized in the Supplementary Materials File S1 (Section 2). Most (73%) studies were published in the last 10 years, while about a third (35%) were published in the last 5 years (Appendix A, Table A1). Most studies were conducted in the European region (33%), America (29%) and the Western-Pacific region (24%) (Appendix A, Table A2). The number of eligible isolates per study was small in most studies, with most (79%) of them including ≤ 10 isolates (Supplementary Materials File S1 Section 2.4). Finally, most studies were single center (65%) and of the multicenter studies most (58%) were conducted in only two to five centers (Supplementary Materials File S1 Section 2.5), an important consideration as this reflects the clonal diversity of the *A. baumannii* isolates available for each study.

### 3.2. Overview of Methods for Assessment of Antimicrobial Combinations

A variety of methods were used for in vitro evaluation of antimicrobial combinations; disk diffusion methods (*n* = 4 studies, *n* = 18 eligible isolates), gradient strip methods (*n* = 11 studies, *n* = 229 eligible isolates), MIC determination by agar dilution (*n* = 2 studies, *n* = 42 eligible isolates), checkerboard assay (*n* = 44 studies, *n* = 599 eligible isolates), the multiple-combination bactericidal test (*n* = 1 study, *n* = 9 eligible isolates), time-kill assay (*n* = 51 studies, *n* = 259 eligible isolates), dynamic in vitro PK/PD models with antimicrobial concentrations simulating human treatment regimens (*n* = 6 studies, *n* = 10 isolates), and semi-mechanistic PK/PD modelling based on TKA data (*n* = 5 studies [37,54,102,107,118]). Finally, a few in vivo animal models (*n* = 11 studies, *n* = 18 isolates) eligible for review have been published [35,38,55,64,70,90,94,98,102,105,113]. No eligible clinical studies were retrieved.

### 3.3. Overview of Antimicrobial Combinations That have been Evaluated

Numerous different combinations (*n* = 141 double and *n* = 9 triple combinations) were evaluated predominantly based on polymyxins, rifamycins (predominantly rifampicin and recently rifabutin), sulbactam, fosfomycin and carbapenems. However, there were few available studies for most combinations with only 10 combinations having >3 studies available. Summarizing Tables of the number of studies and number of eligible isolates for each combination, as well as methods used to evaluate each combination are available in the Supplementary Materials File S1 (Section 3).

### 3.4. Overview of Polymyxin-Based Combinations

Polymyxin-based combinations were the most studied, with several studies demonstrating synergy against eligible *A. baumannii* isolates by combinations of polymyxins (either colistin or polymyxin-B) with cell-wall acting agents including: sulbactam (either alone or as ampicillin-sulbactam), beta-lactams (predominantly carbapenems, but also third generation cephalosporins, aztreonam, and ceftazidime/avibactam), glycopeptides (predominantly vancomycin, but also teicoplanin), and daptomycin. Furthermore, several studies have reported synergy between colistin and rifamycins against eligible strains (predominantly rifampicin and recently rifabutin). Isolated reports have also demonstrated synergy with trimethoprim/sulfamethoxazole, chloramphenicol, and fusidic acid.

The following triple polymyxin-based combinations have also been shown to be synergistic against selected eligible strains: polymyxin-B/meropenem/sulbactam [51,69], polymyxin-B/meropenem/ampicillin/sulbactam [61,62], colistin/doripenem/sulbactam [82], polymyxin-B/meropenem/fosfomycin [51,69] and polymyxin-B/doripenem/vancomycin [35]. Triple polymyxin-based combinations appear to be more active than double combinations and more likely to prevent regrowth during treatment [51,61,69,82], likely by preventing emergence of resistant subpopulations [61].

A variety of the above combinations (colistin/sulbactam, polymyxin-b/sulbactam, colistin/imipenem, colistin/meropenem, polymyxin-B/meropenem, colistin/doripenem, colistin/tigecycline, colistin/rifampicin, polymyxin-B/rifampicin, colistin/vancomycin, polymyxin-B/vancomycin, colistin/daptomycin, colistin/trimethoprim/sulfamethoxazole, colistin/chloramphenicol, colistin/fusidic acid, colistin/levofloxacin, polymyxin-B/fosfomycin/meropenem, polymyxin-B/sulbactam/meropenem, polymyxin-B/ampicillin/sulbactam/meropenem, colistin/sulbactam/doripenem, colistin/vancomycin/doripenem) have been shown to be synergistic at concentrations equal to or less than established breakpoints by a variety of methods, or in dynamic drug concentration-time experiments including animal models (Appendix A; Table A3, Table A4 and Table A5, and Supplementary Materials File S1 Section 4). Nevertheless, the number of studies and eligible isolates per combination was small and most combinations were active at clinically relevant concentrations only against selected of the tested eligible strains (Appendix A; Table A3, Table A4 and Table A5, and Supplementary Materials File S1 Sections 3–4).

### 3.5. Overview of Non-Polymyxin Based Combinations

Non-polymyxin-based combinations are predominantly based on combinations of the following antimicrobials (Supplementary Materials File S1 Section 3): sulbactam (either as sulbactam alone or in the form of ampicillin/sulbactam or cefoperazone/sulbactam), fosfomycin, rifampicin and carbapenems. However, a variety of other antimicrobials have been tried in combination regimens including aminoglycosides, tetracyclines (doxycycline, tigecycline, minocycline and eravacycline), fluoroquinolones, cephalosporins, aztreonam, trimethoprim/sulfamethoxazole, linezolid, teicoplanin and azithromycin.

The best data for non-polymyxin-based combinations come from four studies by Mohd Sazly Lim S et al. [37,44,45,118]. Fosfomycin/sulbactam (FOF/SUL), fosfomycin/meropenem (FOF/MEM), sulbactam/meropenem (SUL/MEM), fosfomycin/rifampin (FOF/RIF) and meropenem/rifampin (MEM/RIF) were evaluated for synergy against 50 eligible *A. baumannii* isolates characterized by high genetic diversity. The combinations were first evaluated by checkerboard assay [44]. Based on an FICI ≤ 0.5 the combinations were synergistic against 74% (FOF/SUL), 28% (FOF/MEM), 56% (SUL/MEM), 24% (FOF/RIF) and 20% (RIF/MEM) of eligible strains. Synergy was mostly detected at concentrations above established breakpoints of resistance. However, considering higher proposed breakpoints based on PK/PD models (32 mg/L for SUL [119,120] and 128 mg/L for FOF [45,121]) the combination FOF/SUL was active against 18 of 28 (64%) eligible isolates [37], the combination FOF/MEM was active against 9 of 33 (27%) eligible isolates [45], and the combination SUL/MEM was active against 9 of 46 (20%) eligible isolates [118]. FOF/SUL and SUL/MEM were further evaluated in TKA against selected isolates [37,44,118], but synergy was only reported at concentrations (128/128 mg/L for SUL/FOF and 64/32–128/64 for SUL/MEM) higher than established breakpoints.

Finally, Mohd Sazly Lim S et al. evaluated two of the above combinations with semi-mechanistic PK/PD modelling; FOF/SUL (simulated regimen: 8 g of fosfomycin given every 8 h as a 1 h infusion and 4 g of sulbactam given every 8 h as a 4 h infusion) [37] and SUL/MEM (simulated regimen: 2 gr of meropenem given every 8 h as a 3 h infusion, and 4 g of sulbactam given every 8 h as a 4 h infusion [118]). A high probability of target attainment was shown for FOF/SUL against the selected isolate (FOF MIC 2048, SUL MIC 128, combination MIC in checkerboard 32/16 mg/L); 81.6%, 76.4%, and 71.6% for stasis, 1-log_10_ kill and 2-log_10_ kill, respectively (compared to 23.3%, 19.8% and 15.5% for fosfomycin monotherapy, and 53.5%, 46.5%, and 32.5% for sulbactam monotherapy) [37]. In contrast, the probability of target attainment was at best moderate for SUL/MEM against the selected isolates (MEM MIC 128 mg/L, SUL MIC 256 mg/L, combination MICs 8/64 and 8/32 mg/L); 41%, 38% and 34% for stasis, 1-log_10_ kill and 2-log_10_ kill, respectively (compared to no killing with either of the monotherapies) [118].

Avibactam/sulbactam is another recently proposed promising combination. Rodriguez CH et al. [47] showed that avibactam at a fixed concentration of 4 mg/L reduced the MIC of sulbactam to ≤4 mg/L in all 35 non-metallo-β-lactamase (MBL)-producing sulbactam-resistant *A. baumannii* isolates in one study. The activity of sulbactam/avibactam (and to a lesser extent of sulbactam/relebactam) was also confirmed in a subsequent study [122]. The rationale of the combination is that avibactam may inhibit the β-lactamases that affect activity of sulbactam [47]. However, the combination is less effective against metallo-β-lactamase-producing isolates [47,122].

In contrast to non-MBL Enterobacterales [6], double carbapenem combinations are less likely to be clinically relevant for *A. baumannii* strains. Specifically, the combination meropenem/imipenem was synergistic against 6 of 21 eligible isolates according to checkerboard assay in one study, but synergy was only observed at concentration above established breakpoints of resistance (synergy was present at the following meropenem/imipenem concentrations: 16/4, 16/8, 32/16 and 32/32, 16/8 mg/L) and all isolates had relatively low MICs (mostly 32–64 mg/L) [46]. The combination imipenem/meropenem has also been shown to be effective in a murine intraperitoneal infection model (using two *A. baumannii* strains with meropenem-imipenem MICs 16–16 and 32–32 mg/L, respectively), but mortality and bacterial clearance were similar comparing meropenem monotherapy to combination therapy [38]. Additionally, the combination imipenem/ertapenem was not found to be synergistic in another study [73].

### 3.6. Evaluation of Clinical Relevance of Reported Synergy

Detailed data regarding the proportion of observed synergy for each combination (per study and method) and assessment of the clinical relevance are available in the Supplementary Materials File S1 (Section 4). In most cases, synergy was only reported at antimicrobial concentrations above the established breakpoints of resistance or the concentration at which synergy was observed was not reported. Specifically, of *n* = 539 cases of reported synergy in checkerboard assay, synergy was observed at concentrations ≤breakpoints in only 112 (21%) cases, synergy was reported at concentration >breakpoints in 194 (36%) cases, while in 233 (43%) cases the concentration at which synergy was present was unclear. Similarly, of *n* = 185 cases of reported synergy in TKA, synergy was observed at concentrations ≤breakpoints in only 65 (35%) cases, synergy was reported at concentration >breakpoints in 88 (48%) cases, while in 32 (17%) cases the concentration at which synergy was present was unclear.

Additionally, the clinical relevance of improved outcomes (survival, reduction of bacterial loads, sterilization of cultures) in animal models is unclear, despite simulation of human treatment regimens, considering the unexpectedly high efficacy of monotherapies in many cases [38,90,94,98,105,113], and potentially nonrelevant for humans mechanisms of action of antimicrobials [35]. Finally, dynamic in vitro PK/PD models [61,62,73,87,88,107] and semi-mechanistic PK/PD models were available for only a few combinations and selected isolates [37,54,102,107,118] but provided useful information about the killing activity of antimicrobial combinations at clinically relevant concentrations.

A summary of combinations that have been found synergistic at concentrations ≤established breakpoints of resistance are available in Table A3 of Appendix A. Studies using dynamic in vitro PK/PD models or animal models are summarized in Table A4 and Table A5 of Appendix A.

### 3.7. Clinical Studies

Although several studies have assessed antimicrobial combination in *A. baumannii* infections (e.g., [7,8,9,10,11,123,124]) none was eligible for this review for the following reasons: (a) combinations were assessed in patients with noneligible isolates (i.e., isolates susceptible to at least one component of the combination) or the extraction of data for eligible isolates was not possible, and/or (b) lack of in vitro evaluation for the presence of synergy. The latter is important because, as demonstrated in this review, in vitro synergy observed against selected *A. baumannii* strains with a specific combination cannot be generalized to other *A. baumannii* strains. Furthermore, the very few available studies including patients with infections by PDRAB [1,6,124,125] have major limitations, including small study populations, retrospective designs, lack of a control group or direct comparison of different treatment regimens, and lack of correlation of in vitro susceptibility testing of the combinations with outcomes.

Notable among the available studies is a secondary analysis of the AIDA study (a randomized controlled trial comparing colistin monotherapy to colistin-meropenem combination in patients with carbapenem-resistant Gram-negative infections [9]) comparing monotherapy to combination therapy against colistin- and carbapenem-resistant *A. baumannii* infections [10]. Based on this study, the colistin-meropenem combination was paradoxically associated with higher mortality compared to monotherapy [10]. However, being an exploratory subgroup analysis, the study has several limitations and data on the presence (or absence) of synergy were not reported for the subgroup of patients with colistin- and carbapenem-resistant *A. baumannii* infections. Nevertheless, the study raises the hypothesis that blindly (in the absence of clinical data) using antimicrobial combinations could unexpectedly result in worse outcomes.

In contrast, favorable results have been reported in a few small series (with all the above-mentioned limitations) with selected combinations. For example, triple combination therapy with high-dose ampicillin/sulbactam, high-dose tigecycline and colistin in patients with ventilator-associated pneumonia by PDRAB resulted in clinical cure in 9 of 10 patients [125]. Similarly, in another series, all seven patients with ventilator-associated pneumonia or bacteremia by colistin-resistant *A. baumannii* were successfully treated with a triple combination including colistin, doripenem and ampicillin/sulbactam (although with one exception, all isolates had ampicillin/sulbactam MICs ≤ 16/8 mg/L, i.e., were not eligible for this review) [126]. Furthermore, the combination of colistin with rifampicin has been used successfully to treat post-neurosurgical meningitis after emergence of colistin resistance during treatment with colistin monotherapy [127,128]. However, eligibility of the included isolates in the latter studies could not be assessed due to lack of reporting of rifampicin MICs [127,128].

Therefore, clinical studies assessing antimicrobial combinations in infections by PDRAB are urgently needed. The selection of antimicrobial combinations for further clinical study should ideally be guided by in vitro susceptibility testing of the combinations against local *A. baumannii* strains, taking into account whether synergy is achievable at clinically relevant concentrations.

## 4. Discussion

### 4.1. Summary of Main Findings

The emergence of XDR/PDR *A. baumannii* [1], which is associated with high mortality [2] and limited treatment options [6], has resulted in an increasing number of publications evaluating the role of antimicrobial combination therapy. A vast number of potential combinations has been reported, although most combinations have been evaluated only against a limited number of eligible *A. baumannii* isolates. The most studied combinations are polymyxin-based combinations with cell-wall acting agents (including sulbactam, carbapenems and vancomycin), rifampicin and fosfomycin. Nevertheless, a variety of combinations have been reported to be synergistic at clinically achievable concentrations, at least against selected *A. baumannii* isolates. However, in most cases synergy was reported either at too high concentrations or at unclear concentrations.

### 4.2. Polymyxin-Based Combinations

Polymyxin-based combinations were originally proposed to prevent treatment failure due to the emergence of polymyxin-resistant *A. baumannii* during therapy [129], but may actually be most useful for PDRAB [5,125,127,128]. A proposed mechanism to explain the synergy between polymyxins and other antimicrobials is that polymyxins, even at subinhibitory concentrations, may increase the permeability of *A baumannii*’s cell wall to other antimicrobials, including antimicrobials that would otherwise be ineffective against Gram-negative pathogens (such as glycopeptides and lipopeptides) [12,34,56,88].

Polymyxins may be combined, either as double or as triple combinations, with a variety of antimicrobials, including carbapenems, sulbactam, fosfomycin, rifampicin, rifabutin (which has recently been shown to be much more potent than rifampicin [130] and may retain activity even against PDRAB [131]) and vancomycin. Synergy with many of these combinations was achievable at concentrations ≤established breakpoints of resistance and demonstratable in animal models and/or dynamic in vitro PK/PD studies simulating human treatment regimens.

However, synergy is not universal and not applicable to every *A. baumannii* strain. Clinically relevant synergy may be less likely for strains with very high MICs. For example, clinically-relevant synergy between polymyxins and carbapenems appears to be less likely for isolates with high carbapenem MIC (doripenem >64 mg/L [82], meropenem ≥64 mg/L [132]). Triple combinations may be more effective than double combinations, by lowering MICs of individual agents to even lower levels and preventing emergence of resistance during treatment [51,61,69,82].

### 4.3. Non-Polymyxin Combinations

A variety of non-polymyxin combinations have been reported, predominantly involving the following antimicrobials: carbapenems, fosfomycin, sulbactam and rifamycins. The combination fosfomycin/sulbactam and to a lesser extent meropenem/sulbactam are especially promising and most studied [37,44,118], but a variety of other combinations have been found synergistic against selected eligible *A. baumannii* isolates. Such combinations may be even more active as triple combinations with polymyxins [51,61,69,82]. Furthermore, among non-polymyxin combinations, the recently proposed avibactam/sulbactam combination (aiming to restore susceptibility to sulbactam by inhibition of non-MBL β-lactamases with avibactam) is particularly promising and warrants further study [47,122].

Tigecycline-based combinations are often used in clinical practice against PDRAB [124,133], probably because of MICs closer to the cut-off for susceptibility [12]. However, based on the limited available data, tigecycline-based (or other tetracyclines, including eravacycline and minocycline) combinations are seldomly synergistic against resistant *A. baumannii* strains at clinically achievable concentrations [12,53,63,71,77,89,96,103,104,117]. However, the lack of in vitro synergy does not preclude a role for tigecycline in the treatment of XDR/PDR *A. baumannii*, especially with higher dose regimens that are predicted to achieve PK/PD targets for isolates with MICs up to 4–8 mg/L [134].

### 4.4. Limitations of the Review and of the Available Evidence

Despite the abundance of in vitro studies evaluating a variety of antimicrobial combinations against XDR/PDR *A. baumannii*, in vivo data, PK/PD models and clinical data are still limited. Furthermore, there is no acceptable gold standard method (one that best predicts in vivo efficacy) for the in vitro evaluation of synergy, mainly due to the lack of studies correlating in vitro synergy to clinical outcomes [19], and the results of different methods are often conflicting [25,68].

Moreover, as demonstrated in this review, studies often fail to assess the clinical relevance of reported synergy, as evidenced by the evaluation for synergy at antimicrobial concentration unlikely to be clinically relevant or lack of reporting of concentrations at which synergy is present. For example, an FIC index ≤ 0.5 in checkerboard assay does not necessarily prove clinically relevant synergy if antimicrobials are synergistic at concentrations higher than those achievable in vivo at the site of the infection. Similarly, in time-kill assays antimicrobials should ideally be used in concentrations achievable at the site of infection [20], which is often not the case as demonstrated in this review.

However, although clinically-relevant synergy was defined as synergy achievable at concentrations ≤ breakpoints of resistance it should be acknowledged that potentially higher breakpoints have been estimated (based on PK/PD data and Monte Carlo simulations) for high-dose, prolonged-infusion regimens [6]. For example, a high probability of target attainment with such regimens has been reported up to the following maximum MICs: meropenem ≤128 mg/L [135], doripenem ≤8 mg/L [136], fosfomycin ≤128 mg/L [45,121], sulbactam ≤32 mg/L [119,120]. Furthermore, some studies have evaluated the feasibility of synergistic combinations based on maximum clinically achievable concentrations [44,59] but we believe this approach could result in overestimating the in vivo relevance of synergistic combinations. Finally, the clinical relevance of synergy in animal models, even when using dosing regimens simulating human pharmacokinetics, is unclear considering that in some studies high efficacy was seen even for monotherapies against resistant strains [38,113], while in some cases antimicrobials may have additional functions in animal models not relevant to humans [35].

Finally, another major limitation of this review is the limited clonal diversity of eligible *A. baumannii* isolates for most combinations evaluated, considering that most studies were single-center and that for most combinations only few eligible isolates were assessed. This, combined with the inconsistent activity of antimicrobial combinations highlight the need to confirm in vitro synergy against local *A. baumannii* strains before using any of these combinations in clinical practice.

### 4.5. Strengths of the Review

Despite the above limitations, this is an exhaustive review of antimicrobial combination options against PDRAB, aiming to aid clinicians, researchers and microbiology laboratories to prioritize the selection of the most promising combinations for further evaluation against PDRAB. Furthermore, a detailed assessment of the potential clinical relevance of each synergistic combination was conducted, based on the concentrations that synergy was observed and the availability of PK/PD or animal models.

## 5. Conclusions

Antimicrobial combinations may be the only treatment option against PDR *A. baumannii*. Numerous combinations have been evaluated and several appear to be active at clinically relevant concentrations, at least against selected eligible *A. baumannii* isolates. However, studies often do not report the concentrations at which synergy is observed or use antimicrobials at concentrations unlikely to be clinically relevant. This is an important limitation of the available literature and an important consideration for future studies evaluating antimicrobial combinations against PDRAB. Furthermore, no combination was consistently synergistic against all isolates evaluated. Therefore, selecting the most appropriate combination is likely strain-specific and should be guided by in vitro synergy evaluation. Combinations demonstrating activity at clinically relevant concentrations and/or supported by PK/PD data and animal models should be further evaluated in appropriately designed clinical studies, which are currently lacking.

## Data Availability

The summary of characteristics and findings of each study included in this review is available in the Supplementary Materials File S1.

## Supplementary Materials

The following are available online at https://www.mdpi.com/article/10.3390/antibiotics10111344/s1, File S1: Table of Contents.

## Appendix A

**Table A1:** Distribution of studies by year of publication.

Year of Publication	Number of Studies (%)
**2017–2021**	**29 (35%)**
2021	5 (6%)
2020	6 (7%)
2019	10 (12%)
2018	3 (4%)
2017	5 (6%)
**2012–2016**	**32 (38%)**
2016	10 (12%)
2015	6 (7%)
2014	7 (8%)
2013	6 (7%)
2012	3 (4%)
**2007–2011**	**15 (18%)**
2011	3 (4%)
2010	6 (7%)
2009	3 (4%)
2008	2 (2%)
2007	1 (1%)
**2002–2006**	**7 (8%)**
2005	2 (2%)
2004	4 (5%)
2003	1 (1%)
**1995–2001**	**1 (1%)**
1996	1 (1%)

**Table A2:** Distribution of studies by country and WHO regions.

WHO Regions	Number of Studies Per Region (%)
**Americas**	**24 (29%)**
Brazil	6 (7%)
USA	12 (14%)
Argentina	3 (4%)
Colombia	1 (1%)
**Southeast Asia Region**	**7 (8%)**
India	1 (1%)
Thailand	6 (7%)
**European Region**	**28 (33%)**
France	3 (4%)
Germany	1 (1%)
Greece	3 (4%)
Italy	3 (4%)
Spain	7 (8%)
Turkey	7 (8%)
Switzerland	1 (1%)
United Kingdom	1 (1%)
**Eastern Mediterranean Region**	**5 (6%)**
Iran	1 (1%)
Saudi Arabia	4 (5%)
United Arab Emirates	2 (2%)
Oman	2 (2%)
Kuwait	2 (2%)
Qatar	2 (2%)
Bahrain	3 (4%)
**Western Pacific Region**	**20 (24%)**
China	9 (11%)
South Korea	6 (7%)
Taiwan	3 (3%)

**Table A3:** Antimicrobial combinations shown to be synergistic in checkerboard (CHBD) and/or time-kill assay (TKA) at concentrations ≤ established breakpoints of resistance.

Antimicrobial Combinations	CHBD			CHBD: Concentration at which Synergy Was Present			TKA			TKA: Concentration at which Synergy Was Present		
	Studies *n*	Isolates *n*	Synergy *n* (% *)	≤Breakpoints *n* (% *)	>Breakpoints *n* (% *)	Unclear *n* (% *)	Studies *n*	Isolates *n*	Synergy *n* (% *)	≤Breakpoints *n* (% *)	>Breakpoints *n* (% *)	Unclear *n* (% *)
**SUL-based**												
SUL/CAZ	1	10	7 (70%)	1 (10%)	6 (60%)	0 (0%)						
SUL/CIP	1	10	8 (80%)	2 (20%)	6 (60%)	0 (0%)						
SUL/MEM	6	173	72 (42%)	2 (1%)	2 (1%)	68 (39%)	3	54	32 (59%)	0 (0%)	7 (22%)	25 (78%)
SUL/DOR							1	17	4 (24%)	4 (100%)	0 (0%)	0 (0%)
SUL/AVI							1	1	1 (100%)	1 (100%)	0 (0%)	0 (0%)
SUL/GEN	1	10	8 (80%)	2 (25%)	6 (75%)	0 (0%)						
SUL/CST							1	6	2 (33%)	2 (100%)	0 (0%)	0 (0%)
SUL/PMB	1	3	2 (67%)	2 (100%)	0 (0%)	0 (0%)						
SUL/FOF	2	56	41 (73%)	3 (7%)	37 (90%)	1 (2%)	2	10	7 (70%)	0 (0%)	7 (100%)	0 (0%)
**SAM-based**												
SAM/FEP	1	2	2 (100%)	1 (50%)	1 (50%)	0 (0%)						
SAM/LVX	1	7	7 (100%)	5 (71%)	2 (29%)	0 (0%)						
SAM/MEM	2	10	2 (20%)	2 (100%)	0 (0%)	0 (0%)	1	2	0 (0%)	0 (0%)	0 (0%)	0 (0%)
SAM/RIF	1	7	7 (100%)	4 (57%)	1 (14%)	2 (29%)						
**IMP-based**												
IMP/CFS	1	16	11 (69%)	9 (82%)	2 (18%)	0 (0%)						
IMP/CST	2	10	9 (90%)	2 (20%)	7 (70%)	0 (0%)	1	2	0 (0%)	0 (0%)	0 (0%)	0 (0%)
IMP/RIF	2	28	16 (57%)	11 (39%)	3 (11%)	2 (7%)	5	13	6 (46%)	0 (0%)	6 (46%)	0 (0%)
IMP/FOF	3	45	26 (58%)	9 (20%)	10 (22%)	7 (16%)	1	9	8 (89%)	0 (0%)	8 (89%)	0 (0%)
**MEM-based**												
MEM/SUL	6	173	72 (42%)	2 (1%)	2 (1%)	68 (39%)	3	54	32 (59%)	0 (0%)	7 (13%)	25 (46%)
MEM/SAM	2	10	2 (20%)	2 (20%)	0 (0%)	0 (0%)	1	2	0 (0%)	0 (0%)	0 (0%)	0 (0%)
MEM/AMK	4	47	16 34%)	1 (2%)	2 (4%)	13 (28%)						
MEM/CST	6	29	21 (72%)	5 (17%)	11 (3%)	5 (17%)	3	4	4 (100%)	0 (0%)	4 (100%)	0 (0%)
MEM/PMB	1	3	3 (100%)	3 (100%)	0 (0%)	0 (0%)	1	2	0 (0%)	0 (0%)	0 (0%)	0 (0%)
MEM/FOF	4	79	15 (19%)	1 (1%)	14 (18%)	0 (0%)						
MEM/VAN	1	5	3 (60%)	1 (20%)	0 (0%)	2 (40%)						
**DOR-based**												
DOR/SUL							1	17	4 (24%)	4 (24%)	0 (0%)	0 (0%)
DOR/CST	3	6	2 (33%)	1 (17%)	1 (17%)	0 (0%)	5	33	23 (70%)	19 (58%)	4 (12%)	0 (0%)
DOR/TGC	1	3	3 (100%)	1 (33%)	0 (0%)	2 (67%)	1	45	5 (11%)	5 (11%)	0 (0%)	0 (0%)
DOR/RIF	1	1	0 (0%)	0 (0%)	0 (0%)	0 (0%)	1	5	2 (40%)	1 (20%)	1 (20%)	0 (0%)
**CZA- or AVI-based**												
AVI/SUL							1	1	1 (100%)	1 (100%)	0 (0%)	0 (0%)
**CST-based**												
CST/SUL							1	6	2 (33%)	2 (33%)	0 (0%)	0 (0%)
CST/LVX	2	2	1 (50%)	1 (50%)	0 (0%)	0 (0%)	2	2	1 (50%)	0 (0%)	1 (50%)	0 (0%)
CST/IMP	2	10	9 (90%)	2 (20%)	7 (70%)	0 (0%)	1	2	0 (0%)	0 (0%)	0 (0%)	0 (0%)
CST/MEM	6	29	21 (72%)	5 (17%)	11 (38%)	5 (17%)	3	4	4 (100%)	0 (0%)	4 (100%)	0 (0%)
CST/DOR	3	6	2 (33%)	1 (17%)	1 (17%)	0 (0%)	5	33	23 (70%)	19 (58%)	4 (12%)	0 (0%)
CST/TGC	2	10	0 (0%)	0 (0%)	0 (0%)	0 (0%)	1	12	7 (100%)	7 (100%)	0 (0%)	0 (0%)
CST/RIF	5	40	31 (78%)	10 (25%)	10 (25%)	11 (28%)	3	7	7 (100%)	0 (0%)	6 (86%)	1 (14%)
CST/SXT	2	8	2 (25%)	1 (13%)	0 (0%)	1 (13%)	1	1	1 (100%)	0 (0%)	1 (100%)	0 (0%)
CST/CHL	1	2	2 (100%)	1 (50%)	1 (50%)	0 (0%)	1	2	2 (100%)	0 (0%)	2 (100%)	0 (0%)
CST/FA	2	6	6 (100%)	1 (17%)	2 (33%)	3 (50%)	2	4	3 (75%)	0 (0%)	3 (75%)	0 (0%)
CST/VAN	7	33	29 (88%)	2 (6%)	2 (6%)	25 (67%)	6	20	16 (80%)	13 (65%)	3 (15%)	0 (0%)
**PMB-based**												
PMB/SUL	1	3	2 (67%)	2 (67%)	0 (0%)	0 (0%)						
PMB/MEM	1	3	3 (100%)	3 (100%)	0 (0%)	0 (0%)	1	2	0 (0%)	0 (0%)	0 (0%)	0 (0%)
PMB/RIF	1	3	3 (100%)	1 (33%)	1 (33%)	1 (33%)	1	3	1 (33%)	1 (33%)	0 (0%)	0 (0%)
PMB/VAN	1	3	3 (100%)	3 (100%)	0 (0%)	0 (0%)	1	3	2 (67%)	0 (0%)	2 (67%)	0 (0%)
**TGC-based**												
TGC/DOR	1	3	3 (100%)	1 (33%)	0 (0%)	2 (67%)	1	45	5 (11%)	5 (11%)	0 (0%)	0 (0%)
TGC/AMK	1	14	2 (14%)	1 (7%)	1 (7%)	0 (0%)	1	1	1 (100%)	1 (100%)	0 (0%)	0 (0%)
TGC/CST	2	10	0 (0%)	0 (0%)	0 (0%)	0 (0%)	1	12	7 (58%)	7 (58%)	0 (0%)	0 (0%)
TGC/RIF	2	16	1 (6%)	0 (0%)	1 (6%)	0 (0%)	2	4	1 (25%)	1 (25%)	0 (0%)	0 (0%)
TGC/FOF	1	4	3 (75%)	1 (25%)	2 (50%)	0 (0%)	1	1	1 (100%)	0 (0%)	1 (100%)	0 (0%)
**RIF-based**												
RIF/SAM	1	7	7 (100%)	4 (57%)	1 (14%)	2 (29%)						
RIF/CFS	1	7	2 (29%)	1 (14%	0 (0%)	1 (14%)						
RIF/IMP	2	28	16 (57%)	11 (39%)	3 (11%)	2 (7%)	5	13	6 (46%)	0 (0%)	6 (46%)	0 (0%)
RIF/DOR	1	1	0 (0%)	0 (0%)	0 (0%)	0 (0%)	1	5	2 (40%)	1 (20%)	1 (20%)	0 (0%)
RIF/CST	5	40	31 (78%)	10 (25%)	10 (25%)	11 (28%)	3	7	7 (100%)	0 (0%)	6 (85%)	1 (14%)
RIF/PMB	1	3	3 (100%)	1 (33%)	1 (33%)	1 (33%)	1	3	1 (33%)	1 (33%)	0 (0%)	0 (0%)
RIF/TGC	2	16	1 (100%)	0 (0%)	1 (100%)	0 (0%)	2	4	1 (25%)	1 (25%)	0 (0%)	0 (0%)
**FOF-based**												
FOF/SUL	2	56	41 (73%)	3 (5%)	37 (66%)	1 (2%)	2	10	7 (70%)	0 (0%)	7 (70%)	0 (0%)
FOF/IMP	3	45	26 (58%)	9 (20%)	10 (22%)	7 (16%0	1	9	8 (89%)	0 (0%)	8 (89%)	0 (0%)
FOF/MEM	4	79	15 (19%)	1 (1%)	14 (18%)	0 (0%)						
FOF/AMK	2	29	26 (90%)	11 (38%)	15 (52%)	0 (0%)						
FOF/GEN	2	13	12 (92%)	3 (32%)	9 (69%)	0 (0%)						
FOF/TGC	1	4	3 (75%)	1 (25%)	2 (50%)	0 (0%)	1	1	1 (100%)	0 (0%)	1 (100%)	0 (0%)
**Triple combinations**												
PMB/FOF/MEM	1	3	3 (100%)	3 (100%)	0 (0%)	0 (0%)						
PMB/SUL/MEM	1	3	3 (100%)	3 (100%)	0 (0%)	0 (0%)						
CST/DOR/SUL							1	6	6 (100%)	6 (100%)	0 (0%)	0 (0%)
CST/VAN/DOR							1	3	3 (100%)	3 (100%)	0 (0%)	0 (0%)

* Percentage over total number of isolates. Combinations only shown to be synergistic at concentrations > established breakpoints or at unclear concentrations are not included in this Table. A more complete (including all combinations) version of this Table as well as similar Tables for other methods are available in the Supplementary Materials File S1 Section 4. Abbreviations: AMK = amikacin, AVI = avibactam, ATM = aztreonam, AZM = azithromycin, CAZ = ceftazidime, CFS = cefoperazone/sulbactam, CHBD = checkerboard assay, CHL = chloramphenicol, CIP = ciprofloxacin, CST = colistin, CZA = ceftazidime/avibactam, DOR = doripenem, FA = fusidic acid, FEP = cefepime, FOF = fosfomycin, GEN = gentamicin, IMP = imipenem, LVX = levofloxacin, MEM = meropenem, PMB = polymyxin-B, RIF = rifampicin, SAM = ampicillin/sulbactam, SUL= sulbactam, SXT = trimethoprim/sulfamethoxazole, TEC = teicoplanin, TGC = tigecycline, TKA = time-kill assay, TMP = trimethoprim, VAN = vancomycin. In the single eligible study using multiple-combination bactericidal assay [12] (not shown in the Table) the following combinations were active at concentrations equal to breakpoints of resistance: SAM/RIF (synergistic against 1 of 8 eligible isolates), SAM/SXT (1/7), SAM/TEC (1/8), AMK/CAZ (1/6), AMK/SXT (1/6), AZM/CAZ (1/8), AZM/SXT (1/6), AZM/TEC (1/8), ATM/CAZ (1/9), ATM/SXT (1/7), ATM/TEC (1/9), CAZ/MEM (1/9), CAZ/RIF (1/9), CAZ/TGC (1/9), CAZ/SXT (1/7), CAZ/VAN (1/9), MEM/RIF (1/9), MEM/SXT (1/7), MEM/TEC (1/9), RIF/SXT (1/7), SXT/VAN (1/7), AMK/RIF (2/6), CAZ/TEC (2/9), CST/RIF (9/9), CST/TEC (9/9), CST/VAN (8/9), CST/MEM (8/9), CST/ATM (8/9), CST/CAZ (6/9), CST/SAM (5/8), CST/SXT (3/8), CST/AMK (4/6), CST/AZM (4/8).

**Table A4:** Studies using dynamic in vitro PK/PD models.

Study-Combinations	Method	Synergy % (*n*/N)	Comments
**Lenhard, J.R., 2017** [61,62]			
PMB/MEM	HFIM	0 (0/1)	Doses simulating human regimens were used (PMB 3.33 mg/kg then 1.43 mg/kg every 12 h, MEM 2 gr every 8 h as 3 h infusions, SAM 8/4 g every 8 h as 3 h infusions).
PMB/SAM		0 (0/1)	
MEM/SAM		0 (0/1)	
PMB/MEM/SAM		100 (1/1)	
**Yuan, Z., 2010** [102] **and Lim, T.P., 2008** [107]			
AMK/LVX	HFIM	0 (0/1)	Regrowth despite initial killing at 4 h.
AMK/FEP		0 (0/1)	Regrowth despite initial killing at 4 h.
**Córdoba, J., 2015** [73]			
CST/IMP	Other dynamic in vitro PK/PD model	0 (0/1)	Simulation of human treatment regimens
CST/DAP		100 (1/1)	
IMP/ETP		0 (0/3)	
RIF/CFS		0 (0/7)	
**Housman, S.T., 2013** [87]			Simulated regimens: SAM 9 g q8 h (3 h inf), DOR 2 gr q8 h (4 h inf), TGC 200 mg q12 h (0.5 h inf).
TGC/DOR	Other dynamic in vitro PK/PD model	0 (0/2)	
SAM/DOR		0 (0/3)	Increased killing with SAM/DOR vs. monotherapies against all 3 strains but with regrowth by 24 h.
SAM/TGC		0 (0/1)	
**Lee, H.J., 2013** [88]			
CST/RIF	Other dynamic in vitro PK/PD model	100 (1/1)	Regimens mimicking human serum concentration after usual doses in critically-ill patients.

Abbreviations: AMK = amikacin, CFS = cefoperazone/sulbactam, CHBD = checkerboard assay, CST = colistin, CZA = ceftazidime/avibactam, DAP = daptomycin, DOR = doripenem, ETP = ertapenem, FEP = cefepime, FOF = fosfomycin, HFIM = hollow-fiber infection model, IMP = imipenem, LVX = levofloxacin, MEM = meropenem, *n*/N = number of isolates against which synergy was demonstrated/total number of eligible isolates, PK/PD = pharmacokinetic/pharmacodynamic, PMB = polymyxin-B, RIF = rifampicin, SAM = ampicillin/sulbactam, TGC = tigecycline.

**Table A5:** Studies using animal models.

Study-Combinations	Method	Synergy % (*n*/N)	Comments
**Cebrero-Cangueiro, T., 2021** [38]			
MEM/IMP	Intraperitoneal infection mouse model	0 (0/2)	Decreased bacterial loads with combination vs. monotherapy, but similar mortality and bacterial clearance comparing meropenem monotherapy to combination therapy.
**Poulakou, G., 2019** [55]			
CST/DAP	Intraperitoneal infection mouse model	100 (1/1)	The combination significantly improved survival and reduced bacterial loads in tissues compared to monotherapies.
**Wei, W., 2017** [64]			
CST/LVX	*G. mellonella* model	0 (0/1)	Same survival comparing combination therapy to monotherapy
**Yang, H., 2016** [70]			
CST/VAN	*G. mellonella* model	100 (1/1)	Survival rate in *G. mellonella* model higher with combination, but high survival even with monotherapies.
**O’Hara, J.A., 2013** [35]			
CST/DOR	*G. mellonella* model	0 (0/3)	No synergy
CST/VAN		0 (0/3)	
DOR/VAN		100 (3/3)	The clinical relevance of the *G. mellonella* model is unclear because of mechanisms of action likely not relevant to humans; high survival even with DOR and VAN monotherapies, and high survival with DOR/VAN despite lack of in vitro synergy
CST/VAN/DOR		100 (3/3)	
**Queenan, A.M., 2013** [90]			
DOR/CIP	intraperitoneal infection mouse model	0 (0/1)	No synergy
DOR/LVX		100 (1/1)	Improved survival in the mouse model (the isolate had relatively low MICs: DOR 16 mg/L and LVX 8 mg/L).
**Pachón-Ibáñez, M.E., 2011** [94]			
RIF/IMP	Pneumonia mouse model	0 (0/2)	In the animal model survival with RIF/IMP (80 and 33%) and RIF/SUL (60 and 53%) did not differ significantly compared to RIF monotherapy (73 and 40%). Lung clearance and blood culture sterilization was higher against one of the two strains with RIF/SUL.
RIF/SUL		50 (1/2)	
**Pachón-Ibáñez, M.E., 2010** [98]			
SUL/IMP	Pneumonia (mouse) and meningitis (rabbit) models	100 (1/1)	Higher survival and bacterial clearance in animal model compared to monotherapies.
RIF/IMP		0 (0/1)	Survival not improved comparing RIF monotherapy (71%) to combination therapy (60%), despite improved bacterial clearance.
RIF/SUL		0 (0/1)	Survival not improved comparing RIF monotherapy (71%) to combination therapy (47%), despite improved bacterial clearance.
**Yuan, Z., 2010** [102]			
AMK/LVX	Pneumonia mouse model	0 (0/1)	Similar survival with AMK monotherapy.
AMK/FEP		1 (1/1)	Improved survival and reduction of tissue bacterial burden in the mouse model.
FEP/LVX		0 (0/1)	Similar survival with FEP monotherapy.
**Song, Y.C., 2009** [105]			
IMP/RIF	Pneumonia mouse model	100 (3/3)	Synergistic (≥2Δlog reduction in lung baterial loads compared to RIF monotherapy) against all 3 strains, but 100% survival with both monotherapy and combination.
RIF/AMK		0 (0/1)	Not better than monotherapy
IMP/AMK		0 (0/1)	Not better than monotherapy
**Montero, A., 2004** [113]			
IMP/RIF	Pneumonia mouse model	50 (1/2)	Strain D: no differences compared to monotherapy in the mouse model. Strain E: significantly reduced lung bacterial counts, no significant reduction of bacteremia, similar survival (100% with the combination, 100% with RIF monotherapy).

Abbreviations: AMK = amikacin, CIP = ciprofloxacin, CST = colistin, DOR = doripenem, FEP = cefepime, IMP = imipenem, LVX = levofloxacin, MEM = meropenem, RIF = rifampicin, SUL = sulbactam, VAN = vancomycin.

## References

[1] Karakonstantis S., Kritsotakis E.I., Gikas A. (2019). Pandrug-resistant Gram-negative bacteria: A systematic review of current epidemiology, prognosis and treatment options. J. Antimicrob. Chemother..

[2] Karakonstantis S., Gikas A., Astrinaki E., Kritsotakis E.I. (2020). Excess mortality due to pandrug-resistant *Acinetobacter baumannii* infections in hospitalized patients. J. Hosp. Infect..

[3] McCreary E.K., Heil E.L., Tamma P.D. (2021). New Perspectives on Antimicrobial Agents: Cefiderocol. Antimicrob. Agents Chemother..

[4] Choby J.E., Ozturk T., Satola S.W., Jacob J.T., Weiss D.S. (2021). Widespread cefiderocol heteroresistance in carbapenem-resistant Gram-negative pathogens. Lancet Infect. Dis..

[5] Karakonstantis S., Saridakis I. (2020). Colistin heteroresistance in *Acinetobacter* spp.: Systematic review and meta-analysis of the prevalence and discussion of the mechanisms and potential therapeutic implications. Int. J. Antimicrob. Agents.

[6] Karakonstantis S., Kritsotakis E.I., Gikas A. (2020). Treatment options for K. pneumoniae, P. aeruginosa and *A. baumannii* co-resistant to carbapenems, aminoglycosides, polymyxins and tigecycline: An approach based on the mechanisms of resistance to carbapenems. Infection.

[7] Wang J., Niu H., Wang R., Cai Y. (2019). Safety and efficacy of colistin alone or in combination in adults with *Acinetobacter baumannii* infection: A systematic review and meta-analysis. Int. J. Antimicrob. Agents.

[8] Salameh M., Daher L.M.A., Chartouny M., Hanna P.A. (2018). Colistin monotherapy v/s colistin combination therapy for treatment of *Acinetobacter* infections, a systematic review. J. Infect. Dev. Ctries..

[9] Paul M., Daikos G.L., Durante-Mangoni E., Yahav D., Carmeli Y., Benattar Y.D., Skiada A., Andini R., Eliakim-Raz N., Nutman A. (2018). Colistin alone versus colistin plus meropenem for treatment of severe infections caused by carbapenem-resistant Gram-negative bacteria: An open-label, randomised controlled trial. Lancet Infect. Dis..

[10] Dickstein Y., Lellouche J., Amar M.B.D., Schwartz D., Nutman A., Daitch V., Yahav D., Leibovici L., Skiada A., Antoniadou A. (2018). Treatment Outcomes of Colistin- and Carbapenem-resistant *Acinetobacter baumannii* Infections: An Exploratory Subgroup Analysis of a Randomized Clinical Trial. Clin. Infect. Dis..

[11] Poulikakos P., Tansarli G.S., Falagas M.E. (2014). Combination antibiotic treatment versus monotherapy for multidrug-resistant, extensively drug-resistant, and pandrug-resistant *Acinetobacter* infections: A systematic review. Eur. J. Clin. Microbiol. Infect. Dis..

[12] Bae S., Kim M.-C., Park S.-J., Kim H.S., Sung H., Kim S.-H., Lee S.-O., Choi S.-H., Woo J.H., Kim Y.S. (2016). In Vitro Synergistic Activity of Antimicrobial Agents in Combination against Clinical Isolates of Colistin-Resistant *Acinetobacter baumannii*. Antimicrob. Agents Chemother..

[13] Karakonstantis S. (2020). Re: ‘Colistin plus meropenem for carbapenem-resistant Gram-negative infections: In vitro synergism is not associated with better clinical outcomes’ by Nutman et al. Clin. Microbiol. Infect..

[14] Mohammadi M., Khayat H., Sayehmiri K., Soroush S., Sayehmiri F., Delfani S., Bogdanovic L., Taherikalani M. (2017). Synergistic Effect of Colistin and Rifampin Against Multidrug Resistant *Acinetobacter baumannii*: A Systematic Review and Meta-Analysis. Open Microbiol. J..

[15] Ni W., Shao X., Di X., Cui J., Wang R., Liu Y. (2015). In vitro synergy of polymyxins with other antibiotics for *Acinetobacter baumannii*: A systematic review and meta-analysis. Int. J. Antimicrob. Agents.

[16] Scudeller L., Righi E., Chiamenti M., Bragantini D., Menchinelli G., Cattaneo P., Giske C.G., Lodise T., Sanguinetti M., Piddock L.J. (2021). Systematic review and meta-analysis of in vitro efficacy of antibiotic combination therapy against carbapenem-resistant Gram-negative bacilli. Int. J. Antimicrob. Agents.

[17] Jiang Z., He X., Li J. (2018). Synergy effect of meropenem-based combinations against *Acinetobacter baumannii*: A systematic review and meta-analysis. Infect. Drug Resist..

[18] Li J., Yang X., Chen L., Duan X., Jiang Z. (2017). In Vitro Activity of Various Antibiotics in Combination with Tigecycline Against *Acinetobacter baumannii*: A Systematic Review and Meta-Analysis. Microb. Drug Resist..

[19] Brennan-Krohn T., Kirby J.E. (2019). When One Drug Is Not Enough: Context, Methodology, and Future Prospects in Antibacterial Synergy Testing. Clin. Lab. Med..

[20] Pillai S.K., Moellering R.C., Eliopoulos G.M., Lorian V. (2005). Antimicrobial combinations. Antibiotics in Laboratory Medicine.

[21] Morrison A., Polisena J., Husereau D., Moulton K., Clark M., Fiander M., Mierzwinski-Urban M., Clifford T., Hutton B., Rabb D. (2012). The effect of English-language restriction on systematic review-based meta-analyses: A systematic review of empirical studies. Int. J. Technol. Assess. Health Care.

[22] Higgins J.P.T., Thomas J., Chandler J., Cumpston M., Li T., Page M.J., Welch V.A.E. Cochrane Handbook for Systematic Reviews of Interventions Version 6.2 (updated February 2021). www.training.cochrane.org/handbook.

[23] Balk E.M., Chung M., Chen M.L., Chang L.K.W., Trikalinos T.A. (2013). Data extraction from machine-translated versus original language randomized trial reports: A comparative study. Syst. Rev..

[24] Ouzzani M., Hammady H., Fedorowicz Z., Elmagarmid A. (2016). Rayyan—A web and mobile app for systematic reviews. Syst. Rev..

[25] Bonapace C.R., Bosso J.A., Friedrich L.V., White R.L. (2002). Comparison of methods of interpretation of checkerboard synergy testing. Diagn. Microbiol. Infect. Dis..

[26] Brill M., Kristoffersson A., Zhao C., Nielsen E., Friberg L. (2018). Semi-mechanistic pharmacokinetic–pharmacodynamic modelling of antibiotic drug combinations. Clin. Microbiol. Infect..

[27] Doern C.D. (2014). When Does 2 Plus 2 Equal 5? A Review of Antimicrobial Synergy Testing. J. Clin. Microbiol..

[28] CLSI (2021). Performance Standards for Antimicrobial Susceptibility Testing.

[29] The European Committee on Antimicrobial Susceptibility Testing Breakpoint Tables for Interpretation of MICs and Zone Diameters. Version 11.0. http://www.eucast.org/.

[30] Sader H.S., Carvalhaes C., Streit J.M., Castanheira M., Flamm R.K. (2020). Antimicrobial activity of cefoperazone-sulbactam tested against Gram-Negative organisms from Europe, Asia-Pacific, and Latin America. Int. J. Infect. Dis..

[31] FDA Plazomicin Infection. FDA-Identified Interpretive Criteria. https://www.fda.gov/drugs/development-resources/plazomicin-injection.

[32] Lepe J.A., García-Cabrera E., Gil-Navarro M.V., Aznar J. (2012). Rifampin breakpoint for *Acinetobacter baumannii* based on pharmacokinetic-pharmacodynamic models with Monte Carlo simulation. Rev. Esp. Quimioter. Publ. Soc. Esp. Quimioter..

[33] Food and Drug Administration (FDA) Tigecycline–Injection Products 2019. https://www.fda.gov/drugs/development-resources/tigecycline-injection-products.

[34] Gordon N., Png K., Wareham D.W. (2010). Potent Synergy and Sustained Bactericidal Activity of a Vancomycin-Colistin Combination versus Multidrug-Resistant Strains of *Acinetobacter baumannii*. Antimicrob. Agents Chemother..

[35] O’Hara J.A., Ambe L.A., Casella L.G., Townsend B.M., Pelletier M.R., Ernst R.K., Shanks R.M.Q., Doi Y. (2013). Activities of Vancomycin-Containing Regimens against Colistin-Resistant *Acinetobacter baumannii* Clinical Strains. Antimicrob. Agents Chemother..

[36] Bowler S.L., Spychala C.N., McElheny C.L., Mettus R.T., Doi Y. (2016). In Vitro Activity of Fusidic Acid-Containing Combinations against Carbapenem-Resistant *Acinetobacter baumannii* Clinical Strains. Antimicrob. Agents Chemother..

[37] Lim S.M.S., Heffernan A.J., Roberts J.A., Sime F.B. (2021). Semimechanistic Pharmacokinetic/Pharmacodynamic Modeling of Fosfomycin and Sulbactam Combination against Carbapenem-Resistant *Acinetobacter baumannii*. Antimicrob. Agents Chemother..

[38] Cebrero-Cangueiro T., Nordmann P., Carretero-Ledesma M., Pachón J., Pachón-Ibáñez M.E. (2021). Efficacy of dual carbapenem treatment in a murine sepsis model of infection due to carbapenemase-producing *Acinetobacter baumannii*. J. Antimicrob. Chemother..

[39] Cheng J., Yan J., Reyna Z., Slarve M., Lu P., Spellberg B., Luna B. (2021). Synergistic Rifabutin and Colistin Reduce Emergence of Resistance When Treating *Acinetobacter baumannii*. Antimicrob. Agents Chemother..

[40] Terbtothakun P., Voravuthikunchai S., Chusri S. (2021). Evaluation of the Synergistic Antibacterial Effects of Fosfomycin in Combination with Selected Antibiotics against Carbapenem–Resistant *Acinetobacter baumannii*. Pharmaceuticals.

[41] Armengol E., Asunción T., Viñas M., Sierra J.M. (2020). When Combined with Colistin, an Otherwise Ineffective Rifampicin–Linezolid Combination Becomes Active in *Escherichia coli*, *Pseudomonas aeruginosa*, and *Acinetobacter baumannii*. Microorganisms.

[42] Li J., Fu Y., Zhang J., Zhao Y., Fan X., Yu L., Wang Y., Zhang X., Li C. (2020). The efficacy of colistin monotherapy versus combination therapy with other antimicrobials against carbapenem-resistant *Acinetobacter baumannii* ST2 isolates. J. Chemother..

[43] Limsrivanichakorn S., Ngamskulrungroj P., Leelaporn A. (2020). Activity of Antimicrobial Combinations Against Extensively Drug-Resistant *Acinetobacter baumannii* as Determined by Checkerboard Method and E-test. Siriraj Med. J..

[44] Lim S.M.S., Naicker S., Ayfan A., Zowawi H., Roberts J., Sime F. (2020). Non-polymyxin-based combinations as potential alternatives in treatment against carbapenem-resistant *Acinetobacter baumannii* infections. Int. J. Antimicrob. Agents.

[45] Lim S.M.S., Heffernan A.J., Roberts J.A., Sime F.B. (2021). Pharmacodynamic Analysis of Meropenem and Fosfomycin Combination Against Carbapenem-Resistant *Acinetobacter baumannii* in Patients with Normal Renal Clearance: Can It Be a Treatment Option?. Microb. Drug Resist..

[46] Nordmann P., Perler J., Kieffer N., Poirel L. (2020). In-vitro evaluation of a dual carbapenem combination against carbapenemase-producing *Acinetobacter baumannii*. J. Infect..

[47] Rodriguez C.H., Brune A., Nastro M., Vay C., Famiglietti A. (2020). In vitro synergistic activity of the sulbactam/avibactam combination against extensively drug-resistant *Acinetobacter baumannii*. J. Med. Microbiol..

[48] Gaudereto J.J., Neto L.V.P., Leite G.C., Martins R.R., Prado G.V.B.D., Rossi F., Guimarães T., Levin A.S., Costa S.F. (2019). Synergistic Effect of Ceftazidime-Avibactam with Meropenem against Panresistant, Carbapenemase-Harboring *Acinetobacter baumannii* and Serratia marcescens Investigated Using Time-Kill and Disk Approximation Assays. Antimicrob. Agents Chemother..

[49] Ghaith D., Hassan R., Dawoud M.E.E.-D., Eweis M., Metwally R., Zafer M. (2019). Effect of rifampicin–colistin combination against XDR *Acinetobacter baumannii* harbouring blaOXA 23-like gene and showed reduced susceptibility to colistin at Cairo University Hospital, Cairo, Egypt. Infect. Dis..

[50] Kara E.M., Yılmaz M., Çelik B. (2019). In vitro activities of ceftazidime/avibactam alone or in combination with antibiotics against multidrug-resistant *Acinetobacter baumannii* isolates. J. Glob. Antimicrob. Resist..

[51] Menegucci T.C., Fedrigo N.H., Lodi F.G., Albiero J., Nishiyama S.A.B., Mazucheli J., Carrara-Marroni F.E., Voelkner N.M.F., Gong H., Sy S. (2019). Pharmacodynamic Effects of Sulbactam/Meropenem/Polymyxin-B Combination Against Extremely Drug Resistant *Acinetobacter baumannii* Using Checkerboard Information. Microb. Drug Resist..

[52] Oliva A., Garzoli S., De Angelis M., Marzuillo C., Vullo V., Mastroianni C.M., Ragno R. (2019). In-Vitro Evaluation of Different Antimicrobial Combinations with and without Colistin Against Carbapenem-Resistant *Acinetobacter baumannii*. Molecules.

[53] Ozger H.S., Cuhadar T., Yildiz S.S., Gulmez Z.D., Dizbay M., Tunccan O.G., Kalkanci A., Simsek H., Unaldi O. (2019). In vitro activity of eravacycline in combination with colistin against carbapenem-resistant *A. baumannii* isolates. J. Antibiot..

[54] Phee L.M., Kloprogge F., Morris R., Barrett J., Wareham D.W., Standing J.F. (2019). Pharmacokinetic-pharmacodynamic modelling to investigate in vitro synergy between colistin and fusidic acid against MDR *Acinetobacter baumannii*. J. Antimicrob. Chemother..

[55] Poulakou G., Renieris G., Sabrakos L., Zarkotou O., Themeli-Digalaki K., Perivolioti E., Kraniotaki E., Giamarellos-Bourboulis E.J., Zavras N. (2018). Daptomycin as adjunctive treatment for experimental infection by *Acinetobacter baumannii* with resistance to colistin. Int. J. Antimicrob. Agents.

[56] Shinohara D.R., Menegucci T.C., Fedrigo N.H., Migliorini L.B., Carrara-Marroni F.E., Anjos M., Cardoso C.L., Nishiyama S.A.B., Tognim M.C.B. (2019). Synergistic activity of polymyxin B combined with vancomycin against carbapenem-resistant and polymyxin-resistant *Acinetobacter baumannii*: First in vitro study. J. Med. Microbiol..

[57] Wang J., Ning Y., Li S., Wang Y., Liang J., Jin C., Yan H., Huang Y. (2019). Multidrug-resistant *Acinetobacter baumannii* strains with NDM-1: Molecular characterization and in vitro efficacy of meropenem-based combinations. Exp. Ther. Med..

[58] Chen F., Wang L., Wang M., Xie Y., Xia X., Li X., Liu Y., Cao W., Zhang T., Li P. (2018). Genetic characterization and in vitro activity of antimicrobial combinations of multidrug-resistant *Acinetobacter baumannii* from a general hospital in China. Oncol. Lett..

[59] Singkham-In U., Chatsuwan T. (2018). In vitro activities of carbapenems in combination with amikacin, colistin, or fosfomycin against carbapenem-resistant *Acinetobacter baumannii* clinical isolates. Diagn. Microbiol. Infect. Dis..

[60] Zhu W., Wang Y., Cao W., Cao S., Zhang J. (2018). In vitro evaluation of antimicrobial combinations against imipenem-resistant *Acinetobacter baumannii* of different MICs. J. Infect. Public Health.

[61] Lenhard J.R., Thamlikitkul V., Silveira F.P., Garonzik S.M., Tao X., Forrest A., Shin B.S., Kaye K.S., Bulitta J.B., Nation R.L. (2017). Polymyxin-resistant, carbapenem-resistant *Acinetobacter baumannii* is eradicated by a triple combination of agents that lack individual activity. J. Antimicrob. Chemother..

[62] Lenhard J., Smith N.M., Bulman Z.P., Tao X., Thamlikitkul V., Shin B.S., Nation R.L., Li J., Bulitta J.B., Tsuji B.T. (2017). High-Dose Ampicillin-Sulbactam Combinations Combat Polymyxin-Resistant *Acinetobacter baumannii* in a Hollow-Fiber Infection Model. Antimicrob. Agents Chemother..

[63] Madadi-Goli N., Moniri R., Bagheri-Josheghani S., Dasteh-Goli N. (2017). Sensitivity of levofloxacin in combination with ampicillin-sulbactam and tigecycline against multidrug-resistant *Acinetobacter baumannii*. Iran. J. Microbiol..

[64] Wei W., Yang H., Hu L., Ye Y., Li J. (2017). Activity of levofloxacin in combination with colistin against *Acinetobacter baumannii*: In vitro and in a Galleria mellonella model. J. Microbiol. Immunol. Infect..

[65] Wei W.-J., Yang H.-F. (2017). Synergy against extensively drug-resistant *Acinetobacter baumannii* in vitro by two old antibiotics: Colistin and chloramphenicol. Int. J. Antimicrob. Agents.

[66] Hong D.J., Kim J.O., Lee H., Yoon E.-J., Jeong S.H., Yong D., Lee K. (2016). In vitro antimicrobial synergy of colistin with rifampicin and carbapenems against colistin-resistant *Acinetobacter baumannii* clinical isolates. Diagn. Microbiol. Infect. Dis..

[67] Laishram S., Anandan S., Devi B.Y., Elakkiya M., Priyanka B., Bhuvaneshwari T., Peter J.V., Subramani K., Balaji V. (2016). Determination of synergy between sulbactam, meropenem and colistin in carbapenem-resistant *Klebsiella pneumoniae* and *Acinetobacter baumannii* isolates and correlation with the molecular mechanism of resistance. J. Chemother..

[68] Leite G.C., Oliveira M.S., Perdigão-Neto L.V., Rocha C.K.D., Guimarães T., Rizek C., Levin A., Costa S.F. (2016). Antimicrobial Combinations against Pan-Resistant *Acinetobacter baumannii* Isolates with Different Resistance Mechanisms. PLoS ONE.

[69] Menegucci T.C., Albiero J., Migliorini L.B., Alves J.L.B., Viana G.F., Mazucheli J., Carrara-Marroni F.E., Cardoso C.L., Tognim M.C.B. (2016). Strategies for the treatment of polymyxin B-resistant *Acinetobacter baumannii* infections. Int. J. Antimicrob. Agents.

[70] Yang H., Lv N., Hu L., Liu Y., Cheng J., Ye Y., Li J. (2016). In vivoactivity of vancomycin combined with colistin against multidrug-resistant strains of *Acinetobacter baumannii* in aGalleriamellonellamodel. Infect. Dis..

[71] Yang Y.-S., Lee Y., Tseng K.-C., Huang W.-C., Chuang M.-F., Kuo S.-C., Lauderdale T.-L.Y., Chen T.-L. (2016). In Vivo and In Vitro Efficacy of Minocycline-Based Combination Therapy for Minocycline-Resistant *Acinetobacter baumannii*. Antimicrob. Agents Chemother..

[72] Yavaş S., Yetkin M.A., Kayaaslan B., Baştuğ A., Aslaner H., But A., Kanyilmaz D., Sari B., Akinci E., Bodur H. (2016). Investigating the in vitro synergistic activities of several antibiotic combinationsagainst carbapenem-resistant *Acinetobacter baumannii* isolates. Turk. J. Med. Sci..

[73] Córdoba J., Coronado-Álvarez N.M., Parra D., Parra-Ruiz J. (2015). In Vitro Activities of Novel Antimicrobial Combinations against Extensively Drug-Resistant *Acinetobacter baumannii*. Antimicrob. Agents Chemother..

[74] García-Salguero C., Rodríguez-Avial I., Picazo J.J., Culebras E. (2015). Can Plazomicin Alone or in Combination Be a Therapeutic Option against Carbapenem-Resistant *Acinetobacter baumannii*?. Antimicrob. Agents Chemother..

[75] Marie M.A.M., Krishnappa L.G., Alzahrani A.J., Mubaraki M.A., Alyousef A.A. (2015). A prospective evaluation of synergistic effect of sulbactam and tazobactam combination with meropenem or colistin against multidrug resistant *Acinetobacter baumannii*. Bosn. J. Basic Med. Sci..

[76] Phee L.M., Betts J., Bharathan B., Wareham D.W. (2015). Colistin and Fusidic Acid, a Novel Potent Synergistic Combination for Treatment of Multidrug-Resistant *Acinetobacter baumannii* Infections. Antimicrob. Agents Chemother..

[77] Rodríguez C.H., Nastro M., Vay C., Famiglietti A. (2015). In vitro activity of minocycline alone or in combination in multidrug-resistant *Acinetobacter baumannii* isolates. J. Med. Microbiol..

[78] Vourli S., Frantzeskaki F., Meletiadis J., Stournara L., Armaganidis A., Zerva L., Dimopoulos G. (2015). Synergistic interactions between colistin and meropenem against extensively drug-resistant and pandrug-resistant *Acinetobacter baumannii* isolated from ICU patients. Int. J. Antimicrob. Agents.

[79] Galani I., Orlandou K., Moraitou H., Petrikkos G., Souli M. (2014). Colistin/daptomycin: An unconventional antimicrobial combination synergistic in vitro against multidrug-resistant *Acinetobacter baumannii*. Int. J. Antimicrob. Agents.

[80] Majewski P., Wieczorek P., Ojdana D., Sacha P., Wieczorek A., Tryniszewska E. (2014). In vitro activity of rifampicin alone and in combination with imipenem against multidrug-resistant *Acinetobacter baumannii* harboring theblaOXA-72resistance gene. Scand. J. Infect. Dis..

[81] Nastro M., Rodríguez C.H., Monge R., Zintgraff J., Neira L., Rebollo M., Vay C., Famiglietti A. (2014). Activity of the colistin–rifampicin combination against colistin-resistant, carbapenemase-producing Gram-negative bacteria. J. Chemother..

[82] Oleksiuk L.M., Nguyen M.H., Press E.G., Updike C.L., O’Hara J.A., Doi Y., Clancy C.J., Shields R.K. (2014). In VitroResponses of *Acinetobacter baumannii* to Two- and Three-Drug Combinations following Exposure to Colistin and Doripenem. Antimicrob. Agents Chemother..

[83] Percin D., Akyol S., Kalin G. (2014). In vitro synergism of combinations of colistin with selected antibiotics against colistin-resistant *Acinetobacter baumannii*. GMS Hyg. Infect. Control.

[84] Sun Y., Wang L., Li J., Zhao C., Zhao J., Liu M., Wang S., Lu C., Shang G., Jia Y. (2014). Synergistic efficacy of meropenem and rifampicin in a murine model of sepsis caused by multidrug-resistant *Acinetobacter baumannii*. Eur. J. Pharmacol..

[85] Wang Y., Bao W., Guo N., Chen H., Cheng W., Jin K., Shen F., Xu J., Zhang Q., Wang C. (2014). Antimicrobial activity of the imipenem/rifampicin combination against clinical isolates of *Acinetobacter baumannii* grown in planktonic and biofilm cultures. World J. Microbiol. Biotechnol..

[86] Cetin E.S., Tekeli A., Ozseven A.G., Us E., Aridogan B.C. (2013). Determination of In Vitro Activities of Polymyxin B and Rifampin in Combination with Ampicillin/Sulbactam or Cefoperazone/Sulbactam against Multidrug-Resistant *Acinetobacter baumannii* by the E-test and Checkerboard Methods. Jpn. J. Infect. Dis..

[87] Housman S.T., Hagihara M., Nicolau D.P., Kuti J.L. (2013). In vitro pharmacodynamics of human-simulated exposures of ampicillin/sulbactam, doripenem and tigecycline alone and in combination against multidrug-resistant *Acinetobacter baumannii*. J. Antimicrob. Chemother..

[88] Lee H.J., Bergen P.J., Bulitta J., Tsuji B., Forrest A., Nation R.L., Li J. (2013). Synergistic Activity of Colistin and Rifampin Combination against Multidrug-Resistant *Acinetobacter baumannii* in anIn VitroPharmacokinetic/Pharmacodynamic Model. Antimicrob. Agents Chemother..

[89] Principe L., Capone A., Mazzarelli A., D’Arezzo S., Bordi E., Di Caro A., Petrosillo N. (2013). In Vitro Activity of Doripenem in Combination with Various Antimicrobials Against Multidrug-Resistant *Acinetobacter baumannii*: Possible Options for the Treatment of Complicated Infection. Microb. Drug Resist..

[90] Queenan A.M., A Davies T., He W., Lynch A.S. (2013). Assessment of the combination of doripenem plus a fluoroquinolone against non-susceptible *Acinetobacter baumannii* isolates from nosocomial pneumonia patients. J. Chemother..

[91] Deveci A., Coban A.Y., Acicbe O., Tanyel E., Yaman G., Durupinar B. (2012). In vitro effects of sulbactam combinations with different antibiotic groups against clinical *Acinetobacter baumannii* isolates. J. Chemother..

[92] Peck K.R., Kim M.J., Choi J.Y., Kim H.S., Kang C.-I., Cho Y.K., Park D.W., Lee H.J., Lee M.S., Ko K.S. (2012). In vitro time-kill studies of antimicrobial agents against blood isolates of imipenem-resistant *Acinetobacter baumannii*, including colistin- or tigecycline-resistant isolates. J. Med. Microbiol..

[93] Vidaillac C., Benichou L., Duval R. (2012). In VitroSynergy of Colistin Combinations against Colistin-Resistant *Acinetobacter baumannii*, *Pseudomonas aeruginosa*, and *Klebsiella pneumoniae* Isolates. Antimicrob. Agents Chemother..

[94] Pachón-Ibáñez M.E., Docobo-Pérez F., Jiménez-Mejías M.E., Ibáñez-Martínez J., García-Curiel A., Pichardo C., Pachón J. (2011). Efficacy of rifampin, in monotherapy and in combinations, in an experimental murine pneumonia model caused by panresistant *Acinetobacter baumannii* strains. Eur. J. Clin. Microbiol. Infect. Dis..

[95] Santimaleeworagun W., Wongpoowarak P., Chayakul P., Pattharachayakul S., Tansakul P., Garey K.W. (2011). In vitro activity of colistin or sulbactam in combination with fosfomycin or imipenem against clinical isolates of carbapenem-resistant *Acinetobacter baumannii* producing OXA-23 carbapenemases. Southeast Asian J. Trop. Med. Public Health.

[96] Tan T.Y., Lim T.P., Lee W.H.L., Sasikala S., Hsu L.Y., Kwa A.L.-H. (2011). In VitroAntibiotic Synergy in Extensively Drug-Resistant *Acinetobacter baumannii*: The Effect of Testing by Time-Kill, Checkerboard, and Etest Methods. Antimicrob. Agents Chemother..

[97] Kiratisin P., Apisarnthanarak A., Kaewdaeng S. (2010). Synergistic activities between carbapenems and other antimicrobial agents against *Acinetobacter baumannii* including multidrug-resistant and extensively drug-resistant isolates. Int. J. Antimicrob. Agents.

[98] Pachón-Ibáñez M.E., Docobo-Pérez F., López-Rojas R., Domínguez-Herrera J., Jiménez-Mejías M.E., García-Curiel A., Pichardo C., Jiménez L., Pachón J. (2010). Efficacy of Rifampin and Its Combinations with Imipenem, Sulbactam, and Colistin in Experimental Models of Infection Caused by Imipenem-Resistant *Acinetobacter baumannii*. Antimicrob. Agents Chemother..

[99] Pankuch G.A., Seifert H., Appelbaum P.C. (2010). Activity of doripenem with and without levofloxacin, amikacin, and colistin against *Pseudomonas aeruginosa* and *Acinetobacter baumannii*. Diagn. Microbiol. Infect. Dis..

[100] Rodriguez C.H., De Ambrosio A., Bajuk M., Spinozzi M., Nastro M., Bombicino K., Radice M., Gutkind G., Vay C., Famiglietti A. (2010). In vitro antimicrobials activity against endemic *Acinetobacter baumannii* multiresistant clones. J. Infect. Dev. Ctries..

[101] Urban C., Mariano N., Rahal J.J. (2010). In Vitro Double and Triple Bactericidal Activities of Doripenem, Polymyxin B, and Rifampin against Multidrug-Resistant *Acinetobacter baumannii*, *Pseudomonas aeruginosa*, *Klebsiella pneumoniae*, and *Escherichia coli*. Antimicrob. Agents Chemother..

[102] Yuan Z., Ledesma K.R., Singh R., Hou J., Prince R.A., Tam V.H. (2010). Quantitative Assessment of Combination Antimicrobial Therapy against Multidrug-Resistant Bacteria in a Murine Pneumonia Model. J. Infect. Dis..

[103] Lim T.-P., Tan T.-Y., Lee W., Sasikala S., Tan T.-T., Hsu L.-Y., Kwa A.L. (2009). In vitro activity of various combinations of antimicrobials against carbapenem-resistant *Acinetobacter* species in Singapore. J. Antibiot..

[104] Principe L., D’Arezzo S., Capone A., Petrosillo N., Visca P. (2009). In vitro activity of tigecycline in combination with various antimicrobials against multidrug resistant *Acinetobacter baumannii*. Ann. Clin. Microbiol. Antimicrob..

[105] Song J.Y., Cheong H.J., Lee J., Sung A.K., Kim W.J. (2009). Efficacy of monotherapy and combined antibiotic therapy for carbapenem-resistant *Acinetobacter baumannii* pneumonia in an immunosuppressed mouse model. Int. J. Antimicrob. Agents.

[106] Lee C.-H., Tang Y.-F., Su L.-H., Chien C.-C., Liu J.-W. (2008). Antimicrobial Effects of Varied Combinations of Meropenem, Sulbactam, and Colistin on a Multidrug-Resistant *Acinetobacter baumannii* Isolate That Caused Meningitis and Bacteremia. Microb. Drug Resist..

[107] Lim T.-P., Ledesma K.R., Chang K.-T., Hou J.-G., Kwa A.L., Nikolaou M., Quinn J.P., Prince R.A., Tam V.H. (2008). Quantitative Assessment of Combination Antimicrobial Therapy against Multidrug-Resistant *Acinetobacter baumannii*. Antimicrob. Agents Chemother..

[108] Lee N.-Y., Wang C.-L., Chuang Y.-C., Yu W.-L., Lee H.-C., Chang C.-M., Wang L.-R., Ko W.-C. (2007). Combination Carbapenem-Sulbactam Therapy for Critically Ill Patients with Multidrug-Resistant *Acinetobacter baumannii* Bacteremia: Four Case Reports and an In Vitro Combination Synergy Study. Pharmacother. J. Hum. Pharmacol. Drug Ther..

[109] Sader H.S., Rhomberg P., Jones R.N. (2005). In Vitro Activity of β-Lactam Antimicrobial Agents in Combination with Aztreonam Tested Against Metallob-β-Lactamase-Producing *Pseudomonas aeruginosa* and *Acinetobacter baumannii*. J. Chemother..

[110] Sader H.S., Jones R.N. (2005). Comprehensive in vitro evaluation of cefepime combined with aztreonam or ampicillin/sulbactam against multi-drug resistant *Pseudomonas aeruginosa* and *Acinetobacter* spp. Int. J. Antimicrob. Agents.

[111] Choi J.Y., Park Y.S., Cho C.H., Shin S.Y., Song Y.G., Yong D., Lee K., Kim J.M. (2004). Synergic in-vitro activity of imipenem and sulbactam against *Acinetobacter baumannii*. Clin. Microbiol. Infect..

[112] Jung R., Husain M., Choi M.K., Fish D.N. (2004). Synergistic Activities of Moxifloxacin Combined with Piperacillin-Tazobactam or Cefepime against *Klebsiella pneumoniae*, Enterobacter cloacae, and *Acinetobacter baumannii* Clinical Isolates. Antimicrob. Agents Chemother..

[113] Montero A., Ariza J., Corbella X., Doménech A., Cabellos C., Ayats J., Tubau F., Borraz C., Gudiol F. (2004). Antibiotic combinations for serious infections caused by carbapenem-resistant *Acinetobacter baumannii* in a mouse pneumonia model. J. Antimicrob. Chemother..

[114] Yoon J., Urban C., Terzian C., Mariano N., Rahal J.J. (2004). In Vitro Double and Triple Synergistic Activities of Polymyxin B, Imipenem, and Rifampin against Multidrug-Resistant *Acinetobacter baumannii*. Antimicrob. Agents Chemother..

[115] Fernández-Cuenca F., Martínez L.M., Pascual A., Perea E.J. (2003). In vitro Activity of Azithromycin in Combination with Amikacin, Ceftazidime, Ciprofloxacin or Imipenem against Clinical Isolates of *Acinetobacter baumannii*. Chemotherapy.

[116] Roussel-Delvallez M., Wallet F., Delpierre F., Courcol R. (1996). In Vitro Bactericidal Effect of a β-lactam + Aminoglycoside Combination Against Multiresistant *Pseudomonas aeruginosa* and *Acinetobacter baumannii*. J. Chemother..

[117] Park G.C., Choi J.A., Jang S.J., Jeong S.H., Kim C.-M., Choi I.S., Kang S.H., Park G., Moon D.S. (2016). In Vitro Interactions of Antibiotic Combinations of Colistin, Tigecycline, and Doripenem Against Extensively Drug-Resistant and Multidrug-Resistant *Acinetobacter baumannii*. Ann. Lab. Med..

[118] Lim S.M.S., Heffernan A.J., Zowawi H.M., Roberts J.A., Sime F.B. (2021). Semi-mechanistic PK/PD modelling of meropenem and sulbactam combination against carbapenem-resistant strains of *Acinetobacter baumannii*. Eur. J. Clin. Microbiol. Infect. Dis..

[119] Jaruratanasirikul S., Wongpoowarak W., Wattanavijitkul T., Sukarnjanaset W., Samaeng M., Nawakitrangsan M., Ingviya N. (2016). Population Pharmacokinetics and Pharmacodynamics Modeling to Optimize Dosage Regimens of Sulbactam in Critically Ill Patients with Severe Sepsis Caused by *Acinetobacter baumannii*. Antimicrob. Agents Chemother..

[120] Jaruratanasirikul S., Nitchot W., Wongpoowarak W., Samaeng M., Nawakitrangsan M. (2019). Population pharmacokinetics and Monte Carlo simulations of sulbactam to optimize dosage regimens in patients with ventilator-associated pneumonia caused by *Acinetobacter baumannii*. Eur. J. Pharm. Sci..

[121] Asuphon O., Montakantikul P., Houngsaitong J., Kiratisin P., Sonthisombat P. (2016). Optimizing intravenous fosfomycin dosing in combination with carbapenems for treatment of *Pseudomonas aeruginosa* infections in critically ill patients based on pharmacokinetic/pharmacodynamic (PK/PD) simulation. Int. J. Infect. Dis..

[122] Pasteran F., Cedano J., Baez M., Albornoz E., Rapoport M., Osteria J., Montaña S., Le C., Ra G., Bonomo R. (2021). A New Twist: The Combination of Sulbactam/Avibactam Enhances Sulbactam Activity against Carbapenem-Resistant *Acinetobacter baumannii* (CRAB) Isolates. Antibiotics.

[123] Savoldi A., Carrara E., Piddock L.J.V., Franceschi F., Ellis S., Chiamenti M., Bragantini D., Righi E., Tacconelli E. (2021). The role of combination therapy in the treatment of severe infections caused by carbapenem resistant gram-negatives: A systematic review of clinical studies. BMC Infect. Dis..

[124] Kofteridis D.P., Andrianaki A.M., Maraki S., Mathioudaki A., Plataki M., Alexopoulou C., Ioannou P., Samonis G., Valachis A. (2020). Treatment pattern, prognostic factors, and outcome in patients with infection due to pan-drug-resistant gram-negative bacteria. Eur. J. Clin. Microbiol. Infect. Dis..

[125] Assimakopoulos S.F., Karamouzos V., Lefkaditi A., Sklavou C., Kolonitsiou F., Christofidou M., Fligou F., Gogos C., Marangos M. (2019). Triple combination therapy with high-dose ampicillin/sulbactam, high-dose tigecycline and colistin in the treatment of ventilator-associated pneumonia caused by pan-drug resistant *Acinetobacter baumannii*: A case series study. Infez. Med..

[126] Qureshi Z.A., Hittle L.E., O’Hara J.A., Rivera J.I., Syed A., Shields R.K., Pasculle A.W., Ernst R., Doi Y. (2015). Colistin-Resistant *Acinetobacter baumannii*: Beyond Carbapenem Resistance. Clin. Infect. Dis..

[127] Park H.J., Cho J.H., Kim H.J., Han S.H., Jeong S.H., Byun M.K. (2019). Colistin monotherapy versus colistin/rifampicin combination therapy in pneumonia caused by colistin-resistant *Acinetobacter baumannii*: A randomised controlled trial. J. Glob. Antimicrob. Resist..

[128] Hernan R.C., Karina B., Gabriela G., Marcela N., Carlos V., Angela F. (2009). Selection of colistin-resistant *Acinetobacter baumannii* isolates in postneurosurgical meningitis in an intensive care unit with high presence of heteroresistance to colistin. Diagn. Microbiol. Infect. Dis..

[129] Cai Y., Chai D., Wang R., Liang B., Bai N. (2012). Colistin resistance of *Acinetobacter baumannii*: Clinical reports, mechanisms and antimicrobial strategies. J. Antimicrob. Chemother..

[130] Luna B., Trebosc V., Lee B., Bakowski M., Ulhaq A., Yan J., Lu P., Cheng J., Nielsen T., Lim J. (2020). A nutrient-limited screen unmasks rifabutin hyperactivity for extensively drug-resistant *Acinetobacter baumannii*. Nat. Microbiol..

[131] Trebosc V., Schellhorn B., Schill J., Lucchini V., Bühler J., Bourotte M., Butcher J.J., Gitzinger M., Lociuro S., Kemmer C. (2020). In vitro activity of rifabutin against 293 contemporary carbapenem-resistant *Acinetobacter baumannii* clinical isolates and characterization of rifabutin mode of action and resistance mechanisms. J. Antimicrob. Chemother..

[132] Fan B., Guan J., Wang X., Cong Y. (2016). Activity of Colistin in Combination with Meropenem, Tigecycline, Fosfomycin, Fusidic Acid, Rifampin or Sulbactam against Extensively Drug-Resistant *Acinetobacter baumannii* in a Murine Thigh-Infection Model. PLoS ONE.

[133] Elsayed E., Elarabi M.A., Sherif D.A., Elmorshedi M., El-Mashad N. (2020). Extensive drug resistant *Acinetobacter baumannii*: A comparative study between non-colistin based combinations. Int. J. Clin. Pharm..

[134] Xie J., Roberts J.A., Alobaid A.S., Roger C., Wang Y., Yang Q., Sun J., Dong H., Wang X., Xing J. (2017). Population Pharmacokinetics of Tigecycline in Critically Ill Patients with Severe Infections. Antimicrob. Agents Chemother..

[135] Song X., Wu Y., Cao L., Yao D., Long M. (2019). Is Meropenem as a Monotherapy Truly Incompetent for Meropenem-Nonsusceptible Bacterial Strains? A Pharmacokinetic/Pharmacodynamic Modeling With Monte Carlo Simulation. Front. Microbiol..

[136] Van Wart S.A., Andes D., Ambrose P.G., Bhavnani S.M. (2009). Pharmacokinetic–pharmacodynamic modeling to support doripenem dose regimen optimization for critically ill patients. Diagn. Microbiol. Infect. Dis..

