# Supplementary material for: Systematic Review of Antimicrobial Combination Options for Pandrug-Resistant Acinetobacter baumannii
Source: Antibiotics (Basel). 2021 Nov 3;10(11):1344. doi: 10.3390/antibiotics10111344 (PMC8615225; doi:10.3390/antibiotics10111344)
Supplement: Supplementary file 1 [file antibiotics-10-01344-s001.zip › antibiotics-1442471-supplementary.pdf]

Supplementary Materials

File S1: Table of Contents

1 Abbreviations 3

2 Summary of reviewed studies 5

2.1 Summary and characteristics of reviewed studies 5

2.2 Distribution of studies by year of publication 12

2.3 Distribution of studies by country and WHO regions 13

2.4 Number of eligible isolates per study 14

2.5 Number of studies with single-centre vs multicentre design 14

3 Overview of number of studies and eligible isolate for each combinations evaluated 15

3.1 Summary table; Number of studies and eligible isolates per combination and method used 15

3.2 Few studies available for each combination 28

3.3 Number of studies per combination (only double combinations shown) 29

3.4 Number of eligible isolates per combination (only double combinations shown) 31

3.5 Number of studies per combinations (only double combinations shown) tested by checkerboard method 33

3.6 Number of eligible isolates per combination (only double combinations are shown) tested by checkerboard method 35

3.7 Number of eligible studies (only double combinations shown) tested by TKA 37

3.8 Number of eligible isolates (only double combinations shown) tested by TKA 39

3.9 Number of eligible studies tested by gradient methods 41

3.10 Number of eligible isolates tested by gradient methods 41

3.11 Number of eligible studies tested by disk methods 42

3.12 Number of eligible isolates tested by disk methods 42

3.13 Number of eligible studies tested by agar dilution method 43

3.14 Number of eligible isolates tested by agar dilution method 43

3.15 Number of eligible studies (only double combinations shown) tested by dynamic in vitro PK/PD models 44

3.16 Number of eligible isolates (only double combinations shown) tested by dynamic in vitro PK/PD models ... 44

3.17 Number of eligible studies (only double combinations shown) tested by animal models 46

3.18 Number of eligible isolates (only double combinations shown) tested by animal models ... 46

4 Assessment of clinical relevance of observed synergy ... 48

4.1 Summary Table (sum of all studies): Proportion of synergy and clinically relevant synergy in checkerboard (CHBD) and time-kill assay (TKA) ... 48

4.2 Summary Table (sum of all studies): Proportion of synergy and clinically relevant synergy in gradient- and disk-based methods ... 60

4.3 Summary Table (sum of all studies): Proportion of synergy and clinically relevant synergy in agar dilution and dynamic in vitro PK/PD models ... 64

4.4 Proportion of synergy and assessment of clinical relevance of observed synergy in checkerboard assays (study level data) ... 67

4.5 Proportion of synergy and assessment of clinical relevance of observed synergy in time-kill assays (study level data) ... 79

4.6 Proportion of synergy and assessment of clinical relevance of observed synergy in studies using dynamic in vitro PK/PD models (study level data) ... 88

4.7 Proportion of synergy and assessment of clinical relevance of observed synergy in studies using animal models (study level data) ... 89

4.8 Proportion of synergy and assessment of clinical relevance of observed synergy in other methods (study level data) ... 92

5 Explanations for articles excluded as irrelevant ... 98

6 List of non-English articles excluded ... 99

7 References ... 101

1 Abbreviations

AMK= amikacin,

ATM= aztreonam,

AVI= avibactam

AZM= azithromycin,

CAZ=ceftazidime,

CFS= cefoperazone/sulbactam,

CHBD= checkerboard assay

CHL= chloramphenicol,

CIP= ciprofloxacine,

CRO= ceftriaxone,

CST= colistin,

CZA= ceftazidime/avibactam,

DAP= daptomycin,

DDST= double-disk synergy test,

DOR= doripenem,

ERV= eravacycline,

FA= fusidic acid, **FEP**= cefepime,

FICI= fractional inhibitory concentration index,

FOF= fosfomycin,

GEN= gentamicin,

HFIM= hollow-fiber infection model,

IMP= imipenem,

LVX=levofloxacin,

LZD= linezolid,

MCBT= multiple-combination bactericidal test,

MIN= minocycline,

MEM= meropenem,

MXF= moxifloxacin,

NR= not reported,

PK/PD= pharmacokinetic/pharmacodynamic study,

PLZ= plazomicin,

PMB= polymyxin-B,

RFB= rifabutin,

RIF= rifampicin,

SAM= ampicillin/sulbactam,

SUL= sulbactam,

SXT= trimethoprim/sulfamethoxazole,

TEC= teicoplanin,

TIM= ticarcillin-clavulanic acid,

TGC= tigecycline,

TKA= time-kill assay,

TMP= trimethoprim,

TOB= tobramycin,

TZB= tazobactam,

TZP= piperacillin/tazobactam,

VAN= vancomycin

2 Summary of reviewed studies

2.1 Summary and characteristics of reviewed studies

Author-Year	Country (number of institutions from which the isolates were collected)	Methods for evaluation of synergy	Antimicrobial combinations tested (synergy present/number of eligible[1] strains)
**Cebrero-Cangueiro T, 2021 [1] and Nordmann P, 2020 [2]**	Barhein, Brazil, Colombia, France, Saudi Arabia, Switzerland, and Turkey (Unclear number of institutions)	CHBD, intraperitoneal infection mouse model	**MEM/IMP** (CHBD 6/21, animal model 2/2)
**Cheng J 2021 [3]**	USA (NR)	CHBD	**CST/RIF** (2/2), **CST/RBT** (3/3)
**Nwabor OF 2021 [4]**	Thailand (4)	CHBD, TKA	**FOF/IMP** (CHBD 0/2), **FOF/MEM** (CHBD 0/1), **FOF/DOR** (CHBD 3/3, TKA 0/1), **FOF/GEN** (CHBD 2/2), **FOF/TOB** (CHBD 1/2), **FOF/CIP** (CHBD 1/2, TKA 0/1), **FOF/LVX** (CHBD 0/1), **FOF/TGC** (CHBD 3/4, TKA 1/1)
**Armengol E, 2020 [5]**	Spain (1)	CHBD	**RIF/LZD** (0/3)
**Li J, 2020 [6]**	China (1)	CHBD, TKA	**CST/MEM** (CHBD 4/5, TKA 1/1), **CST/LVX** (CHBD 1/1, TKA 1/1)
**Limsrivanichakorn S, 2020 [7]**	Thailand (1)	CHBD, E-test	**CFS/MXF** (CHBD 4/80, E-test 2/80)
**Mohd Sazlly Lim S 2020 [8], 2021 [9], 2021 [10]**	Saudi Arabia, United Arab Emirates, Oman, Kuwait, Qatar, Bahrain (7)	CHBD, TKA, semi-mechanistic PK/PD, Monte-Carlo simulation	<u>**CHBD**</u>: **FOF/SUL** (37/50), **MEM/SUL** (28/50), **FOF/MEM** (14/50), **FOF/RIF** (12/50), **MEM/RIF** (10/50), **RIF/SUL** (10/50). <u>**TKA**</u>: **FOF/SUL** (3/4) <u>**semi-mechanistic PK/PD**</u>: **FOF/SUL** (based on TKA for 2 isolates synergy was achievable at clinically relevant concentration). <u>**PK/PD modelling and Monte-Carlo simulations**</u>: **FOF/MEM** (proposed breakpoints: MEM 8 mg/L, FOF 128 mg/L)
**Rodriguez CH, 2020 [11]**	South America (6 countries, 15 hospitals)	Agar dilution, TKA	**SUL/AVI** (agar dilution 35/38, TKA 1/1)
**Gaudereto JJ, 2019**	Brazil (1)	DDST, TKA	**CZA/MEM** (DDST 2/11,

Author-Year	Country (number of institutions from which the isolates were collected)	Methods for evaluation of synergy	Antimicrobial combinations tested (synergy present/number of eligible[1] strains)
**[12]**			TKA 0/11)
**Ghaith D, 2019 [13]**	Egypt (NR)	CHBD	**CST/RIF** (14/23)
**Mataraci Kara E, 2019 [14]**	Turkey (1)	TKA	**CST/CZA** (2/2), **CZA/LVX** (3/4), **CZA/TGC** (3/4), **CZA/TOB** (1/2), **CZA/MEM** (3/4)
**Mengucci TC 2019 [15], 2016 [16]**	Brazil (2)	CHBD	**PMB/MEM** (3/3), **PMB/SUL** (2/3), **PMB/FOF** (0/3), **MEM/FOF** (1/6), **MEM/SUL** (6), **PMB/MEM/FOF** (3), **PMB/MEM/SUL** (3/3)
**Oliva A, 2019 [17]**	Italy (1)	CHBD, TKA	**CST/VAN** (CHBD 2/2, TKA 2/2), **CST/RIF** (CHBD 2/2, TKA 2/2), **CST/MEM** (CHBD 1/2, TKA 2/2s)
**Ozger HS, 2019 [18]**	Turkey (NR)	CHBD	**CST/ERV** (1/3)
**Phee LM, 2015 [19] and 2019 [20]**	UK (1)	CHBD, TKA, PK/PD modelling	**CST/FA** (CHBD 3/3, TKA 1/1)
**Poulakou G, 2019 [21]**	Greece (1)	TKA, Intraperitoneal infection mouse model	**CST/DAP** (1/1)
**Shinohara DR, 2019 [22]**	Brazil (2)	CHBD, TKA, agar/disk, agar/gradient	**PMB/VAN** (CHBD 3/3, TKA 2/3, agar/disk 2/3, agar/gradient 3/3)
**Wang J, 2019 [23]**	China (4)	CHBD, TKA	<u>CHBD</u>: **MEM/VAN** (3/5), **MEM/SAM** (2/5), **MEM/TZB** (2/5), **MEM/CST** (4/5)<br>**TKA: MEM/CST** (2/2)
**Chen F, 2018 [24]**	China (1)	CHBD	**AMK/CIP** (4/34), **AMK/MEM** (12/34), **MEM/CIP** (3/34)
**Singham-In U, 2018 [25]**	Thailand (1)	CHBD, TKA	**MEM/AMK** (CHBD 2/2), **MEM/FOF** (CHBD 0/22), **IMP/AMK** (CHBD 1/2), **IMP/FOF** (CHBD 15/23, TKA 8/9)
**Zhu W, 2018 [26]**	China (1)	CHBD	**CST/IMP** (2/3), **CST/DOR** (0/2), **CST/FOF** (0/3), **CST/CFS** (0/3), **IMP/CFS** (11/16), **IMP/FOF** (11/20)
**Lenhard JR, 2017 [27,28]**	Thailand (1)	TKA, HFIM	**TKA**:<br>**PMB/MEM** (0/2), **PMB/SAM** (0/2), **MEM/SAM** (0/2), **PMB/MEM/SAM** (2/2)<br>**HFIM**:<br>PMB/MEM (0/1), PMB/SAM (0/1), MEM/SAM (0/1), PMB/MEM/SAM (1/1)
**Madadi-Goli N,**	Iran (1)	Fixed ratio E-test	**LVX/SAM** (7/7), **LVX/TGC** (0/7), **TGC/SAM**

Author-Year	Country (number of institutions from which the isolates were collected)	Methods for evaluation of synergy	Antimicrobial combinations tested (synergy present/number of eligible[1] strains)
**2017 [29]**		method	(0/7)
**Wei W, 2017 [30]**	China (1)	CHBD, TKA, G. mellonella model	**CST/LVX** (CHBD 0/1, TKA 0/1, G. mellonell 0/1)
**Wei WJ, 2017 [31]**	China (unclear)	CHBD, TKA	**CST/CHL** (CHBD 2/2, TKA 0/1)
**Bae S, 2016 [32]**	South Korea (1)	MCBT, CHBD (only the CST-based combinations)	**MCBT** for all possible combinations of: CST, SAM, AMK, AZM, ATM, MEM, RIF, TGC, SXT, VAN, TEC. Synergy shown only for the following: SAM/RIF (1/8), SAM/SXT (1/7), SAM/TEC (1/8), AMK/CAZ (1/6), AMK/SXT (1/6), AZM/CAZ (1/9), AZM/SXT (1/7), AZM/TEC (1/8), ATM/CAZ (1/9), ATM/SXT (1/7), ATM/TEC (1/9), CAZ/MEM (1/9), CAZ/RIF (1/9), CAZ/TGC (1/9), CAZ/SXT (1/7), CAZ/VAN (1/9), MEM/RIF (1/9), MEM/SXT (1/7), MEM/TEC (1/9), RIF/SXT (1/7), SXT/VAN (1/7), AMK/RIF (2/6), CAZ/TEC (2/9), CST/RIF (9/9), CST/TEC (9/9), CST/VAN (8/9), CST/MEM (8/9), CST/ATM (8/9), CST/CAZ (6/9), CST/SAM (5/8), CST/SXT (3/7), CST/AMK (4/6), CST/AZM (4/8), CST/TGC (0/9)<br>**CHBD**: CST/RIF (9/9), CST/TEC (4/9), CST/VAN (7/9), CST/MEM (3/9), CST/ATM (4/9), CST/CAZ (4/9), CST/SAM (0/8), CST/SXT (1/7), CST/AMK (2/6), CST/AZM (1/8), CST/TGC (0/9)
**Bowler SL, 2016 [33]**	USA (1)	CHBD, TKA	**CHBD**: **CST/FA** (3/3),<br>**TKA**: **CST/FA** (1/3), **CST/VAN** (1/3), **CST/DOR** (1/3)
**Hong DJ, 2016 [34]**	South Korea (1)	Fixed ratio E-test method	**CST/MEM** (41), **CST/IMP** (41), **CST/RIF** (41)
**Laishram S, 2016 [35]**	India (1)	CHBD, TKA	**MEM/SUL** (CHBD: 16/50, TKA: 29/50)
**Leite GC, 2016 [36]**	Brazil (unclear)	CHBD, (TKA data were excluded because reported data were not	**CST/RIF** (4/4), **CST/VAN** (7/7), **CST/MEM** (7/7), **CST/IMP** (7/7), **CST/TGC** (0/1), **CST/FOF** (0/6), **CST/GEN** (0/1), **FOF/GEN** (10/11), **FOF/AMK** (26/27)

Author-Year	Country (number of institutions from which the isolates were collected)	Methods for evaluation of synergy	Antimicrobial combinations tested (synergy present/number of eligible[1] strains)
		sufficient to allow accurate extraction)	
**Park GC, 2016 [37]**	South Korea (1***)	TKA	**CST/DOR** (10/17), **CST/TGC** (7/12), **TGC/DOR** (5/45)
**Yang H, 2016 [38]**	China (1)	CHBD, TKA, G. mellonella model	**CST/VAN** (1/1)
**Yang YS, 2016 [39]**	Taiwan (3)	CHBD, TKA	**MEM/MIN** (0/3), **MEM/CFS** (CHBD 0/2, TKA 1/2)
**Yavaş S, 2016 [40]**	Turkey (1)	Fixed ratio E-test method	**MEM/SUL** (1/7)
**Córdoba J, 2015 [41]**	Spain (2)	Dynamic in-vitro PK/PD model	**IMP/ETP** (0/3), **CST/DAP** (1/1), **CST/IMP** (0/1)
**García-Salguero C, 2015 [42]**	Spain (1)	Disk diffusion, CHBD, TKA	**AMK/IMP** (CHBD 2/2), **AMK/MEM** (CHBD 2/2), **AMK/FOF** (CHBD 0/2), **PLZ/IMP** (CHBD 6/8), **PLZ/MEM** (CHBD 2/4), **PLZ/FOF** (CHBD 1/9, TKA 1/2)
**Marie MA, 2015 [43]**	Saudi Arabia (1)	CHBD, E-test/agar method	**MEM/SUL** (CHBD 24/54, E-test 22/54), **MEM/TZB** (CHBD 22/54, E-test 19/54)
**Rodriguez CH, 2015 [44]**	Argentina (1)	TKA	**IMP/MIN** (0/1), **MIN/RIF** (0/1)
**Vourli S, 2015 [45]**	Greece (1)	CHBD	**CST/MEM** (2/2), **CST/SAM** (0/2), **MEM/SAM** (0/5)
**Galani I, 2014 [46]**	Greece (2)	E-test/agar dilution. TKA	**CST/DAP** (0/4)
**Majewski P, 2014 [47]**	Poland (1)	CHBD	**IMP/RIF** (4/10)
**Nastro M, 2014 [48]**	Argentina (unclear)	E-test/agar, TKA	**CST/RIF** (E-test/agar 4/4, TKA 4/4)
**Oleksium LM, 2014 [49]**	USA (1)	TKA	**CST/DOR** (5/6), **CST/SUL** (2/6), **CST/DOR/SUL** (6/6), **DOR/SUL** (4/17)
**Percin D, 2014 [50]**	Turkey (3)	CHBD, TKA	**CST/VAN** (CHBD 9/10, TKA 10/10)
**Sun Y, 2014 [51]**	China (1)	CHBD	**MEM/CFS** (0/11), **MEM/AMK** (0/9), **MEM/CIP** (1/11), **MEM/AZM** (0/12)
**Wang Y, 2014 [52]**	China (9)	CHBD, disk diffusion combination	**IMP/RIF** (12/18)
**Cetin ES, 2013 [53]**	Turkey (1)	CHBD, Perpendicular E-	**RIF/SAM** (7/7), **RIF/CFS** (2/7)

Author-Year	Country (number of institutions from which the isolates were collected)	Methods for evaluation of synergy	Antimicrobial combinations tested (synergy present/number of eligible[1] strains)
		test method	
**Housman ST, 2013 [54]**	USA (1)	Dynamic in-vitro PK/PD model	**DOR/SAM** (0/3), **DOR/TGC** (0/2), **SAM/TGC** (0/1)
**Lee HJ, 2013 [55]**	USA (1)	Dynamic in-vitro PK/PD model	**CST/RIF** (1/1)
**O'Hara JA, 2013 [56]**	USA (1)	CHBD, TKA, *G. mellonella* infection model	**CST/DOR** (CHBD 2/3, TKA 3/3, *G. mellonella* 0/3), **CST/VAN** (CHBD 3/3, TKA 2/3, *G. mellonella* 0/3), **VAN/DOR** (CHBD 0/3, TKA 0/3, *G. mellonella* 3/3), **CST/DOR/VAN** (TKA 3/3, *G. mellonella* 3/3)
**Principe L, 2013 [57]**	Italy (7)	CHBD, (TKA also conducted, but no eligible isolates)	**DOR/TGC** (3/3), **DOR/AMK** (1/4), **DOR/CST** (0/1), **DOR/SAM** (0/1), **DOR/RIF** (0/1)
**Queenan AM, 2013 [58]**	USA (1)	TKA, intraperitoneal infection mouse model	**DOR/CIP** (TKA 0/1, mouse model 0/1), **DOR/ LVX** (TKA 0/1, mouse model 1/1)
**Deveci A, 2012 [59]**	Turkey (1)	CHBD	**SUL/CAZ** (7/10), **SUL/MEM** (0/9), **SUL/CRO** (4/10), **SUL/CIP** (8/10), **SUL/GEN** (8/10), **SUL/FEP** (4/10)
**Peck KR, 2012 [60]**	South Korea (4)	TKA	**CST/RIF** (1/1), **CST/IMP** (0/2), **IMP/SAM** (6/6)
**Vidaillac C, 2012 [61]**	France (1)	CHBD, TKA	**CST/VAN** (CHBD 1/1, TKA 0.25xMIC 1/1, TKA 0.5xMIC 1/1), **CST/TMP** (CHBD 1/1, TKA 0.25xMIC 0/1, TKA 0.5xMIC 1/1), **CST/SXT** (CHBD 1/1, TKA 0.25xMIC 0/1, TKA 0.5xMIC 1/1)
**Santimaleeworagun W, 2011 [62]**	Thailand (1)	CHBD, TKA	**SUL/FOF** (CHBD 5/6, TKA 5/6), **SUL/IMP** (CHBD 0/6)
**Pachón-Ibáñez ME, 2011 [63]**	Spain (1)	TKA, pneumonia mouse models	**RIF/IMP** (TKA 2/2, animal model 0/2), **RIF/SUL** (TKA 2/2, animal model 1/2)
**Tan TY, 2011 [64]**	Singapore (4)	CHBD, perpendicular E-test method, TKA	**PMB/RIF** (CHBD 3/3, E-test 1/3, TKA 1/3), **PMB/TGC** (CHBD 3/4, E-test 0/4, TKA 0/4), **TGC/RIF** (CHBD 1/3, E-test 1/3, TKA 0/3)
**Kiratisin P, 2010 [65]**	Thailand (1)	Perpendicular E-test method	**DOR/CFS** (4/19), **DOR/DOX** (0/21), **DOR/RIF** (0/17), **DOR/NET** (0/21), **DOR/MXF** (0/21), **IMP/CFS** (9/19), **IMP/DOX** (0/21), **IMP/RIF** (0/17), **IMP/NET**

Author-Year	Country (number of institutions from which the isolates were collected)	Methods for evaluation of synergy	Antimicrobial combinations tested (synergy present/number of eligible[1] strains)
			(0/21), **IMP/MXF** (1/21), **MEM/CFS** (10/19), **MEM/DOX** (0/21), **MEM/RIF** (0/17), **MEM/NET** (0/21), **MEM/MXF** (0/21)
**Pachón-Ibáñez ME, 2010 [66]**	Spain (1)	TKA, mouse pneumonia and meningitis model	**SUL/IMP** (TKA: 2/4, model 1/1), **RIF/IMP** (TKA 2/4, model 0/1), **RIF/SUL** (TKA 1/4, model 0/1)
**Pankuch GA, 2010 [67]**	Germany (1)	TKA	**DOR/LVX** (3/5), **DOR/AMK** (2/2), **DOR/CST** (4/4)
**Rodriguez CH, 2010 [68]**	Argentina (1)	TKA	**IMP/RIF** (0/4), **IMP/GEN** (0/2)
**Urban C, 2010 [69]**	USA (1)	TKA	**DOR/RIF** (2/5)
**Yuan Z, 2010 [70] and Lim TP, 2008 [71]**	USA (1)	TKA (interactive index method), HFIM, mouse pneumonia model	**AMK/FEP** (TKA 1/1, HFIM 0/1, mouse model 0/1), **FEP/LVX** (TKA 1/1, mouse model 1/1), **AMK/LVX** (TKA 0/1, HFIM 0/1, mouse model 0/1)
**Lim TP, 2009 [72]**	Singapore (1)	TKA	**MEM/RIF** (2/2), **TGC/MEM** (0/1), **TGC/RIF** (1/1)
**Principe L, 2009 [73]**	Italy (5)	CHBD, TKA	**TGC/LVX** (CHBD 4/18, TKA 0/2), **TGC/TZP** (CHBD 0/18), **TGC/AMK** (CHBD 2/14, TKA 1/1), **TGC/IMP** (CHBD 1/12, TKA 0/1), **TGC/RIF** (CHBD 0/13), **TGC/SAM** (CHBD 0/6)
**Song YC, 2009 [74]**	South Korea (1)	Pneumonia mouse model	**IMP/RIF** (3/3), **RIF/AMK** (0/1), **IMP/AMK** (0/1)
**Lee CH, 2008 [75]**	Taiwan (1)	TKA	**MEM/SUL** (1/2)
**Lee NY, 2007 [76]**	Taiwan (3)	Agar dilution, CHBD	**IMP/SUL** (agar 4/4, CHBD 0/4), **MEM/SUL** (agar 4/4, CHBD 0/4)
**Sader HS, 2005 [77]**	USA (unclear)	CHBD	**FEP/SUL** (2/2)
**Sader HS, 2005 [78]**	USA (unclear)	TKA	**ATM/FEP** (0/3), **ATM/CAZ** (0/3), **ATM/MEM** (0/1), **ATM/SAM** (0/1)
**Choi JY, 2004 [79]**	South Korea (1)	TKA	**IMP/SUL** (1/1)
**Jung R, 2004 [80]**	USA (1)	TKA	**FEP/MXF** (2/2)
**Montero A, 2004 [81]**	Spain (1)	TKA, mouse pneumonia model	**IMP/RIF** (TKA 2/2, mouse model 1/2), **IMP/SUL** (TKA 0/1)
**Yoon J, 2004 [82]**	USA (1)	TKA	**PMB/IMP** (1/1)
**Fernández-Cuenca F, 2003 [83]**	Spain (3)	CHBD	**AZM/IMP** (0/2), **AZM/CAZ** (1/3), **AZM/AMK** (0/2), **AZM/CIP** (0/3)

**Author-Year**	**Country (number of institutions from which the isolates were collected)**	**Methods for evaluation of synergy**	**Antimicrobial combinations tested (synergy present/number of eligible[1] strains)**
**Roussel-Delvallez M, 1996 [84]**	France (1)	TKA	**IMP/SUL/AMK** (0/8), **IMP/TIM/AMK** (0/8), **TZP/SUL/AMK** (0/8), **TZP/TIM/AMK** (0/8)

Studies with overlapping isolates are grouped together (same row).

[1] Eligible= non-susceptible to the tested antimicrobial combinations. The number of eligible isolates can be different for each combination.

[2] See "Methods" for interpretation of clinical relevance

** 50 isolates randomly selected from a pool of 117 isolates, 107 of which were from diverse clonal lineages [9]

*** 64 of 69 isolates from a single hospital. 5 of 69 from the Korean Antimicrobial Resistance Monitoring System

2.2 Distribution of studies by year of publication

Year of publication	Number of studies (%)
**2017-2021**	**29 (35%)**
2021	5 (6%)
2020	6 (7%)
2019	10 (12%)
2018	3 (4%)
2017	5 (6%)
**2012-2016**	**32 (38%)**
2016	10 (12%)
2015	6 (7%)
2014	7 (8%)
2013	6 (7%)
2012	3 (4%)
**2007-2011**	**15 (18%)**
2011	3 (4%)
2010	6 (7%)
2009	3 (4%)
2008	2 (2%)
2007	1 (1%)
**2002-2006**	**7 (8%)**
2005	2 (2%)
2004	4 (5%)
2003	1 (1%)
**1995-2001**	**1 (1%)**
1996	1 (1%)

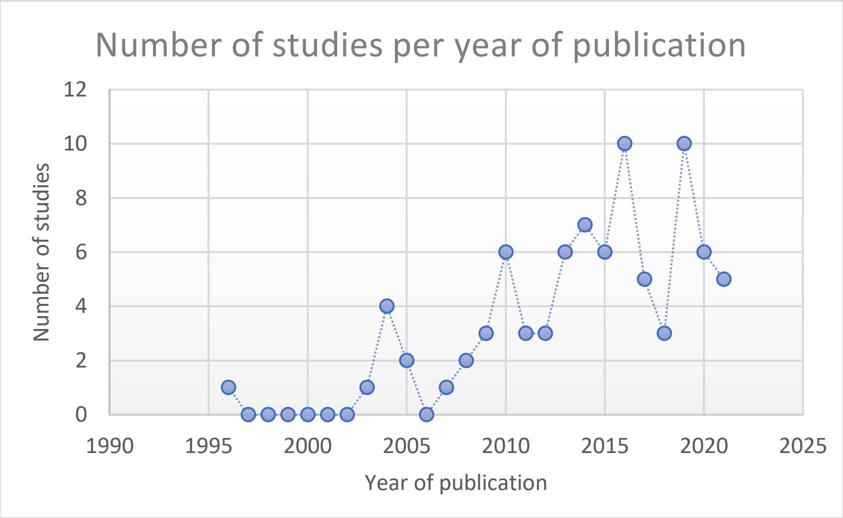

2.3 Distribution of studies by country and WHO regions

WHO regions	Number of studies per region (%)
**Americas**	**24 (29%)**
Brazil	6 (7%)
USA	12 (14%)
Argentina	3 (4%)
Colombia	1 (1%)
**South-East Asia Region**	7 (8%)
India	1 (1%)
Thailand	6 (7%)
**European Region**	**28 (33%)**
France	3 (4%)
Germany	1 (1%)
Greece	3 (4%)
Italy	3 (4%)
Spain	7 (8%)
Turkey	7 (8%)
Switzerland	1 (1%)
United Kingdom	1 (1%)
**Eastern Mediterranean Region**	**5 (6%)**
Iran	1 (1%)
Saudi Arabia	4 (5%)
United Arab Emirates	2 (2%)
Oman	2 (2%)
Kuwait	2 (2%)
Qatar	2 (2%)
Bahrain	3 (4%)
**Western Pacific Region**	**20 (24%)**
China	9 (11%)
South Korea	6 (7%)
Taiwan	3 (3%)

2.4 Number of eligible isolates per study

Number of eligible isolates	Number of studies (%)
1-5	51 (61%)
6-10	13 (16%)
11-15	2 (2%)
16-20	3 (4%)
21-25	4 (5%)
26-30	1 (1%)
31-35	1 (1%)
36-40	1 (1%)
41-45	1 (1%)
46-50	4 (5%)
51-55	2 (2%)
> 55 (80)	1 (1%)

2.5 Number of studies with single-centre vs multicentre design

Single-centre, n (%)	54 (64%)
Multicentre, n (%)	26 (31%)
• 2 centres	6 (7%)
• 3 centres	4 (5%)
• 4 centres	4 (5%)
• 5 centres	1 (1%)
• 7 centres	5 (6%)
• 9 centres	1 (1%)
• 15 centres	1 (1%)
• Unclear	4 (5%)
Unclear, n (%)	4 (5%)

3 Overview of number of studies and eligible isolate for each combinations evaluated

3.1 Summary table; Number of studies and eligible isolates per combination and method used

**Antimicrobial combinations**	**Total**		**CHBD**		**TKA**		**Gradient-based methods**		**Disk-based methods**		**Agar dilution**		**Dynamic in vitro PK/PD modelling**		**Animal models**	
	Studies	Isolates	Studies	Isolates	Studies	Isolates	Studies	Isolates	Studies	Isolates	Studies	Isolates	Studies	Isolates	Studies	Isolates
**AZM-based**																
AZM/CAZ	2	11	1	3												
AZM/CIP	1	3	1	3												
AZM/IMP	1	2	1	2												
AZM/MEM	1	12	1	12												
AZM/AMK	1	2	1	2												
AZM/CST	1	8	1	8												
AZM/SXT	1	6														
AZM/TEC	1	8														
**ATM-based**																
ATM/SAM	1	1			1	1										
ATM/CAZ	2	13			1	3										
ATM/FEP	1	3			1	3										
ATM/MEM	1	3			1	1										
ATM/CST	1	9	1	9												
ATM/SXT	1	7														
ATM/TEC	1	9														
**SUL-based**																
SUL/CRO	1	10	1	10												
SUL/CAZ	1	10	1	10												
SUL/FEP	1	9	1	9												
SUL/CIP	1	10	1	10												
SUL/IMP	5	16	2	10	3	6					1	4			1	1

Antimicrobial combinations	Total		CHBD		TKA		Gradient-based methods		Disk-based methods		Agar dilution		Dynamic in vitro PK/PD modelling		Animal models	
	Studies	Isolates	Studies	Isolates	Studies	Isolates	Studies	Isolates	Studies	Isolates	Studies	Isolates	Studies	Isolates	Studies	Isolates
SUL/MEM	8	181	6	173	3	54	2	61			1	4				
SUL/DOR	1	17			1	17										
SUL/AVI	1	38			1	1					1	38				
SUL/GEN	1	10	1	10												
SUL/CST	1	6			1	6										
SUL/PMB	1	3	1	3												
SUL/RIF	3	56	1	50	2	6									2	3
SUL/FOF	2	56	2	56	2	10										
**SAM-based**																
SAM/ATM	1	1			1	1										
SAM/FEP	1	2	1	2												
SAM/LVX	1	7	1	7												
SAM/IMP	1	6			1	6										
SAM/MEM	3	12	2	10	1	2							1	1		
SAM/DOR	2	7	1	1									1	3		
SAM/CST	2	10	2	10												
SAM/PMB	1	2			1	2							1	1		
SAM/TGC	3	14	2	13									1	1		
SAM/RIF	2	15	1	7			1	7								
SAM/SXT	1	7														
SAM/TEC	1	8														
**CSF-based**																
CFS/MXF	1	80	1	80			1	80								
CFS/IMP	2	35	1	16			1	19								
CFS/MEM	2	30	1	11			1	19								
CFS/DOR	1	19					1	19								

**Antimicrobial combinations**	**Total**		**CHBD**		**TKA**		**Gradient-based methods**		**Disk-based methods**		**Agar dilution**		**Dynamic in vitro PK/PD modelling**		**Animal models**	
	Studies	Isolates	Studies	Isolates	Studies	Isolates	Studies	Isolates	Studies	Isolates	Studies	Isolates	Studies	Isolates	Studies	Isolates
CFS/CST	1	3	1	3												
CFS/MIN	1	2	1	2	1	2										
CFS/RIF	1	7	1	7			1	7								
**TZB-based**																
TZB/MEM	2	59	2	59			1	54								
**TZP-based**																
TZP/TGC	1	18	1	18												
**CRO-based**																
CRO/SUL	1	10	1	10												
**CAZ-based**																
CAZ/AZM	2	11	1	3												
CAZ/ATM	2	13			1	3										
CAZ/SUL	1	10	1	10												
CAZ/MEM	1	9														
CAZ/AMK	1	6														
CAZ/CST	1	9	1	9												
CAZ/TGC	1	9														
CAZ/RIF	1	9														
CAZ/SXT	1	7														
CAZ/VAN	1	9														
CAZ/TEC	1	9														
**FEP-based**																
FEP/ATM	1	3			1	3										
FEP/SUL	1	9	1	9												
FEP/SAM	1	2	1	2												
FEP/LVX	1	1			1	1									1	1

Antimicrobial combinations	Total		CHBD		TKA		Gradient-based methods		Disk-based methods		Agar dilution		Dynamic in vitro PK/PD modelling		Animal models	
	Studies	Isolates	Studies	Isolates	Studies	Isolates	Studies	Isolates	Studies	Isolates	Studies	Isolates	Studies	Isolates	Studies	Isolates
FEP/MXF	1	2			1	2										
FEP/AMK	1	1			1	1							1	1	1	1
**CIP-based**																
CIP/AZM	1	3	1	3												
CIP/SUL	1	10	1	10												
CIP/MEM	2	45	2	45												
CIP/DOR	1	1			1	1									1	1
CIP/AMK	1	34	1	34												
CIP/FOF	1	2	1	2	1	1										
**LVX-based**																
LVX/SAM	1	7	1	7												
LVX/FEP	1	1			1	1									1	1
LVX/DOR	2	6			2	6									1	1
LVX/CZA	1	4			1	4										
LVX/AMK	1	1			1	1							1	1	1	1
LVX/CST	2	2	2	2	2	2									1	1
LVX/TGC	2	25	2	25	1	2										
LVX-FOF	1	1	1	1												
**MXF-based**																
MXF/CFS	1	80	1	80			1	80								
MXF/FEP	1	2			1	2										
MXF/IMP	1	21					1	21								
MXF/MEM	1	21					1	21								
MXF/DOR	1	21					1	21								
**ETP-based**																
ETP/IMP	1	3											1	3		

**Antimicrobial combinations**	**Total**		**CHBD**		**TKA**		**Gradient-based methods**		**Disk-based methods**		**Agar dilution**		**Dynamic in vitro PK/PD modelling**		**Animal models**	
	Studies	Isolates	Studies	Isolates	Studies	Isolates	Studies	Isolates	Studies	Isolates	Studies	Isolates	Studies	Isolates	Studies	Isolates
**IMP-based**																
IMP/AZM	1	2	1	2												
IMP/SUL	5	16	2	10	3	6					1	4			1	1
IMP/SAM	1	6			1	6										
IMP/CFS	2	35	1	16			1	19								
IMP/MXF	1	21					1	21								
IMP/ETP	1	3											1	3		
IMP/MEM	1	21	1	21											1	2
IMP/AMK	3	6	2	4											1	1
IMP/GEN	1	2			1	2										
IMP/NET	1	21					1	21								
IMP/PLZ	1	8	1	8												
IMP/CST	5	54	2	10	1	2	1	41					1	1		
IMP/PMB	1	1			1	1										
IMP/DOX	1	21					1	21								
IMP/TGC	1	12	1	12	1	1										
IMP/MIN	1	1			1	1										
IMP/RIF	9	61	2	28	5	13	1	17	1	1					4	8
IMP/FOF	3	45	3	45	1	9										
**MEM-based**																
MEM/AZM	1	12	1	12												
MEM/ATM	1	3			1	1										
MEM/SUL	8	181	6	173	3	54	2	61			1	4				
MEM/SAM	3	12	2	10	1	2							1	1		
MEM/CFS	2	30	1	11			1	19								
MEM/TZB	2	59	2	59			1	54								

**Antimicrobial combinations**	**Total**		**CHBD**		**TKA**		**Gradient-based methods**		**Disk-based methods**		**Agar dilution**		**Dynamic in vitro PK/PD modelling**		**Animal models**	
	Studies	Isolates	Studies	Isolates	Studies	Isolates	Studies	Isolates	Studies	Isolates	Studies	Isolates	Studies	Isolates	Studies	Isolates
MEM/CAZ	1	9														
MEM/CIP	2	45	2	45												
MEM/MXF	1	21					1	21								
MEM/IMP	1	21	1	21											1	2
MEM/CZA	2	15			2	15			1	11						
MEM/AMK	4	47	4	47												
MEM/NET	1	21					1	21								
MEM/PLZ	1	4	1	4												
MEM/CST	7	70	6	29	3	4	1	41								
MEM/PMB	2	5	1	3	1	2							1	1		
MEM/DOX	1	21			0	0	1	21								
MEM/TGC	1	1			1	1										
MEM/MIN	1	3	1	3	1	3										
MEM/RIF	3	69	1	50	1	2	1	17								
MEM/FOF	4	79	4	79												
MEM/SXT	1	7														
MEM/VAN	1	5	1	5												
MEM/TEC	1	9	0	0												
**DOR-based**																
DOR/SUL	1	17			1	17										
DOR/SAM	2	7	1	1									1	3		
DOR/CFS	1	19					1	19								
DOR/CIP	1	1			1	1									1	1
DOR/LVX	2	6			2	6									1	1
DOR/MXF	1	21					1	21								
DOR/AMK	2	6	1	4	1	2										

**Antimicrobial combinations**	**Total**		**CHBD**		**TKA**		**Gradient-based methods**		**Disk-based methods**		**Agar dilution**		**Dynamic in vitro PK/PD modelling**		**Animal models**	
	Studies	Isolates	Studies	Isolates	Studies	Isolates	Studies	Isolates	Studies	Isolates	Studies	Isolates	Studies	Isolates	Studies	Isolates
DOR/NET	1	21					1	21								
DOR/CST	7	37	3	6	5	33									1	3
DOR/DOX	1	21					1	21								
DOR/TGC	3	50	1	3	1	45							1	2		
DOR/RIF	3	23	1	1	1	5	1	17								
DOR/FOF	1	3	1	3	1	1										
DOR/VAN	1	3	1	3	1	3									1	3
**CZA- or AVI-based**																
AVI/SUL	1	38			1	1					1	38				
CZA/LVX	1	4			1	4										
CZA/MEM	2	15			2	15			1	11						
CZA/TOB	1	2			1	2										
CZA/CST	1	2			1	2										
CZA/TGC	1	4			1	4										
**AMK-based**																
AMK/AZM	1	2	1	2												
AMK/CAZ	1	6														
AMK/FEP	1	1			1	1							1	1	1	1
AMK/CIP	1	34	1	34												
AMK/LVX	1	1			1	1							1	1	1	1
AMK/IMP	3	6	2	4											1	1
AMK/MEM	4	47	4	47												
AMK/DOR	2	6	1	4	1	2										
AMK/CST	1	6	1	6												
AMK/TGC	1	14	1	14	1	1										

**Antimicrobial combinations**	**Total**		**CHBD**		**TKA**		**Gradient-based methods**		**Disk-based methods**		**Agar dilution**		**Dynamic in vitro PK/PD modelling**		**Animal models**	
	Studies	Isolates	Studies	Isolates	Studies	Isolates	Studies	Isolates	Studies	Isolates	Studies	Isolates	Studies	Isolates	Studies	Isolates
AMK/RIF	2	8													1	1
AMK/FOF	2	29	2	29												
AMK/SXT	1	6														
**GEN-based**																
GEN/SUL	1	10	1	10												
GEN/IMP	1	2			1	2										
GEN/CST	1	1	1	1												
GEN/FOF	2	13	2	13												
**TOB-based**																
TOB/CZA	1	2			1	2										
TOB/FOF	1	2	1	2												
**NET-based**																
NET/IMP	1	21					1	21								
NET/MEM	1	21					1	21								
NET/DOR	1	21					1	21								
**PLZ-based**																
PLZ/IMP	1	8	1	8												
PLZ/MEM	1	4	1	4												
PLZ/FOF	1	9	1	9	1	2										
**CST-based**																
CST/AZM	1	8	1	8												
CST/ATM	1	9	1	9												
CST/SUL	1	6			1	6										
CST/SAM	2	10	2	10												
CST/CFS	1	3	1	3												
CST/CAZ	1	9	1	9												

**Antimicrobial combinations**	**Total**		**CHBD**		**TKA**		**Gradient-based methods**		**Disk-based methods**		**Agar dilution**		**Dynamic in vitro PK/PD modelling**		**Animal models**	
	Studies	Isolates	Studies	Isolates	Studies	Isolates	Studies	Isolates	Studies	Isolates	Studies	Isolates	Studies	Isolates	Studies	Isolates
CST/LVX	2	2	2	2	2	2									1	1
CST/IMP	5	54	2	10	1	2	1	41					1	1		
CST/MEM	7	70	6	29	3	4	1	41								
CST/DOR	7	37	3	6	5	33									1	3
CST/CZA	1	2			1	2										
CST/AMK	1	6	1	6												
CST/GEN	1	1	1	1												
CST/TGC	3	22	2	10	1	12										
CST/ERV	1	3	1	3												
CST/RIF	9	87	5	40	3	7	2	45					1	1		
CST/RFB	1	3	1	3												
CST/FOF	2	9	2	9												
CST/TMP	1	1	1	1	1	1										
CST/SXT	2	9	2	8	1	1										
CST/CHL	1	2	1	2	1	2										
CST/FA	2	6	2	6	2	4			1	3						
CST/VAN	8	36	7	33	6	20									2	4
CST/TEC	1	9	1	9												
CST/DAP	3	6			2	5	1	4					1	1	1	1
**PMB-based**																
PMB/SUL	1	3	1	3												
PMB/SAM	1	2	0	0	1	2							1	1		
PMB/IMP	1	1	0	0	1	1										
PMB/MEM	2	5	1	3	1	2							1	1		
PMB/TGC	1	4	1	4	1	4	1	4								
PMB/RIF	1	3	1	3	1	3	1	3								

Antimicrobial combinations	Total		CHBD		TKA		Gradient-based methods		Disk-based methods		Agar dilution		Dynamic in vitro PK/PD modelling		Animal models	
	Studies	Isolates	Studies	Isolates	Studies	Isolates	Studies	Isolates	Studies	Isolates	Studies	Isolates	Studies	Isolates	Studies	Isolates
PMB/FOF	1	3	1	3												
PMB/VAN	1	3	1	3	1	3	1	3	1	3						
**DOX-based**																
DOX/IMP	1	21					1	21								
DOX/MEM	1	21					1	21								
DOX/DOR	1	21					1	21								
**TGC-based**																
TGC/SAM	3	14	2	13									1	1		
TGC/TZP	1	18	1	18												
TGC/CAZ	1	9														
TGC/LVX	2	25	2	25	1	2										
TGC/IMP	1	12	1	12	1	1										
TGC/MEM	1	1	0	0	1	1										
TGC/DOR	3	50	1	3	1	45							1	2		
TGC/CZA	1	4			1	4										
TGC/AMK	1	14	1	14	1	1										
TGC/CST	3	22	2	10	1	12										
TGC/PMB	1	4	1	4	1	4	1	4								
TGC/RIF	3	17	2	16	2	4	1	3								
TGC/FOF	1	4	1	4	1	1										
**MIN-based**																
MIN/CFS	1	2	1	2	1	2										
MIN/IMP	1	1			1	1										
MIN/MEM	1	3	1	3	1	3										
**ERV-based**																
ERV/CST	1	3	1	3												

Antimicrobial combinations	Total		CHBD		TKA		Gradient-based methods		Disk-based methods		Agar dilution		Dynamic in vitro PK/PD modelling		Animal models	
	Studies	Isolates	Studies	Isolates	Studies	Isolates	Studies	Isolates	Studies	Isolates	Studies	Isolates	Studies	Isolates	Studies	Isolates
**RIF-based**																
RIF/SUL	3	56	1	50	2	6									2	3
RIF/SAM	2	15	1	7			1	7								
RIF/CFS	1	7	1	7			1	7								
RIF/CAZ	1	9														
RIF/IMP	9	61	2	28	5	13	1	17	1	1					4	8
RIF/MEM	3	69	1	50	1	2	1	17								
RIF/DOR	3	23	1	1	1	5	1	17								
RIF/AMK	2	8													1	1
RIF/CST	9	87	5	40	3	7	2	45					1	1		
RIF/PMB	1	3	1	3	1	3	1	3								
RIF/TGC	3	17	2	16	2	4	1	3								
RIF/FOF	1	50	1	50												
RIF/SXT	1	7														
RIF/LZD	1	3	1	3												
**RFB-based**																
RFB/CST	1	3	1	3												
**FOF-based**																
FOF/SUL	2	56	2	56	2	10										
FOF/CIP	1	1	1	1												
FOF/LVX	1	1	1	1												
FOF/IMP	3	45	3	45	1	9										
FOF/MEM	4	79	4	79												
FOF/DOR	1	3	1	3	1	1										
FOF/AMK	2	29	2	29												
FOF/GEN	2	13	2	13												

**Antimicrobial combinations**	**Total**		**CHBD**		**TKA**		**Gradient-based methods**		**Disk-based methods**		**Agar dilution**		**Dynamic in vitro PK/PD modelling**		**Animal models**	
	Studies	Isolates	Studies	Isolates	Studies	Isolates	Studies	Isolates	Studies	Isolates	Studies	Isolates	Studies	Isolates	Studies	Isolates
FOF/TOB	1	2	1	2												
FOF/PLZ	1	9	1	9	1	2										
FOF/CST	2	9	2	9												
FOF/PMB	1	3	1	3												
FOF/TGC	1	4	1	4	1	1										
FOF/RIF	1	50	1	50												
**TMP-based**																
TMP/CST	1	1	1	1	1	1										
**SXT-based**																
SXT/AZM	1	6														
SXT/ATM	1	7														
SXT/SAM	1	7														
SXT/CAZ	1	7														
SXT/MEM	1	7														
SXT/AMK	1	6														
SXT/CST	2	9	2	8	1	1										
SXT/RIF	1	7														
SXT/VAN	1	7														
**CHL-based**																
CHL/CST	1	2	1	2	1	2										
**FA-based**																
FA/CST	2	6	2	6	2	4			1	3						
**VAN-based**																
VAN/CAZ	1	9														
VAN/MEM	1	5	1	5												
VAN/DOR	1	3	1	3	1	3									1	3

Antimicrobial combinations	Total		CHBD		TKA		Gradient-based methods		Disk-based methods		Agar dilution		Dynamic in vitro PK/PD modelling		Animal models	
	Studies	Isolates	Studies	Isolates	Studies	Isolates	Studies	Isolates	Studies	Isolates	Studies	Isolates	Studies	Isolates	Studies	Isolates
VAN/CST	8	36	7	33	6	20									2	4
VAN/PMB	1	3	1	3	1	3	1	3	1	3						
VAN/SXT	1	7														
**TEC-based**																
TEC/AZM	1	8														
TEC/ATM	1	9														
TEC/SAM	1	8														
TEC/CAZ	1	9														
TEC/MEM	1	9														
TEC/CST	1	9	1	9												
**DAP-based**																
DAP/CST	3	6			2	5	1	4					1	1	1	1
**LZD-based**																
LZD/RIF	1	3	1	3												
**Triple combinations**																
PMB/FOF/MEM	1	3	1	3												
PMB/SUL/MEM	1	3	1	3												
PMB/SAM/MEM	2	2			2	2							1	1		
CST/DOR/SUL	1	6			1	6										
CST/VAN/DOR	1	3			1	3									1	3
IMP/SUL/AMK	1	8			1	8										
IMP/TIM/AMK	1	8			1	8										
TZP/SUL/AMK	1	8			1	8										
TZP/TIM/AMK	1	8			1	8										

MCBT assay was used in only 1 study and is not shown in the Table. See Section 4.5 (Bae S, 2016 [32])

3.2 Few studies available for each combination

Number of studies	Number of combinations
1	98
2	22
3	11
4	2
5	2
6	0
7	2
8	2
9	2

I.e. there were; only 1 study available for 98 of the 141 double combinations evaluated, 2 studies for 22 of the combinations, 3 studies for 11 of the combinations, 4 studies for 2 of the combinations, 5 studies for 2 of the combinations, 7 studies for 2 of the combinations, 8 studies for 2 of the combinations and 9 studies for 2 of the combinations.

3.3 Number of studies per combination (only double combinations shown)

	AZM	ATM	SUL	SAM	CFS	TZB	TZP	CRO	CAZ	FEP	CIP	LVX	MXF	ETP	IMP	MEM	DOR	CZA	AVI	AMK	GEN	TOB	NET	PLZ	CST	PMB	DOX	TGC	MIN	ERV	RIF	RFB	FOF	TMP	SXT	CHL	FA	VAN
AZM		0	0	0	0	0	0	0	2	0	1	0	0	0	1	1	0	0	0	1	0	0	0	0	1	0	0	0	0	0	0	0	0	0	1	0	0	0
ATM	0		0	1	0	0	0	0	2	1	0	0	0	0	0	1	0	0	0	0	0	0	0	0	1	0	0	0	0	0	0	0	0	0	1	0	0	0
SUL	0	0				0	0	1	1	1	1	0	0	0	5	8	1	0	1	0	1	0	0	0	1	1	0	0	0	0	3	0	2	0	0	0	0	0
SAM	0	1				0	0	0	0	1	0	1	0	0	1	3	2	0	0	0	0	0	0	0	2	1	0	3	0	0	2	0	0	0	1	0	0	0
CFS	0	0				0	0	0	0	0	0	0	1	0	2	2	1	0	0	0	0	0	0	0	1	0	0	0	1	0	1	0	0	0	0	0	0	0
TZB	0	0	0	0	0		0	0	0	0	0	0	0	0	0	2	0	0	0	0	0	0	0	0	0	0	0	0	0	0	0	0	0	0	0	0	0	0
TZP	0	0	0	0	0	0		0	0	0	0	0	0	0	0	0	0	0	0	0	0	0	0	0	0	0	0	1	0	0	0	0	0	0	0	0	0	0
CRO	0	0	1	0	0	0	0		0	0	0	0	0	0	0	0	0	0	0	0	0	0	0	0	0	0	0	0	0	0	0	0	0	0	0	0	0	0
CAZ	2	2	1	0	0	0	0	0		0	0	0	0	0	0	1	0	0	0	1	0	0	0	0	1	0	0	1	0	0	1	0	0	0	1	0	0	1
FEP	0	1	1	1	0	0	0	0	0		0	1	1	0	0	0	0	0	0	1	0	0	0	0	0	0	0	0	0	0	0	0	0	0	0	0	0	0
CIP	1	0	1	0	0	0	0	0	0	0		0	0	0	0	2	1	0	0	1	0	0	0	0	0	0	0	0	0	0	0	0	1	0	0	0	0	0
LVX	0	0	0	1	0	0	0	0	0	1	0		0	0	0	0	2	1	0	1	0	0	0	0	2	0	0	2	0	0	0	0	1	0	0	0	0	0
MXF	0	0	0	0	1	0	0	0	0	1	0	0		0	1	1	1	0	0	0	0	0	0	0	0	0	0	0	0	0	0	0	0	0	0	0	0	0
ETP	0	0	0	0	0	0	0	0	0	0	0	0	0		1	0	0	0	0	0	0	0	0	0	0	0	0	0	0	0	0	0	0	0	0	0	0	0
IMP	1	0	5	1	2	0	0	0	0	0	0	0	1	1		1	0	0	0	3	1	0	1	1	5	1	1	1	1	0	9	0	3	0	0	0	0	0
MEM	1	1	8	3	2	2	0	0	1	0	2	0	1	1	1		0	2	0	3	0	0	1	1	7	2	1	1	1	0	4	0	4	0	1	0	0	1
DOR	0	0	1	2	1	0	0	0	0	0	1	2	1	0	0	0		0	0	2	0	0	1	0	7	0	1	3	0	0	3	0	1	0	0	0	0	1
CZA	0	0	0	0	0	0	0	0	0	0	0	1	0	0	0	2	0		0	0	0	1	0	0	1	0	0	1	0	0	0	0	0	0	0	0	0	0
AVI	0	0	1	0	0	0	0	0	0	0	0	0	0	0	0	0	0	0		0	0	0	0	0	0	0	0	0	0	0	0	0	0	0	0	0	0	0
AMK	1	0	0	0	0	0	0	0	1	1	1	1	0	0	3	3	2	0	0		0	0	0	0	1	0	0	1	0	0	2	0	2	0	1	0	0	0
GEN	0	0	1	0	0	0	0	0	0	0	0	0	0	0	1	0	0	0	0	0		0	0	0	1	0	0	0	0	0	0	0	2	0	0	0	0	0
TOB	0	0	0	0	0	0	0	0	0	0	0	0	0	0	0	0	0	1	0	0	0		0	0	0	0	0	0	0	0	0	0	1	0	0	0	0	0
NET	0	0	0	0	0	0	0	0	0	0	0	0	0	0	1	1	1	0	0	0	0	0		0	0	0	0	0	0	0	0	0	0	0	0	0	0	0
PLZ	0	0	0	0	0	0	0	0	0	0	0	0	0	0	1	1	0	0	0	0	0	0			0	0	0	0	0	0	0	0	1	0	0	0	0	0
CST	1	1	1	2	1	0	0	0	1	0	0	2	0	0	5	7	7	1	0	1	1	0	0	0			0	3	0	1	9	1	2	1	2	1	2	8
PMB	0	0	1	1	0	0	0	0	0	0	0	0	0	0	1	2	0	0	0	0	0	0	0	0			0	1	0	0	1	0	1	0	0	0	0	1
DOX	0	0	0	0	0	0	0	0	0	0	0	0	0	0	1	1	1	0	0	0	0	0	0	0	0	0		0	0	0	0	0	0	0	0	0	0	0
TGC	0	0	0	3	0	0	1	0	1	0	0	2	0	0	1	1	3	1	0	1	0	0	0	0	3	1	0				3	0	1	0	0	0	0	0
MIN	0	0	0	0	1	0	0	0	0	0	0	0	0	0	1	1	0	0	0	0	0	0	0	0	0	0	0				0	0	0	0	0	0	0	0
ERV	0	0	0	0	0	0	0	0	0	0	0	0	0	0	0	0	0	0	0	0	0	0	0	0	1	0	0				0	0	0	0	0	0	0	0
RIF	0	0	3	2	1	0	0	0	1	0	0	0	0	0	9	4	3	0	0	2	0	0	0	0	9	1	0	3	0	0			1	0	1	0	0	0

	AZM	ATM	SUL	SAM	CFS	TZB	TZP	CRO	CAZ	FEP	CIP	LVX	MXF	ETP	IMP	MEM	DOR	CZA	AVI	AMK	GEN	TOB	NET	PLZ	CST	PMB	DOX	TGC	MIN	ERV	RIF	RFB	FOF	TMP	SXT	CHL	FA	VAN
RFB	0	0	0	0	0	0	0	0	0	0	0	0	0	0	0	0	0	0	0	0	0	0	0	0	1	0	0	0	0	0			0	0	0	0	0	0
FOF	0	0	2	0	0	0	0	0	0	0	1	1	0	0	3	4	1	0	0	2	2	1	0	1	2	1	0	1	0	0	1	0		0	0	0	0	0
TMP	0	0	0	0	0	0	0	0	0	0	0	0	0	0	0	0	0	0	0	0	0	0	0	0	1	0	0	0	0	0	0	0	0			0	0	0
SXT	1	1	0	1	0	0	0	0	1	0	0	0	0	0	0	1	0	0	0	1	0	0	0	0	2	0	0	0	0	0	1	0	0			0	0	1
CHL	0	0	0	0	0	0	0	0	0	0	0	0	0	0	0	0	0	0	0	0	0	0	0	0	1	0	0	0	0	0	0	0	0	0	0		0	0
FA	0	0	0	0	0	0	0	0	0	0	0	0	0	0	0	0	0	0	0	0	0	0	0	0	2	0	0	0	0	0	0	0	0	0	0	0		0
VAN	0	0	0	0	0	0	0	0	1	0	0	0	0	0	0	1	1	0	0	0	0	0	0	0	8	1	0	0	0	0	0	0	0	0	1	0	0	
TEC	1	1	0	1	0	0	0	0	1	0	0	0	0	0	0	1	0	0	0	0	0	0	0	0	1	0	0	0	0	0	0	0	0	0	0	0	0	0
DAP	0	0	0	0	0	0	0	0	0	0	0	0	0	0	0	0	0	0	0	0	0	0	0	0	3	0	0	0	0	0	0	0	0	0	0	0	0	0
LZD	0	0	0	0	0	0	0	0	0	0	0	0	0	0	0	0	0	0	0	0	0	0	0	0	0	0	0	0	0	0	1	0	0	0	0	0	0	0

	1 study		6 studies
	2 studies		7 studies
	3 studies		8 studies
	4 studies		9 studies

3.4 Number of eligible isolates per combination (only double combinations shown)

	AZM	ATM	SUL	SAM	CFS	TZB	TZP	CRO	CAZ	FEP	CIP	LVX	MXF	ETP	IMP	MEM	DOR	CZA	AVI	AMK	GEN	TOB	NET	PLZ	CST	PMB	DOX	TGC	MIN	ERV	RIF	RFB	FOF	TMP	SXT	CHL	FA	VAN
AZM		0	0	0	0	0	0	0	11	0	3	0	0	0	2	12	0	0	0	2	0	0	0	0	8	0	0	0	0	0	0	0	0	0	6	0	0	0
ATM	0		0	1	0	0	0	0	13	3	0	0	0	0	0	3	0	0	0	0	0	0	0	0	9	0	0	0	0	0	0	0	0	0	7	0	0	0
SUL	0	0				0	0	10	10	9	10	0	0	0	16	181	17	0	38	0	10	0	0	0	6	3	0	0	0	0	56	0	56	0	0	0	0	0
SAM	0	1				0	0	0	0	2	0	7	0	0	6	12	7	0	0	0	0	0	0	0	10	2	0	14	0	0	15	0	0	0	7	0	0	0
CFS	0	0				0	0	0	0	0	0	0	80	0	35	30	19	0	0	0	0	0	0	0	3	0	0	0	2	0	7	0	0	0	0	0	0	0
TZB	0	0	0	0	0		0	0	0	0	0	0	0	0	0	59	0	0	0	0	0	0	0	0	0	0	0	0	0	0	0	0	0	0	0	0	0	0
TZP	0	0	0	0	0	0		0	0	0	0	0	0	0	0	0	0	0	0	0	0	0	0	0	0	0	0	18	0	0	0	0	0	0	0	0	0	0
CRO	0	0	10	0	0	0	0		0	0	0	0	0	0	0	0	0	0	0	0	0	0	0	0	0	0	0	0	0	0	0	0	0	0	0	0	0	0
CAZ	11	13	10	0	0	0	0	0		0	0	0	0	0	0	9	0	0	0	6	0	0	0	0	9	0	0	9	0	0	9	0	0	0	7	0	0	9
FEP	0	3	9	2	0	0	0	0	0		0	1	2	0	0	0	0	0	0	1	0	0	0	0	0	0	0	0	0	0	0	0	0	0	0	0	0	0
CIP	3	0	10	0	0	0	0	0	0	0		0	0	0	0	45	1	0	0	34	0	0	0	0	0	0	0	0	0	0	0	0	2	0	0	0	0	0
LVX	0	0	0	7	0	0	0	0	0	1	0		0	0	0	0	6	4	0	1	0	0	0	0	2	0	0	25	0	0	0	0	1	0	0	0	0	0
MXF	0	0	0	0	80	0	0	0	0	2	0	0		0	21	21	21	0	0	0	0	0	0	0	0	0	0	0	0	0	0	0	0	0	0	0	0	0
ETP	0	0	0	0	0	0	0	0	0	0	0	0	0		3	0	0	0	0	0	0	0	0	0	0	0	0	0	0	0	0	0	0	0	0	0	0	0
IMP	2	0	16	6	35	0	0	0	0	0	0	0	21	3		21	0	0	0	6	2	0	21	8	54	1	21	12	1	0	61	0	45	0	0	0	0	0
MEM	12	3	181	12	30	59	0	0	9	0	45	0	21	0	21		0	15	0	45	0	0	21	4	70	5	21	1	3	0	78	0	79	0	7	0	0	5
DOR	0	0	17	7	19	0	0	0	0	0	1	6	21	0	0	0		0	0	6	0	0	21	0	37	0	21	50	0	0	23	0	3	0	0	0	0	3
CZA	0	0	0	0	0	0	0	0	0	0	0	4	0	0	0	15	0		0	0	0	2	0	0	2	0	0	4	0	0	0	0	0	0	0	0	0	0
AVI	0	0	38	0	0	0	0	0	0	0	0	0	0	0	0	0	0	0		0	0	0	0	0	0	0	0	0	0	0	0	0	0	0	0	0	0	0
AMK	2	0	0	0	0	0	0	0	6	1	34	1	0	0	6	45	6	0	0		0	0	0	0	6	0	0	14	0	0	8	0	29	0	6	0	0	0
GEN	0	0	10	0	0	0	0	0	0	0	0	0	0	0	2	0	0	0	0	0		0	0	0	1	0	0	0	0	0	0	0	13	0	0	0	0	0
TOB	0	0	0	0	0	0	0	0	0	0	0	0	0	0	0	0	0	2	0	0	0		0	0	0	0	0	0	0	0	0	0	2	0	0	0	0	0
NET	0	0	0	0	0	0	0	0	0	0	0	0	0	0	21	21	21	0	0	0	0	0		0	0	0	0	0	0	0	0	0	0	0	0	0	0	0
PLZ	0	0	0	0	0	0	0	0	0	0	0	0	0	0	8	4	0	0	0	0	0	0	0		0	0	0	0	0	0	0	0	9	0	0	0	0	0
CST	8	9	6	10	3	0	0	0	9	0	0	2	0	0	54	70	37	2	0	6	1	0	0	0			0	22	0	3	87	3	9	1	9	2	6	36
PMB	0	0	3	2	0	0	0	0	0	0	0	0	0	0	1	5	0	0	0	0	0	0	0	0			0	4	0	0	3	0	3	0	0	0	0	3
DOX	0	0	0	0	0	0	0	0	0	0	0	0	0	0	21	21	21	0	0	0	0	0	0	0				0	0	0	0	0	0	0	0	0	0	0
TGC	0	0	0	14	0	0	18	0	9	0	0	25	0	0	12	1	50	4	0	14	0	0	0	0	22	4					17	0	4	0	0	0	0	0
MIN	0	0	0	0	2	0	0	0	0	0	0	0	0	0	1	3	0	0	0	0	0	0	0	0	0	0	0				0	0	0	0	0	0	0	0
ERV	0	0	0	0	0	0	0	0	0	0	0	0	0	0	0	0	0	0	0	0	0	0	0	0	3	0	0				0	0	0	0	0	0	0	0
RIF	0	0	56	15	7	0	0	0	9	0	0	0	0	0	61	78	23	0	0	8	0	0	0	0	87	3	0	17	0	0			50	0	7	0	0	0
RFB	0	0	0	0	0	0	0	0	0	0	0	0	0	0	0	0	0	0	0	0	0	0	0	0	3	0	0	0	0	0			0	0	0	0	0	0

	AZM	ATM	SUL	SAM	CFS	TZB	TZP	CRO	CAZ	FEP	CIP	LVX	MXF	ETP	IMP	MEM	DOR	CZA	AVI	AMK	GEN	TOB	NET	PLZ	CST	PMB	DOX	TGC	MIN	ERV	RIF	RFB	FOF	TMP	SXT	CHL	FA	VAN
FOF	0	0	56	0	0	0	0	0	0	0	2	1	0	0	45	79	3	0	0	29	13	2	0	9	9	3	0	4	0	0	50	0		0	0	0	0	0
TMP	0	0	0	0	0	0	0	0	0	0	0	0	0	0	0	0	0	0	0	0	0	0	0	0	1	0	0	0	0	0	0	0	0			0	0	0
SXT	6	7	0	7	0	0	0	0	7	0	0	0	0	0	0	7	0	0	0	6	0	0	0	0	9	0	0	0	0	0	7	0	0			0	0	7
CHL	0	0	0	0	0	0	0	0	0	0	0	0	0	0	0	0	0	0	0	0	0	0	0	0	2	0	0	0	0	0	0	0	0	0	0		0	0
FA	0	0	0	0	0	0	0	0	0	0	0	0	0	0	0	0	0	0	0	0	0	0	0	0	6	0	0	0	0	0	0	0	0	0	0	0		0
VAN	0	0	0	0	0	0	0	0	9	0	0	0	0	0	0	5	3	0	0	0	0	0	0	0	36	3	0	0	0	0	0	0	0	0	7	0	0	
TEC	8	9	0	8	0	0	0	0	9	0	0	0	0	0	0	9	0	0	0	0	0	0	0	0	9	0	0	0	0	0	0	0	0	0	0	0	0	0
DAP	0	0	0	0	0	0	0	0	0	0	0	0	0	0	0	0	0	0	0	0	0	0	0	0	6	0	0	0	0	0	0	0	0	0	0	0	0	0
LZD	0	0	0	0	0	0	0	0	0	0	0	0	0	0	0	0	0	0	0	0	0	0	0	0	0	0	0	0	0	0	3	0	0	0	0	0	0	0

	1-5		21-30
	6-10		31-40
	11-15		41-50
	16-20		51-60
			>60

3.5 Number of studies per combinations (only double combinations shown) tested by checkerboard method

	AZM	ATM	SUL	SAM	CFS	TZB	TZP	CRO	CAZ	FEP	CIP	LVX	MXF	IMP	MEM	DOR	AMK	GEN	TOB	PLZ	CST	PMB	TGC	MIN	ERV	RIF	RFB	FOF	TMP	SXT	CHL	FA
AZM		0	0	0	0	0	0	0	1	0	1	0	0	1	1	0	1	0	0	0	1	0	0	0	0	0	0	0	0	0	0	0
ATM	0		0	0	0	0	0	0	0	0	0	0	0	0	0	0	0	0	0	0	1	0	0	0	0	0	0	0	0	0	0	0
SUL	0	0				0	0	1	1	1	1	0	0	2	6	0	0	1	0	0	0	1	0	0	0	1	0	2	0	0	0	0
SAM	0	0				0	0	0	0	1	0	1	0	0	2	1	0	0	0	0	2	0	2	0	0	1	0	0	0	0	0	0
CFS	0	0				0	0	0	0	0	0	0	1	1	1	0	0	0	0	0	1	0	0	1	0	1	0	0	0	0	0	0
TZB	0	0	0	0	0		0	0	0	0	0	0	0	0	2	0	0	0	0	0	0	0	0	0	0	0	0	0	0	0	0	0
TZP	0	0	0	0	0	0		0	0	0	0	0	0	0	0	0	0	0	0	0	0	0	1	0	0	0	0	0	0	0	0	0
CRO	0	0	1	0	0	0	0		0	0	0	0	0	0	0	0	0	0	0	0	0	0	0	0	0	0	0	0	0	0	0	0
CAZ	1	0	1	0	0	0	0	0		0	0	0	0	0	0	0	0	0	0	0	1	0	0	0	0	0	0	0	0	0	0	0
FEP	0	0	1	1	0	0	0	0	0		0	0	0	0	0	0	0	0	0	0	0	0	0	0	0	0	0	0	0	0	0	0
CIP	1	0	1	0	0	0	0	0	0	0		0	0	0	2	0	1	0	0	0	0	0	0	0	0	0	0	1	0	0	0	0
LVX	0	0	0	1	0	0	0	0	0	0	0		0	0	0	0	0	0	0	0	2	0	2	0	0	0	0	1	0	0	0	0
MXF	0	0	0	0	1	0	0	0	0	0	0	0		0	0	0	0	0	0	0	0	0	0	0	0	0	0	0	0	0	0	0
IMP	1	0	2	0	1	0	0	0	0	0	0	0	0		1	0	2	0	0	1	2	0	1	0	0	2	0	3	0	0	0	0
MEM	1	0	6	2	1	2	0	0	0	0	2	0	0	1		0	3	0	0	1	6	1	0	1	0	1	0	4	0	0	0	0
DOR	0	0	0	1	0	0	0	0	0	0	0	0	0	0	0		1	0	0	0	3	0	1	0	0	1	0	1	0	0	0	0
AMK	1	0	0	0	0	0	0	0	0	0	1	0	0	2	3	1		0	0	0	1	0	1	0	0	0	0	2	0	0	0	0
GEN	0	0	1	0	0	0	0	0	0	0	0	0	0	0	0	0	0		0	0	1	0	0	0	0	0	0	2	0	0	0	0
TOB	0	0	0	0	0	0	0	0	0	0	0	0	0	0	0	0	0	0		0	0	0	0	0	0	0	0	1	0	0	0	0
PLZ	0	0	0	0	0	0	0	0	0	0	0	0	0	1	1	0	0	0	0		0	0	0	0	0	0	0	1	0	0	0	0
CST	1	1	0	2	1	0	0	0	1	0	0	2	0	2	6	3	1	1	0	0			2	0	1	5	1	2	1	2	1	2
PMB	0	0	1	0	0	0	0	0	0	0	0	0	0	0	1	0	0	0	0	0			1	0	0	1	0	1	0	0	0	0
TGC	0	0	0	2	0	0	1	0	0	0	0	2	0	1	0	1	1	0	0	0	2	1				2	0	1	0	0	0	0
MIN	0	0	0	0	1	0	0	0	0	0	0	0	0	0	1	0	0	0	0	0	0	0				0	0	0	0	0	0	0
ERV	0	0	0	0	0	0	0	0	0	0	0	0	0	0	0	0	0	0	0	0	1	0				0	0	0	0	0	0	0
RIF	0	0	1	1	1	0	0	0	0	0	0	0	0	2	1	1	0	0	0	0	5	1	2	0	0			1	0	0	0	0
RFB	0	0	0	0	0	0	0	0	0	0	0	0	0	0	0	0	0	0	0	0	1	0	0	0	0			0	0	0	0	0
FOF	0	0	2	0	0	0	0	0	0	0	1	1	0	3	4	1	2	2	1	1	2	1	1	0	0	1	0		0	0	0	0
TMP	0	0	0	0	0	0	0	0	0	0	0	0	0	0	0	0	0	0	0	0	1	0	0	0	0	0	0	0			0	0
SXT	0	0	0	0	0	0	0	0	0	0	0	0	0	0	0	0	0	0	0	0	2	0	0	0	0	0	0	0			0	0
CHL	0	0	0	0	0	0	0	0	0	0	0	0	0	0	0	0	0	0	0	0	1	0	0	0	0	0	0	0	0	0		0
FA	0	0	0	0	0	0	0	0	0	0	0	0	0	0	0	0	0	0	0	0	2	0	0	0	0	0	0	0	0	0	0	
VAN	0	0	0	0	0	0	0	0	0	0	0	0	0	0	1	1	0	0	0	0	7	1	0	0	0	0	0	0	0	0	0	0
TEC	0	0	0	0	0	0	0	0	0	0	0	0	0	0	0	0	0	0	0	0	1	0	0	0	0	0	0	0	0	0	0	0
LZD	0	0	0	0	0	0	0	0	0	0	0	0	0	0	0	0	0	0	0	0	0	0	0	0	0	1	0	0	0	0	0	0

	1 study		6 studies
	2 studies		7 studies
	3 studies		8 studies
	4 studies		9 studies

3.6 Number of eligible isolates per combination (only double combinations are shown) tested by checkerboard method

	AZM	ATM	SUL	SAM	CFS	TZB	TZP	CRO	CAZ	FEP	CIP	LVX	MXF	IMP	MEM	DOR	AMK	GEN	TOB	PLZ	CST	PMB	TGC	MIN	ERV	RIF	RFB	FOF	TMP	SXT	CHL	FA	VAN
AZM		0	0	0	0	0	0	0	3	0	3	0	0	2	12	0	2	0	0	0	8	0	0	0	0	0	0	0	0	0	0	0	0
ATM	0		0	0	0	0	0	0	0	0	0	0	0	0	0	0	0	0	0	0	9	0	0	0	0	0	0	0	0	0	0	0	0
SUL	0	0				0	0	10	10	9	10	0	0	10	173	0	0	10	0	0	0	3	0	0	0	50	0	56	0	0	0	0	0
SAM	0	0				0	0	0	0	2	0	7	0	0	10	1	0	0	0	0	10	0	13	0	0	7	0	0	0	0	0	0	0
CFS	0	0				0	0	0	0	0	0	0	80	16	11	0	0	0	0	0	3	0	0	2	0	7	0	0	0	0	0	0	0
TZB	0	0	0	0	0		0	0	0	0	0	0	0	0	59	0	0	0	0	0	0	0	0	0	0	0	0	0	0	0	0	0	0
TZP	0	0	0	0	0	0		0	0	0	0	0	0	0	0	0	0	0	0	0	0	0	18	0	0	0	0	0	0	0	0	0	0
CRO	0	0	10	0	0	0	0		0	0	0	0	0	0	0	0	0	0	0	0	0	0	0	0	0	0	0	0	0	0	0	0	0
CAZ	3	0	10	0	0	0	0	0		0	0	0	0	0	0	0	0	0	0	0	9	0	0	0	0	0	0	0	0	0	0	0	0
FEP	0	0	9	2	0	0	0	0	0		0	0	0	0	0	0	0	0	0	0	0	0	0	0	0	0	0	0	0	0	0	0	0
CIP	3	0	10	0	0	0	0	0	0	0		0	0	0	45	0	34	0	0	0	0	0	0	0	0	0	0	2	0	0	0	0	0
LVX	0	0	0	7	0	0	0	0	0	0	0		0	0	0	0	0	0	0	0	2	0	25	0	0	0	0	1	0	0	0	0	0
MXF	0	0	0	0	80	0	0	0	0	0	0	0		0	0	0	0	0	0	0	0	0	0	0	0	0	0	0	0	0	0	0	0
IMP	2	0	10	0	16	0	0	0	0	0	0	0	0		21	0	4	0	0	8	10	0	12	0	0	28	0	45	0	0	0	0	0
MEM	12	0	173	10	11	59	0	0	0	0	45	0	0	21		0	45	0	0	4	29	3	0	3	0	50	0	79	0	0	0	0	5
DOR	0	0	0	1	0	0	0	0	0	0	0	0	0	0	0		4	0	0	0	6	0	3	0	0	1	0	3	0	0	0	0	3
AMK	2	0	0	0	0	0	0	0	0	0	34	0	0	4	45	4		0	0	0	6	0	14	0	0	0	0	29	0	0	0	0	0
GEN	0	0	10	0	0	0	0	0	0	0	0	0	0	0	0	0	0		0	0	1	0	0	0	0	0	0	13	0	0	0	0	0
TOB	0	0	0	0	0	0	0	0	0	0	0	0	0	0	0	0	0	0		0	0	0	0	0	0	0	0	2	0	0	0	0	0
PLZ	0	0	0	0	0	0	0	0	0	0	0	0	0	8	4	0	0	0	0		0	0	0	0	0	0	0	9	0	0	0	0	0
CST	8	9	0	10	3	0	0	0	9	0	0	2	0	10	29	6	6	1	0	0			10	0	3	40	3	9	1	8	2	6	33
PMB	0	0	3	0	0	0	0	0	0	0	0	0	0	0	3	0	0	0	0	0			4	0	0	3	0	3	0	0	0	0	3
TGC	0	0	0	13	0	0	18	0	0	0	0	25	0	12	0	3	14	0	0	0	10	4				16	0	4	0	0	0	0	0
MIN	0	0	0	0	2	0	0	0	0	0	0	0	0	0	3	0	0	0	0	0	0	0				0	0	0	0	0	0	0	0
ERV	0	0	0	0	0	0	0	0	0	0	0	0	0	0	0	0	0	0	0	0	3	0				0	0	0	0	0	0	0	0
RIF	0	0	50	7	7	0	0	0	0	0	0	0	0	28	50	1	0	0	0	0	40	3	16	0	0			50	0	0	0	0	0
RFB	0	0	0	0	0	0	0	0	0	0	0	0	0	0	0	0	0	0	0	0	3	0	0	0	0			0	0	0	0	0	0
FOF	0	0	56	0	0	0	0	0	0	0	2	1	0	45	79	3	29	13	2	9	9	3	4	0	0	50	0		0	0	0	0	0
TMP	0	0	0	0	0	0	0	0	0	0	0	0	0	0	0	0	0	0	0	0	1	0	0	0	0	0	0	0			0	0	0
SXT	0	0	0	0	0	0	0	0	0	0	0	0	0	0	0	0	0	0	0	0	8	0	0	0	0	0	0	0			0	0	0
CHL	0	0	0	0	0	0	0	0	0	0	0	0	0	0	0	0	0	0	0	0	2	0	0	0	0	0	0	0	0	0		0	0
FA	0	0	0	0	0	0	0	0	0	0	0	0	0	0	0	0	0	0	0	0	6	0	0	0	0	0	0	0	0	0	0		0
VAN	0	0	0	0	0	0	0	0	0	0	0	0	0	0	5	3	0	0	0	0	33	3	0	0	0	0	0	0	0	0	0	0	
TEC	0	0	0	0	0	0	0	0	0	0	0	0	0	0	0	0	0	0	0	0	9	0	0	0	0	0	0	0	0	0	0	0	0
LZD	0	0	0	0	0	0	0	0	0	0	0	0	0	0	0	0	0	0	0	0	0	0	0	0	0	3	0	0	0	0	0	0	0

	1-5		21-30
	6-10		31-40
	11-15		41-50
	16-20		51-60
			>60

3.7 Number of eligible studies (only double combinations shown) tested by TKA

	ATM	SUL	SAM	CFS	CAZ	FEP	CIP	LVX	MXF	IMP	MEM	DOR	CZA	AVI	AMK	GEN	TOB	PLZ	CST	PMB	TGC	MIN	RIF	FOF	TMP	SXT	CHL
ATM		0	1	0	1	1	0	0	0	0	1	0	0	0	0	0	0	0	0	0	0	0	0	0	0	0	0
SUL	0				0	0	0	0	0	3	3	1	0	1	0	0	0	0	1	0	0	0	2	2	0	0	0
SAM	1				0	0	0	0	0	1	1	0	0	0	0	0	0	0	0	1	0	0	0	0	0	0	0
CFS	0				0	0	0	0	0	0	0	0	0	0	0	0	0	0	0	0	0	1	0	0	0	0	0
CAZ	1	0	0	0		0	0	0	0	0	0	0	0	0	0	0	0	0	0	0	0	0	0	0	0	0	0
FEP	1	0	0	0	0		0	1	1	0	0	0	0	0	1	0	0	0	0	0	0	0	0	0	0	0	0
CIP	0	0	0	0	0	0		0	0	0	0	1	0	0	0	0	0	0	0	0	0	0	0	1	0	0	0
LVX	0	0	0	0	0	1	0		0	0	0	2	1	0	1	0	0	0	2	0	1	0	0	0	0	0	0
MXF	0	0	0	0	0	1	0	0		0	0	0	0	0	0	0	0	0	0	0	0	0	0	0	0	0	0
IMP	0	3	1	0	0	0	0	0	0		0	0	0	0	0	1	0	0	1	1	1	1	5	1	0	0	0
MEM	1	3	1	0	0	0	0	0	0	0		0	2	0	0	0	0	0	3	1	1	1	1	0	0	0	0
DOR	0	1	0	0	0	0	1	2	0	0	0		0	0	1	0	0	0	5	0	1	0	1	1	0	0	0
CZA	0	0	0	0	0	0	0	1	0	0	2	0		0	0	0	1	0	1	0	1	0	0	0	0	0	0
AVI	0	1	0	0	0	0	0	0	0	0	0	0	0		0	0	0	0	0	0	0	0	0	0	0	0	0
AMK	0	0	0	0	0	1	0	1	0	0	0	1	0	0		0	0	0	0	0	1	0	0	0	0	0	0
GEN	0	0	0	0	0	0	0	0	0	1	0	0	0	0	0		0	0	0	0	0	0	0	0	0	0	0
TOB	0	0	0	0	0	0	0	0	0	0	0	0	1	0	0	0		0	0	0	0	0	0	0	0	0	0
PLZ	0	0	0	0	0	0	0	0	0	0	0	0	0	0	0	0	0		0	0	0	0	0	1	0	0	0
CST	0	1	0	0	0	0	0	2	0	1	3	5	1	0	0	0	0	0			1	0	3	0	1	1	1
PMB	0	0	1	0	0	0	0	0	0	1	1	0	0	0	0	0	0	0			1	0	1	0	0	0	0
TGC	0	0	0	0	0	0	0	1	0	1	1	1	1	0	1	0	0	0	1	1			2	1	0	0	0
MIN	0	0	0	1	0	0	0	0	0	1	1	0	0	0	0	0	0	0	0	0			0	0	0	0	0
RIF	0	2	0	0	0	0	0	0	0	5	1	1	0	0	0	0	0	0	3	1	2	0		0	0	0	0
FOF	0	2	0	0	0	0	1	0	0	1	0	1	0	0	0	0	0	1	0	0	1	0	0		0	0	0
TMP	0	0	0	0	0	0	0	0	0	0	0	0	0	0	0	0	0	0	1	0	0	0	0	0			0
SXT	0	0	0	0	0	0	0	0	0	0	0	0	0	0	0	0	0	0	1	0	0	0	0	0			0
CHL	0	0	0	0	0	0	0	0	0	0	0	0	0	0	0	0	0	0	1	0	0	0	0	0	0	0	
FA	0	0	0	0	0	0	0	0	0	0	0	0	0	0	0	0	0	0	2	0	0	0	0	0	0	0	0
VAN	0	0	0	0	0	0	0	0	0	0	0	1	0	0	0	0	0	0	6	1	0	0	0	0	0	0	0
DAP	0	0	0	0	0	0	0	0	0	0	0	0	0	0	0	0	0	0	2	0	0	0	0	0	0	0	0

	1 study		6 studies
	2 studies		7 studies
	3 studies		8 studies
	4 studies		9 studies

3.8 Number of eligible isolates (only double combinations shown) tested by TKA

	ATM	SUL	SAM	CFS	CAZ	FEP	CIP	LVX	MXF	IMP	MEM	DOR	CZA	AVI	AMK	GEN	TOB	PLZ	CST	PMB	TGC	MIN	RIF	FOF	TMP	SXT	CHL
ATM		0	1	0	3	3	0	0	0	0	1	0	0	0	0	0	0	0	0	0	0	0	0	0	0	0	0
SUL	0				0	0	0	0	0	6	54	17	0	1	0	0	0	0	6	0	0	0	6	10	0	0	0
SAM	1				0	0	0	0	0	6	2	0	0	0	0	0	0	0	0	2	0	0	0	0	0	0	0
CFS	0				0	0	0	0	0	0	0	0	0	0	0	0	0	0	0	0	0	2	0	0	0	0	0
CAZ	3	0	0	0		0	0	0	0	0	0	0	0	0	0	0	0	0	0	0	0	0	0	0	0	0	0
FEP	3	0	0	0	0		0	1	2	0	0	0	0	0	1	0	0	0	0	0	0	0	0	0	0	0	0
CIP	0	0	0	0	0	0		0	0	0	0	1	0	0	0	0	0	0	0	0	0	0	0	1	0	0	0
LVX	0	0	0	0	0	1	0		0	0	0	6	4	0	1	0	0	0	2	0	2	0	0	0	0	0	0
MXF	0	0	0	0	0	2	0	0		0	0	0	0	0	0	0	0	0	0	0	0	0	0	0	0	0	0
IMP	0	6	6	0	0	0	0	0	0		0	0	0	0	0	2	0	0	2	1	1	1	13	9	0	0	0
MEM	1	54	2	0	0	0	0	0	0	0		0	15	0	0	0	0	0	4	2	1	3	2	0	0	0	0
DOR	0	17	0	0	0	0	1	6	0	0	0		0	0	2	0	0	0	33	0	45	0	5	1	0	0	0
CZA	0	0	0	0	0	0	0	4	0	0	15	0		0	0	0	2	0	2	0	4	0	0	0	0	0	0
AVI	0	1	0	0	0	0	0	0	0	0	0	0	0		0	0	0	0	0	0	0	0	0	0	0	0	0
AMK	0	0	0	0	0	1	0	1	0	0	0	2	0	0		0	0	0	0	0	1	0	0	0	0	0	0
GEN	0	0	0	0	0	0	0	0	0	2	0	0	0	0	0		0	0	0	0	0	0	0	0	0	0	0
TOB	0	0	0	0	0	0	0	0	0	0	0	0	2	0	0	0		0	0	0	0	0	0	0	0	0	0
PLZ	0	0	0	0	0	0	0	0	0	0	0	0	0	0	0	0	0		0	0	0	0	0	2	0	0	0
CST	0	6	0	0	0	0	0	2	0	2	4	33	2	0	0	0	0	0			12	0	7	0	1	1	2
PMB	0	0	2	0	0	0	0	0	0	1	2	0	0	0	0	0	0	0			4	0	3	0	0	0	0
TGC	0	0	0	0	0	0	0	2	0	1	1	45	4	0	1	0	0	0	12	4			4	1	0	0	0
MIN	0	0	0	2	0	0	0	0	0	1	3	0	0	0	0	0	0	0	0	0			0	0	0	0	0
RIF	0	6	0	0	0	0	0	0	0	13	2	5	0	0	0	0	0	0	7	3	4	0		0	0	0	0
FOF	0	10	0	0	0	0	1	0	0	9	0	1	0	0	0	0	0	2	0	0	1	0	0		0	0	0
TMP	0	0	0	0	0	0	0	0	0	0	0	0	0	0	0	0	0	0	1	0	0	0	0	0			0
SXT	0	0	0	0	0	0	0	0	0	0	0	0	0	0	0	0	0	0	1	0	0	0	0	0			0
CHL	0	0	0	0	0	0	0	0	0	0	0	0	0	0	0	0	0	0	2	0	0	0	0	0	0	0	
FA	0	0	0	0	0	0	0	0	0	0	0	0	0	0	0	0	0	0	4	0	0	0	0	0	0	0	0
VAN	0	0	0	0	0	0	0	0	0	0	0	3	0	0	0	0	0	0	20	3	0	0	0	0	0	0	0
DAP	0	0	0	0	0	0	0	0	0	0	0	0	0	0	0	0	0	0	5	0	0	0	0	0	0	0	0

	1-5		21-30
	6-10		31-40
	11-15		41-50
	16-20		51-60
			>60

3.9 Number of eligible studies tested by gradient methods

	SUL	SAM	CFS	TZB	MXF	IMP	MEM	DOR	NET	CST	PMB	DOX	TGC	RIF	VAN
SUL				0	0	0	2	0	0	0	0	0	0	0	0
SAM				0	0	0	0	0	0	0	0	0	0	1	0
CFS				0	1	1	1	1	0	0	0	0	0	1	0
TZB	0	0	0		0	0	1	0	0	0	0	0	0	0	0
MXF	0	0	1	0		1	1	1	0	0	0	0	0	0	0
IMP	0	0	1	0	1		0	0	1	1	0	1	0	1	0
MEM	2	0	1	1	1	0		0	1	1	0	1	0	1	0
DOR	0	0	1	0	1	0	0		1	0	0	1	0	1	0
NET	0	0	0	0	0	1	1	1		0	0	0	0	0	0
CST	0	0	0	0	0	1	1	0	0			0	0	2	0
PMB	0	0	0	0	0	0	0	0	0			0	1	1	1
DOX	0	0	0	0	0	1	1	1	0	0	0		0	0	0
TGC	0	0	0	0	0	0	0	0	0	0	1	0		1	0
RIF	0	1	1	0	0	1	1	1	0	2	1	0	1		0
VAN	0	0	0	0	0	0	0	0	0	0	1	0	0	0	
DAP	0	0	0	0	0	0	0	0	0	1	0	0	0	0	0

	1 study		6 studies
	2 studies		7 studies
	3 studies		8 studies
	4 studies		9 studies

3.10 Number of eligible isolates tested by gradient methods

	SUL	SAM	CFS	TZB	MXF	IMP	MEM	DOR	CST	PMB	DOX	TGC	RIF	VAN	DAP
SUL				0	0	0	61	0	0	0	0	0	0	0	0
SAM				0	0	0	0	0	0	0	0	0	7	0	0
CFS				0	80	19	19	19	0	0	0	0	7	0	0
TZB	0	0	0		0	0	54	0	0	0	0	0	0	0	0
MXF	0	0	80	0		21	21	21	0	0	0	0	0	0	0
IMP	0	0	19	0	21		0	0	41	0	21	0	17	0	0
MEM	61	0	19	54	21	0		0	41	0	21	0	17	0	0
DOR	0	0	19	0	21	0	0		0	0	21	0	17	0	0
CST	0	0	0	0	0	41	41	0			0	0	45	0	4
PMB	0	0	0	0	0	0	0	0			0	4	3	3	0
DOX	0	0	0	0	0	21	21	21	0	0		0	0	0	0
TGC	0	0	0	0	0	0	0	0	0	4	0		3	0	0
RIF	0	7	7	0	0	17	17	17	45	3	0	3		0	0
VAN	0	0	0	0	0	0	0	0	0	3	0	0	0		0
DAP	0	0	0	0	0	0	0	0	4	0	0	0	0	0	

	1-5		21-30
	6-10		31-40
	11-15		41-50
	16-20		51-60
			>60

3.11 Number of eligible studies tested by disk methods

	IMP	MEM	CZA	CST	PMB	RIF	FA	VAN
IMP		0	0	0	0	1	0	0
MEM	0		1	0	0	0	0	0
CZA	0	1		0	0	0	0	0
CST	0	0	0			0	1	0
PMB	0	0	0			0	0	1
RIF	1	0	0	0	0		0	0
FA	0	0	0	1	0	0		0
VAN	0	0	0	0	1	0	0	

	1 study		6 studies
	2 studies		7 studies
	3 studies		8 studies
	4 studies		9 studies

3.12 Number of eligible isolates tested by disk methods

	IMP	MEM	CZA	CST	PMB	RIF	FA	VAN
IMP		0	0	0	0	1	0	0
MEM	0		11	0	0	0	0	0
CZA	0	11		0	0	0	0	0
CST	0	0	0			0	3	0
PMB	0	0	0			0	0	3
RIF	1	0	0	0	0		0	0
FA	0	0	0	3	0	0		0
VAN	0	0	0	0	3	0	0	

	1-5		21-30
	6-10		31-40
	11-15		41-50
	16-20		51-60
			>60

3.13 Number of eligible studies tested by agar dilution method

	SUL	IMP	MEM	AVI
SUL		1	1	1
IMP	1		0	0
MEM	1	0		0
AVI	1	0	0	

	1 study		6 studies
	2 studies		7 studies
	3 studies		8 studies
	4 studies		9 studies

3.14 Number of eligible isolates tested by agar dilution method

	SUL	IMP	MEM	AVI
SUL		4	4	38
IMP	4		0	0
MEM	4	0		0
AVI	38	0	0	

	1-5		21-30
	6-10		31-40
	11-15		41-50
	16-20		51-60
			>60

3.15 Number of eligible studies (only double combinations shown) tested by dynamic in vitro PK/PD models

	SAM	FEP	LVX	ETP	IMP	MEM	DOR	AMK	CST	PMB	TGC	RIF	DAP
SAM		0	0	0	0	1	1	0	0	1	1	0	0
FEP	0		0	0	0	0	0	1	0	0	0	0	0
LVX	0	0		0	0	0	0	1	0	0	0	0	0
ETP	0	0	0		1	0	0	0	0	0	0	0	0
IMP	0	0	0	1		0	0	0	1	0	0	0	0
MEM	1	0	0	0	0		0	0	0	1	0	0	0
DOR	1	0	0	0	0	0		0	0	0	1	0	0
AMK	0	1	1	0	0	0	0		0	0	0	0	0
CST	0	0	0	0	1	0	0	0			0	1	1
PMB	1	0	0	0	0	1	0	0			0	0	0
TGC	1	0	0	0	0	0	1	0	0	0		0	0
MIN	0	0	0	0	0	0	0	0	0	0		0	0
ERV	0	0	0	0	0	0	0	0	0	0		0	0
RIF	0	0	0	0	0	0	0	0	1	0	0		0
DAP	0	0	0	0	0	0	0	0	1	0	0	0	

	1 study		6 studies
	2 studies		7 studies
	3 studies		8 studies
	4 studies		9 studies

3.16 Number of eligible isolates (only double combinations shown) tested by dynamic in vitro PK/PD models

	SAM	FEP	LVX	ETP	IMP	MEM	DOR	AMK	CST	PMB	TGC	RIF	DAP
SAM		0	0	0	0	1	3	0	0	1	1	0	0
FEP	0		0	0	0	0	0	1	0	0	0	0	0
LVX	0	0		0	0	0	0	1	0	0	0	0	0
ETP	0	0	0		3	0	0	0	0	0	0	0	0
IMP	0	0	0	3		0	0	0	1	0	0	0	0
MEM	1	0	0	0	0		0	0	0	1	0	0	0
DOR	3	0	0	0	0	0		0	0	0	2	0	0
AMK	0	1	1	0	0	0	0		0	0	0	0	0
CST	0	0	0	0	1	0	0	0			0	1	1
PMB	1	0	0	0	0	1	0	0			0	0	0
TGC	1	0	0	0	0	0	2	0	0	0		0	0
RIF	0	0	0	0	0	0	0	0	1	0	0		0
DAP	0	0	0	0	0	0	0	0	1	0	0	0	

	1-5		21-30
	6-10		31-40
	11-15		41-50
	16-20		51-60
			>60

3.17 Number of eligible studies (only double combinations shown) tested by animal models

	SUL	FEP	CIP	LVX	IMP	MEM	DOR	AMK	CST	RIF	VAN	DAP
SUL		0	0	0	1	0	0	0	0	2	0	0
FEP	0		0	1	0	0	0	1	0	0	0	0
CIP	0	0		0	0	0	1	0	0	0	0	0
LVX	0	1	0		0	0	1	1	1	0	0	0
IMP	1	0	0	0		1	0	1	0	4	0	0
MEM	0	0	0	0	1		0	0	0	0	0	0
DOR	0	0	1	1	0	0		0	1	0	1	0
AMK	0	1	0	1	1	0	0		0	1	0	0
CST	0	0	0	1	0	0	1	0		0	2	1
RIF	2	0	0	0	4	0	0	1	0		0	0
VAN	0	0	0	0	0	0	1	0	2	0		0
DAP	0	0	0	0	0	0	0	0	1	0	0	

	1 study		6 studies
	2 studies		7 studies
	3 studies		8 studies
	4 studies		9 studies

3.18 Number of eligible isolates (only double combinations shown) tested by animal models

	SUL	FEP	CIP	LVX	IMP	MEM	DOR	AMK	CST	RIF	VAN	DAP
SUL		0	0	0	1	0	0	0	0	3	0	0
FEP	0		0	1	0	0	0	1	0	0	0	0
CIP	0	0		0	0	0	1	0	0	0	0	0
LVX	0	1	0		0	0	1	1	1	0	0	0
IMP	1	0	0	0		2	0	1	0	8	0	0
MEM	0	0	0	0	2		0	0	0	0	0	0
DOR	0	0	1	1	0	0		0	3	0	3	0
AMK	0	1	0	1	1	0	0		0	1	0	0
CST	0	0	0	1	0	0	3	0		0	4	1
RIF	3	0	0	0	8	0	0	1	0		0	0
VAN	0	0	0	0	0	0	3	0	4	0		0

DAP	0	0	0	0	0	0	0	0	1	0	0	

	1-5		21-30
	6-10		31-40
	11-15		41-50
	16-20		51-60
			>60

4 Assessment of clinical relevance of observed synergy

4.1 Summary Table (sum of all studies): Proportion of synergy and clinically relevant synergy in checkerboard (CHBD) and time-kill assay (TKA)

Antimicrobial combinations	CHBD			CHBD: concentration at which synergy was present			TKA			TKA: concentration at which synergy was present		
	Studies N	Isolates n	Synergy n (%)	≤ breakpoints n (%)	> breakpoints n (%)	Unclear n (%)	Studies N	Isolates n	Synergy n (%)	≤ breakpoints n (%)	> breakpoints n (%)	Unclear n (%)
**AZM-based**												
AZM/CAZ	1	3	1 (33%)	0 (0%)	1 (33%)	0 (0%)						
AZM/CIP	1	3	0 (0%)	0 (0%)	0 (0%)	0 (0%)						
AZM/IMP	1	2	0 (0%)	0 (0%)	0 (0%)	0 (0%)						
AZM/MEM	1	12	0 (0%)	0 (0%)	0 (0%)	0 (0%)						
AZM/AMK	1	2	0 (0%)	0 (0%)	0 (0%)	0 (0%)						
AZM/CST	1	8	1 (13%)	0 (0%)	0 (0%)	1 (13%)						
**ATM-based**												
ATM/SAM							1	1	0 (0%)	0 (0%)	0 (0%)	0 (0%)
ATM/CAZ							1	3	0 (0%)	0 (0%)	0 (0%)	0 (0%)
ATM/FEP							1	3	0 (0%)	0 (0%)	0 (0%)	0 (0%)
ATM/MEM							1	1	0 (0%)	0 (0%)	0 (0%)	0 (0%)
ATM/CST	1	9	4 (44%)	0 (0%)	0 (0%)	4 (44%)						
**SUL-based**												
SUL/CRO	1	10	4 (40%)	0 (0%)	4 (40%)	0 (0%)						
SUL/CAZ	1	10	7 (70%)	1 (10%)	6 (60%)	0 (0%)						
SUL/FEP	1	9	4 (44%)	0 (0%)	4 (44%)	0 (0%)						
SUL/CIP	1	10	8 (80%)	2 (20%)	6 (60%)	0 (0%)						
SUL/IMP	2	10	0 (0%)	0 (0%)	0 (0%)	0 (0%)	3	6	3 (50%)	0 (0%)	3(50%)	0 (0%)

Antimicrobial combinations	CHBD			CHBD: concentration at which synergy was present			TKA			TKA: concentration at which synergy was present		
	**Studies N**	**Isolates n**	**Synergy n (%)**	**≤ breakpoints n (%)**	**> breakpoints n (%)**	**Unclear n (%)**	**Studies N**	**Isolates n**	**Synergy n (%)**	**≤ breakpoints n (%)**	**> breakpoints n (%)**	**Unclear n (%)**
SUL/MEM	6	173	72 (42%)	2 (1%)	2 (1%)	68 (39%)	3	54	32 (59%)	0 (0%)	7 (13%)	25 (46%)
SUL/DOR							1	17	4 (24%)	4 (24%)	0 (0%)	0 (0%)
SUL/AVI							1	1	1 (100%)	1 (100%)	0 (0%)	0 (0%)
SUL/GEN	1	10	8 (80%)	2 (20%)	6 (60%)	0 (0%)						
SUL/CST							1	6	2 (33%)	2 (33%)	0 (0%)	0 (0%)
SUL/PMB	1	3	2 (67%)	2 (67%)	0 (0%)	0 (0%)						
SUL/RIF	1	50	10 (20%)	0 (0%)	0 (0%)	10 (20%)	2	6	3 (50%)	0 (0%)	3 (50%)	0 (0%)
SUL/FOF	2	56	41 (73%)	3 (5%)	37 (66%)	1 (2%)	2	10	7 (70%)	0 (0%)	7 (70%)	0 (0%)
**SAM-based**												
SAM/ATM							1	1	0 (0%)	0 (0%)	0 (0%)	0 (0%)
SAM/FEP	1	2	2 (100%)	1 (50%)	1 (50%)	0 (0%)						
SAM/LVX	1	7	7 (100%)	5 (71%)	2 (29%)	0 (0%)						
SAM/IMP							1	6	6 (100%)	0 (0%)	0 (0%)	6 (100%)
SAM/MEM	2	10	2 (100%)	2 (100%)	0 (0%)	0 (0%)	1	2	0 (0%)	0 (0%)	0 (0%)	0 (0%)
SAM/DOR	1	1	0 (0%)	0 (0%)	0 (0%)	0 (0%)						
SAM/CST	2	10	0 (0%)	0 (0%)	0 (0%)	0 (0%)						
SAM/PMB							1	2	0 (0%)	0 (0%)	0 (0%)	0 (0%)
SAM/TGC	2	13	0 (0%)	0 (0%)	0 (0%)	0 (0%)						
SAM/RIF	1	7	7 (100%)	4 (57%)	1 (14%)	2 (29%)						
**CSF-based**												
CFS/MXF	1	80	4 (5%)	0 (0%)	0 (0%)	4 (5%)						
CFS/IMP	1	16	11 (69%)	9 (56%)	2 (13%)	0 (0%)						
CFS/MEM	1	11	0 (0%)	0 (0%)	0 (0%)	0 (0%)						
CFS/CST	1	3	0 (0%)	0 (0%)	0 (0%)	0 (0%)						

Antimicrobial combinations	CHBD			CHBD: concentration at which synergy was present			TKA			TKA: concentration at which synergy was present		
	Studies N	Isolates n	Synergy n (%)	≤ breakpoints n (%)	> breakpoints n (%)	Unclear n (%)	Studies N	Isolates n	Synergy n (%)	≤ breakpoints n (%)	> breakpoints n (%)	Unclear n (%)
CFS/MIN	1	2	0 (0%)	0 (0%)	0 (0%)	0 (0%)	1	2	1 (50%)	0 (0%)	1 (50%)	0 (0%)
CFS/RIF	1	7	2 (29%)	1 (14%)	0 (0%)	1 (14%)						
**TZB-based**												
TZB/MEM	2	59	24 (41%)	0 (0%)	0 (0%)	24 (41%)						
**TZP-based**												
TZP/TGC	1	18	0 (0%)	0 (0%)	0 (0%)	0 (0%)						
**CRO-based**												
CRO/SUL	1	10	4 (40%)	0 (0%)	4 (40%)	0 (0%)						
**CAZ-based**												
CAZ/AZM	1	3	1 (33%)	0 (0%)	1 (33%)	0 (0%)						
CAZ/ATM							1	3	0 (0%)	0 (0%)	0 (0%)	0 (0%)
CAZ/SUL	1	10	7 (70%)	1 (10%)	6 (60%)	0 (0%)						
CAZ/CST	1	9	4 (44%)	0 (0%)	0 (0%)	4 (44%)						
**FEP-based**												
FEP/ATM							1	3	0 (0%)	0 (0%)	0 (0%)	0 (0%)
FEP/SUL	1	9	4 (44%)	0 (0%)	4 (44%)	0 (0%)						
FEP/SAM	1	2	2 (100%)	1 (50%)	1 (50%)	0 (0%)						
FEP/LVX							1	1	1 (100%)	0 (0%)	1 (100%)	0 (0%)
FEP/MXF							1	2	2 (100%)	0 (0%)	2 (100%)	0 (0%)
FEP/AMK							1	1	1 (100%)	0 (0%)	1 (100%)	0 (0%)
**CIP-based**												
CIP/AZM	1	3	0 (0%)	0 (0%)	0 (0%)	0 (0%)						
CIP/SUL	1	10	8 (80%)	2 (20%)	6 (70%)	0 (0%)						
CIP/MEM	2	45	4 (9%)	0 (0%)	1 (2%)	3 (7%)						

Antimicrobial combinations	CHBD			CHBD: concentration at which synergy was present			TKA			TKA: concentration at which synergy was present		
	Studies N	Isolates n	Synergy n (%)	≤ breakpoints n (%)	> breakpoints n (%)	Unclear n (%)	Studies N	Isolates n	Synergy n (%)	≤ breakpoints n (%)	> breakpoints n (%)	Unclear n (%)
CIP/DOR							1	1	0 (0%)	0 (0%)	0 (0%)	0 (0%)
CIP/AMK	1	34	4 (12%)	0 (0%)	0 (0%)	4 (12%)						
CIP/FOF	1	2	1 (50%)	0 (0%)	1 (50%)	0 (0%)	1	1	0 (0%)	0 (0%)	0 (0%)	0 (0%)
**LVX-based**												
LVX/SAM	1	7	7 (100%)	5 (71%)	2 (29%)	0 (0%)						
LVX/FEP							1	1	1 (100%)	0 (0%)	1 (100%)	0 (0%)
LVX/DOR							2	6	3 (50%)	0 (0%)	3 (50%)	0 (0%)
LVX/CZA							1	4	3 (75%)	0 (0%)	3 (75%)	0 (0%)
LVX/AMK							1	1	0 (0%)	0 (0%)	0 (0%)	0 (0%)
LVX/CST	2	2	1 (50%)	1 (50%)	0 (0%)	0 (0%)	2	2	1 (50%)	0 (0%)	1 (50%)	0 (0%)
LVX/TGC	2	25	4 (16%)	0 (0%)	4 (16%)	0 (0%)	1	2	0 (0%)	0 (0%)	0 (0%)	0 (0%)
LVX/FOF	1	1	0 (0%)	0 (0%)	0 (0%)	0 (0%)						
**MXF-based**												
MXF/CFS	1	80	4 (5%)	0 (0%)	0 (0%)	4 (5%)						
MXF/FEP							1	2	2 (100%)	0 (0%)	2 (100%)	0 (0%)
**IMP-based**												
IMP/AZM	1	2	0 (0%)	0 (0%)	0 (0%)	0 (0%)						
IMP/SUL	2	10	0 (0%)	0 (0%)	0 (0%)	0 (0%)	3	6	3 (50%)	0 (0%)	3 (50%)	0 (0%)
IMP/SAM							1	6	6 (100%)	0 (0%)	0 (0%)	6 (100%)
IMP/CFS	1	16	11 (69%)	9 (56%)	2 (13%)	0 (0%)						
IMP/MEM	1	21	6 (29%)	0 (0%)	6 (29%)	0 (0%)						
IMP/AMK	2	4	3 (75%)	0 (0%)	2 (50%)	1 (25%)						
IMP/GEN							1	2	0 (0%)	0 (0%)	0 (0%)	0 (0%)
IMP/PLZ	1	8	6 (75%)	0 (0%)	5 (63%)	1 (13%)						

Antimicrobial combinations	CHBD			CHBD: concentration at which synergy was present			TKA			TKA: concentration at which synergy was present		
	Studies N	Isolates n	Synergy n (%)	≤ breakpoints n (%)	> breakpoints n (%)	Unclear n (%)	Studies N	Isolates n	Synergy n (%)	≤ breakpoints n (%)	> breakpoints n (%)	Unclear n (%)
IMP/CST	2	10	9 (90%)	2 (20%)	7 (70%)	0 (0%)	1	2	0 (0%)	0 (0%)	0 (0%)	0 (0%)
IMP/PMB							1	1	1 (100%)	0 (0%)	1 (100%)	0 (0%)
IMP/TGC	1	12	1 (8%)	0 (0%)	1 (8%)	0 (0%)	1	1	0 (0%)	0 (0%)	0 (0%)	0 (0%)
IMP/MIN							1	1	0 (0%)	0 (0%)	0 (0%)	0 (0%)
IMP/RIF	2	28	16 (57%)	11 (39%)	3 (11%)	2 (7%)	5	13	6 (46%)	0 (0%)	6 (46%)	0 (0%)
IMP/FOF	3	45	26 (58%)	9 (20%)	10 (22%)	7 (16%)	1	9	8 (89%)	0 (0%)	8 (89%)	0 (0%)
**MEM-based**												
MEM/AZM	1	12	0 (0%)	0 (0%)	0 (0%)	0 (0%)						
MEM/ATM							1	1	0 (0%)	0 (0%)	0 (0%)	0 (0%)
MEM/SUL	6	173	72 (42%)	2 (1%)	2 (1%)	68 (39%)	3	54	32 (59%)	0 (0%)	7 (13%)	25 (46%)
MEM/SAM	2	10	2 (20%)	2 (20%)	0 (0%)	0 (0%)	1	2	0 (0%)	0 (0%)	0 (0%)	0 (0%)
MEM/CFS	1	11	0 (0%)	0 (0%)	0 (0%)	0 (0%)						
MEM/TZB	2	59	24 (41%)	0 (0%)	0 (0%)	24 (41%)						
MEM/CIP	2	45	4 (9%)	0 (0%)	1 (2%)	3 (7%)						
MEM/IMP	1	21	6 (29%)	0 (0%)	6 (29%)	0 (0%)						
MEM/CZA							2	15	3 (20%)	0 (0%)	3 (20%)	0 (0%)
MEM/AMK	4	47	16 (34%)	1 (2%)	2 (4%)	13 (28%)						
MEM/PLZ	1	4	2 (50%)	0 (0%)	2 (50%)	0 (0%)						
MEM/CST	6	29	21 (72%)	5 (17%)	11 (38%)	5 (17%)	3	4	4 (100%)	0 (0%)	4 (100%)	0 (0%)
MEM/PMB	1	3	3 (100%)	3 (100%)	0 (0%)	0 (0%)	1	2	0 (0%)	0 (0%)	0 (0%)	0 (0%)
MEM/TGC							1	1	0 (0%)	0 (0%)	0 (0%)	0 (0%)
MEM/MIN	1	3	0 (0%)	0 (0%)	0 (0%)	0 (0%)	1	3	0 (0%)	0 (0%)	0 (0%)	0 (0%)
MEM/RIF	1	50	10 (20%)	0 (0%)	0 (0%)	10 (20%)	1	2	2 (100%)	0 (0%)	2 (100%)	0 (0%)
MEM/FOF	4	79	15 (19%)	1 (1%)	14 (18%)	0 (0%)						

Antimicrobial combinations	CHBD			CHBD: concentration at which synergy was present			TKA			TKA: concentration at which synergy was present		
	Studies N	Isolates n	Synergy n (%)	≤ breakpoints n (%)	> breakpoints n (%)	Unclear n (%)	Studies N	Isolates n	Synergy n (%)	≤ breakpoints n (%)	> breakpoints n (%)	Unclear n (%)
MEM/VAN	1	5	3 (60%)	1 (20%)	0 (0%)	2 (40%)						
**DOR-based**												
DOR/SUL							1	17	4 (24%)	4 (24%)	0 (0%)	0 (0%)
DOR/SAM	1	1	0 (0%)	0 (0%)	0 (0%)	0 (0%)						
DOR/CIP							1	1	0 (0%)	0 (0%)	0 (0%)	0 (0%)
DOR/LVX							2	6	3 (50%)	0 (0%)	3 (50%)	0 (0%)
DOR/AMK	1	4	1 (25%)	0 (0%)	0 (0%)	1 (25%)	1	2	2 (100%)	0 (0%)	2 (100%)	0 (0%)
DOR/CST	3	6	2 (33%)	1 (17%)	1 (17%)	0 (0%)	5	33	23 (70%)	19 (58%)	4 (12%)	0 (0%)
DOR/TGC	1	3	3 (100%)	1 (33%)	0 (0%)	2 (67%)	1	45	5 (11%)	5 (11%)	0 (0%)	0 (0%)
DOR/RIF	1	1	0 (0%)	0 (0%)	0 (0%)	0 (0%)	1	5	2 (40%)	1 (20%)	1 (20%)	0 (0%)
DOR/FOF	1	3	3	3 (100%)	3	3 (100%)	1	1	0 (0%)	0 (0%)	0 (0%)	0 (0%)
DOR/VAN	1	3	0 (0%)	0 (0%)	0 (0%)	0 (0%)	1	3	0 (0%)	0 (0%)	0 (0%)	0 (0%)
**CZA- or AVI-based**												
AVI/SUL							1	1	1 (100%)	1 (100%)	0 (0%)	0 (0%)
CZA/LVX							1	4	3 (75%)	0 (0%)	3 (75%)	0 (0%)
CZA/MEM							2	15	3 (20%)	0 (0%)	3 (20%)	0 (0%)
CZA/TOB							1	2	1 (50%)	0 (0%)	1 (50%)	0 (0%)
CZA/CST							1	2	2 (100%)	0 (0%)	2 (100%)	0 (0%)
CZA/TGC							1	4	3 (75%)	0 (0%)	3 (75%)	0 (0%)
**AMK-based**												
AMK/AZM	1	2	0 (0%)	0 (0%)	0 (0%)	0 (0%)						
AMK/FEP							1	1	1 (100%)	0 (0%)	1 (100%)	0 (0%)
AMK/CIP	1	34	4 (12%)	0 (0%)	0 (0%)	4 (12%)						
AMK/LVX							1	1	0 (0%)	0 (0%)	0 (0%)	0 (0%)

Antimicrobial combinations	CHBD			CHBD: concentration at which synergy was present			TKA			TKA: concentration at which synergy was present		
	Studies N	Isolates n	Synergy n (%)	≤ breakpoints n (%)	> breakpoints n (%)	Unclear n (%)	Studies N	Isolates n	Synergy n (%)	≤ breakpoints n (%)	> breakpoints n (%)	Unclear n (%)
AMK/IMP	2	4	3 (75%)	0 (0%)	2 (50%)	1 (25%)						
AMK/MEM	4	47	16 (34%)	1 (2%)	2 (4%)	13 (28%)						
AMK/DOR	1	4	1 (25%)	0 (0%)	0 (0%)	1 (25%)	1	2	2 (100%)	0 (0%)	2 (100%)	0 (0%)
AMK/CST	1	6	2 (33%)	0 (0%)	0 (0%)	2 (33%)						
AMK/TGC	1	14	2 (14%)	1 (7%)	1 (7%)	0	1	1	1 (100%)	1 (100%)	0 (0%)	0 (0%)
AMK/FOF	2	29	26 (90%)	11 (38%)	15 (52%)	0 (0%)						
**GEN-based**												
GEN/SUL	1	10	8 (80%)	2 (20%)	6 (60%)	0 (0%)						
GEN/IMP							1	2	0 (0%)	0 (0%)	0 (0%)	0 (0%)
GEN/CST	1	1	0 (0%)	0 (0%)	0 (0%)	0 (0%)						
GEN/FOF	2	13	12 (92%)	3 (23%)	9 (69%)	0 (0%)						
**TOB-based**												
TOB/CZA							1	2	1 (50%)	0 (0%)	1 (50%)	0 (0%)
TOB/FOF	1	2	1 (50%)	0 (0%)	1 (50%)	0 (0%)						
**PLZ-based**												
PLZ/IMP	1	8	6 (75%)	0 (0%)	5 (63%)	1 (13%)						
PLZ/MEM	1	4	2 (50%)	0 (0%)	2 (50%)	0 (0%)						
PLZ/FOF	1	9	1 (11%)	0 (0%)	1 (11%)	0 (0%)	1	2	0 (0%)	0 (0%)	0 (0%)	0 (0%)
**CST-based**												
CST/AZM	1	8	1 (13%)	0 (0%)	0 (0%)	1(13%)						
CST/ATM	1	9	4 (44%)	0 (0%)	0 (0%)	4 (44%)						
CST/SUL							1	6	2 (33%)	2 (33%)	0 (0%)	0 (0%)
CST/SAM	2	10	0 (0%)	0 (0%)	0 (0%)	0 (0%)						
CST/CFS	1	3	0 (0%)	0 (0%)	0 (0%)	0 (0%)						

Antimicrobial combinations	CHBD			CHBD: concentration at which synergy was present			TKA			TKA: concentration at which synergy was present		
	Studies N	Isolates n	Synergy n (%)	≤ breakpoints n (%)	> breakpoints n (%)	Unclear n (%)	Studies N	Isolates n	Synergy n (%)	≤ breakpoints n (%)	> breakpoints n (%)	Unclear n (%)
CST/CAZ	1	9	4 (44%)	0 (0%)	0 (0%)	4 (44%)						
CST/LVX	2	2	1 (50%)	1 (50%)	0 (0%)	0 (0%)	2	2	1 (50%)	0 (0%)	1 (50%)	0 (0%)
CST/IMP	2	10	9 (90%)	2 (20%)	7 (70%)	0 (0%)	1	2	0 (0%)	0 (0%)	0 (0%)	0 (0%)
CST/MEM	6	29	21 (72%)	5 (17%)	11 (38%)	5 (17%)	3	4	4 (100%)	0 (0%)	4 (100%)	0 (0%)
CST/DOR	3	6	2 (33%)	1 (17%)	1 (17%)	0 (0%)	5	33	23 (70%)	19 (58%)	4 (12%)	0 (0%)
CST/CZA							1	2	2 (100%)	0 (0%)	2 (100%)	0 (0%)
CST/AMK	1	6	2 (33%)	0 (0%)	0 (0%)	2 (33%)						
CST/GEN	1	1	0 (0%)	0 (0%)	0 (0%)	0 (0%)						
CST/TGC	2	10	0 (0%)	0 (0%)	0 (0%)	0 (0%)	1	12	7 (58%)	7 (58%)	0 (0%)	0 (0%)
CST/ERV	1	3	1 (33%)	0 (0%)	1 (33%)	0 (0%)						
CST/RIF	5	40	31 (78%)	10 (25%)	10 (25%)	11 (28%)	3	7	7 (100%)	0 (0%)	6 (86%)	1 (14%)
CST/RFB	1	3	3 (100%)	0 (0%)	0 (0%)	3 (100%)						
CST/FOF	2	9	0 (0%)	0 (0%)	0 (0%)	0 (0%)						
CST/TMP	1	1	1 (100%)	0 (0%)	1 (100%)	0 (0%)	1	1	1 (100%)	0 (0%)	1 (100%)	0 (0%)
CST/SXT	2	8	2 (25%)	1 (13%)	0 (0%)	1 (13%)	1	1	1 (100%)	0 (0%)	1 (100%)	0 (0%)
CST/CHL	1	2	2 (100%)	1 (50%)	1 (50%)	0 (0%	1	2	2 (100%)	0 (0%)	2 (100%)	0 (0%)
CST/FA	2	6	6 (100%)	1 (17%)	2 (33%)	3 (50%)	2	4	3 (75%)	0 (0%)	3 (75%)	0 (0%)
CST/VAN	7	33	29 (88%)	2 (6%)	2 (6%)	25 (76%)	6	20	16 (80%)	13 (65%)	3 (15%)	0 (0%)
CST/TEC	1	9	4 (44%)	0 (0%)	0 (0%)	4 (44%)						
CST/DAP							2	5	0 (0%)	0 (0%)	0 (0%)	0 (0%)
**PMB-based**												
PMB/SUL	1	3	2 (67%)	2 (67%)	0 (0%)	0 (0%)						
PMB/SAM							1	2	0 (0%)	0 (0%)	0 (0%)	0 (0%)
PMB/IMP							1	1	1 (100%)	0 (0%)	1 (100%)	0 (0%)

Antimicrobial combinations	CHBD			CHBD: concentration at which synergy was present			TKA			TKA: concentration at which synergy was present		
	Studies N	Isolates n	Synergy n (%)	≤ breakpoints n (%)	> breakpoints n (%)	Unclear n (%)	Studies N	Isolates n	Synergy n (%)	≤ breakpoints n (%)	> breakpoints n (%)	Unclear n (%)
PMB/MEM	1	3	3 (100%)	3 (100%)	0 (0%)	0 (0%)	1	2	0 (0%)	0 (0%)	0 (0%)	0 (0%)
PMB/TGC	1	4	3 (75%)	0 (0%)	2 (50%)	1 (25%)	1	4	0 (0%)	0 (0%)	0 (0%)	0 (0%)
PMB/RIF	1	3	3 (100%)	1 (33%)	1 (33%)	1 (33%)	1	3	1 (33%)	1 (33%)	0 (0%)	0 (0%)
PMB/FOF	1	3	0 (0%)	0 (0%)	0 (0%)	0 (0%)						
PMB/VAN	1	3	3 (100%)	3 (100%)	0 (0%)	0 (0%)	1	3	2 (67%)	0 (0%)	2 (67%)	0 (0%)
**TGC-based**												
TGC/SAM	2	13	0 (0%)	0 (0%)	0 (0%)	0 (0%)						
TGC/TZP	1	18	0 (0%)	0 (0%)	0 (0%)	0 (0%)						
TGC/LVX	2	25	4 (16%)	0 (0%)	4 (16%)	0 (0%)	1	2	0 (0%)	0 (0%)	0 (0%)	0 (0%)
TGC/IMP	1	12	1 (8%)	0 (0%)	1 (8%)	0 (0%)	1	1	0 (0%)	0 (0%)	0 (0%)	0 (0%)
TGC/MEM							1	1	0 (0%)	0 (0%)	0 (0%)	0 (0%)
TGC/DOR	1	3	3 (100%)	1 (33%)	0 (0%)	2 (67%)	1	45	5 (11%)	5 (11%)	0 (0%)	0 (0%)
TGC/CZA							1	4	3 (75%)	0 (0%)	3 (75%)	0 (0%)
TGC/AMK	1	14	2 (14%)	1 (7%)	1 (7%)	0 (0%)	1	1	1 (100%)	1 (100%)	0 (0%)	0 (0%)
TGC/CST	2	10	0 (0%)	0 (0%)	0 (0%)	0 (0%)	1	12	7 (58%)	7 (58%)	0 (0%)	0 (0%)
TGC/PMB	1	4	3 (75%)	0 (0%)	2 (50%)	1 (25%)	1	4	0 (0%)	0 (0%)	0 (0%)	0 (0%)
TGC/RIF	2	16	1 (6%)	0 (0%)	1 (6%)	0 (0%)	2	4	1 (25%)	1 (25%)	0 (0%)	0 (0%)
TGC/FOF	1	4	3 (75%)	1 (25%)	2 (50%)	0 (0%)	1	1	1 (100%)	0 (0%)	1 (100%)	0 (0%)
**MIN-based**												
MIN/CFS	1	2	0 (0%)	0 (0%)	0 (0%)	0 (0%)	1	2	1 (50%)	0 (0%)	1 (50%)	0 (0%)
MIN/IMP							1	1	0 (0%)	0 (0%)	0 (0%)	0 (0%)
MIN/MEM	1	3	0 (0%)	0 (0%)	0 (0%)	0 (0%)	1	3	0 (0%)	0 (0%)	0 (0%)	0 (0%)
**ERV-based**												
ERV/CST	1	3	1 (33%)	0 (0%)	1 (33%)	0 (0%)						

Antimicrobial combinations	CHBD			CHBD: concentration at which synergy was present			TKA			TKA: concentration at which synergy was present		
	Studies N	Isolates n	Synergy n (%)	≤ breakpoints n (%)	> breakpoints n (%)	Unclear n (%)	Studies N	Isolates n	Synergy n (%)	≤ breakpoints n (%)	> breakpoints n (%)	Unclear n (%)
**RIF-based**												
RIF/SUL	1	50	10 (20%)	0 (0%)	0 (0%)	10 (20%)	2	6	3 (50%)	0 (0%)	3 (50%)	0 (0%)
RIF/SAM	1	7	7 (100%)	4 (57%)	1 (14%)	2 (29%)						
RIF/CFS	1	7	2 (29%)	1 (14%)	0 (0%)	1 (14%)						
RIF/IMP	2	28	16 (57%)	11 (39%)	3 (11%)	2 (7%)	5	13	6 (46%)	0 (0%)	6 (46%)	0 (0%)
RIF/MEM	1	50	10 (20%)	0 (0%)	0 (0%)	10 (20%)	1	2	2 (100%)	0 (0%)	2 (100%)	0 (0%)
RIF/DOR	1	1	0 (0%)	0 (0%)	0 (0%)	0 (0%)	1	5	2 (40%)	0 (0%)	1 (20%)	0 (0%)
RIF/CST	5	40	31 (78%)	10 (25%)	10 (25%)	11 (28%)	3	7	7 (100%)	0 (0%)	6 (86%)	1 (14%)
RIF/PMB	1	3	3 (100%)	1 (33%)	1 (33%)	1 (33%)	1	3	1 (33%)	1 (33%)	0 (0%)	0 (0%)
RIF/TGC	2	16	1 (6%)	0 (0%)	1 (6%)	0 (0%)	2	4	1 (25%)	1 (25%)	0 (0%)	0 (0%)
RIF/FOF	1	50	12 (24%)	0 (0%)	0 (0%)	12 (24%)						
RIF/LZD	1	3	0 (0%)	0 (0%)	0 (0%)	0 (0%)						
**RFB-based**												
RFB/CST	1	3	3 (100%	0 (0%)	0 (0%)	3 (100%						
**FOF-based**												
FOF/SUL	2	56	41 (73%)	3 (5%)	37 (66%)	1 (2%)	2	10	7 (70%)	0 (0%)	7 (70%)	0 (0%)0
FOF/CIP	1	2	1 (50%)	0 (0%)	1 (50%)	0 (0%)	1	1	0 (0%)	0 (0%)	0 (0%)	0 (0%)
FOF/LVX	1	1	0 (0%)	0 (0%)	0 (0%)	0 (0%)						
FOF/IMP	3	45	26 (58%)	9 (20%)	10 (22%)	7 (16%)	1	9	8 (89%)	0 (0%)	8 (89%)	0 (0%)
FOF/MEM	4	79	15 (19%)	1 (1%)	14 (18%)	0 (0%)						
FOF/DOR	1	3	3 (100%)	0 (0%)	3 (100%)	0 (0%)	1	1	0 (0%)	0 (0%)	0 (0%)	0 (0%)
FOF/AMK	2	29	26 (90%)	11 (38%)	15 (52%)	0 (0%)						
FOF/GEN	2	13	12 (92%)	3 (23%)	9 (69%)	0 (0%)						
FOF/TOB	1	2	1 (50%)	0 (0%)	1 (50%)	0 (0%)						

Antimicrobial combinations	CHBD			CHBD: concentration at which synergy was present			TKA			TKA: concentration at which synergy was present		
	Studies N	Isolates n	Synergy n (%)	≤ breakpoints n (%)	> breakpoints n (%)	Unclear n (%)	Studies N	Isolates n	Synergy n (%)	≤ breakpoints n (%)	> breakpoints n (%)	Unclear n (%)
FOF/PLZ	1	9	1 (11%)	0 (0%)	1 (11%)	0 (0%)	1	2	0 (0%)	0 (0%)	0 (0%)	0 (0%)
FOF/CST	2	9	0 (0%)	0 (0%)	0 (0%)	0 (0%)						
FOF/PMB	1	3	0 (0%)	0 (0%)	0 (0%)	0 (0%)						
FOF/TGC	1	4	3 (75%)	1 (25%)	2 (50%)	0 (0%)	1	1	1 (100%)	0 (0%)	1 (100%)	0 (0%)
FOF/RIF	1	50	12 (24%)	0 (0%)	0 (0%)	12 (24%)						
**TMP-based**												
TMP/CST	1	1	1 (100%)	1 (100%)	0 (0%)	0 (0%)	1	1	1 (100%)	0 (0%)	1 (100%)	0 (0%)
**SXT-based**												
SXT/CST	2	8	2 (25%)	1 (13%)	0 (0%)	1 (13%)	1	1	1 (100%)	0 (0%)	1 (100%)	0 (0%)
**CHL-based**												
CHL/CST	1	2	2 (100%)	1 (50%)	1 (50%)	0 (0%)	1	2	2 (100%)	0 (0%)	2 (100%)	0 (0%)
**FA-based**												
FA/CST	2	6	6 (100%)	1 (17%)	2 (33%)	3 (50%)	2	4	3 (75%)	0 (0%)	3 (75%)	0 (0%)
**VAN-based**												
VAN/CAZ												
VAN/MEM	1	5	3 (60%)	1 (20%)	0 (0%)	2 (40%)						
VAN/DOR	1	3	0 (0%)	0 (0%)	0 (0%)	0 (0%)	1	3	0 (0%)	0 (0%)	0 (0%)	0 (0%)
VAN/CST	7	33	29 (88%)	2 (6%)	2 (6%)	25 (76%)	6	20	16 (80%)	13 (65%0	3 (15%)	0 (0%)
VAN/PMB	1	3	3 (100%)	3 (100%)	0 (0%)	0 (0%)	1	3	2 (67%)	0 (0%)	2 (67%)	0 (0%)
**TEC-based**												
TEC/CST	1	9	4 (44%)	0 (0%)	0 (0%)	4 (44%)						
**DAP-based**												
DAP/CST							2	5	0 (0%)	0 (0%)	0 (0%)	0 (0%)
**LZD-based**												

Antimicrobial combinations	CHBD			CHBD: concentration at which synergy was present			TKA			TKA: concentration at which synergy was present		
	Studies N	Isolates n	Synergy n (%)	≤ breakpoints n (%)	> breakpoints n (%)	Unclear n (%)	Studies N	Isolates n	Synergy n (%)	≤ breakpoints n (%)	> breakpoints n (%)	Unclear n (%)
LZD/RIF	1	3	0 (0%)	0 (0%)	0 (0%)	0 (0%)						
**Triple combinations**												
PMB/FOF/MEM	1	3	3 (100%)	3 (100%)	0 (0%)	0 (0%)						
PMB/SUL/MEM	1	3	3 (100%)	3 (100%)	0 (0%)	0 (0%)						
PMB/SAM/MEM							2	2	1 (50%)	0 (0%)	1 (50%)	0 (0%)
CST/DOR/SUL							1	6	6 (100%)	6 (100%)	0 (0%)	0 (0%)
CST/VAN/DOR							1	3	3 (100%)	3 (100%)	0 (0%)	0 (0%)
IMP/SUL/AMK							1	8	0 (0%)	0 (0%)	0 (0%)	0 (0%)
IMP/TIM/AMK							1	8	0 (0%)	0 (0%)	0 (0%)	0 (0%)
TZP/SUL/AMK							1	8	0 (0%)	0 (0%)	0 (0%)	0 (0%)
TZP/TIM/AMK							1	8	0 (0%)	0 (0%)	0 (0%)	0 (0%)
**Sum**	139	1402	539 (39%)	112 (8%)	194 (14%)	233 (17%)	99	407	185 (46%)	65 (16%)	88 (22%)	32 (8%)

%= 100*n/N

MCBT assay was used in only 1 study and is not shown in the Table. See Section 4.5 (Bae S, 2016 [32])

Highlighted in green= combinations shown to be synergistic at concentrations equal to or lower than established breakpoints of resistance.

4.2 Summary Table (sum of all studies): Proportion of synergy and clinically relevant synergy in gradient- and disk-based methods

Antimicrobial combinations	Gradient methods*			Gradient methods*: concentration at which synergy was present			Disk methods*			Disk methods*: concentration at which synergy was present		
	Studies N	Isolates n	Synergy n (%)	≤ breakpoints n (%)	> breakpoints n (%)	Unclear n (%)	Studies N	Isolates n	Synergy n (%)	≤ breakpoints n (%)	> breakpoints n (%)	Unclear n (%)
**SUL-based**												
SUL/MEM	2	61	23 (38%)	0 (0%)	0 (0%)	23 (38%)						
**SAM-based**												
SAM/RIF	1	7	0 (0%)	0 (0%)	0 (0%)	0 (0%)						
**CSF-based**												
CFS/MXF	1	80	2 (3%)	0 (0%)	0 (0%)	2 (3%)						
CFS/IMP	1	19	9 (47%)	0 (0%)	0 (0%)	9 (47%)						
CFS/MEM	1	19	10 (53%)	0 (0%)	0 (0%)	10 (53%)						
CFS/DOR	1	19	4 (21%)	0 (0%)	0 (0%)	4 (21%)						
CFS/RIF	1	7	0 (0%)	0 (0%)	0 (0%)	0 (0%)						
**TZB-based**												
TZB/MEM	1	54	19 (35%)	0 (0%)	0 (0%)	19 (35%)						
**MXF-based**												
MXF/CFS	1	80	2 (3%)	0 (0%)	0 (0%)	2 (3%)						
MXF/IMP	1	21	1 (5%)	0 (0%)	0 (0%)	1 (5%)						
MXF/MEM	1	21	0 (0%)	0 (0%)	0 (0%)	0 (0%)						
MXF/DOR	1	21	0 (0%)	0 (0%)	0 (0%)	0 (0%)						
**IMP-based**												
IMP/CFS	1	19	9 (47%)	0 (0%)	0 (0%)	9 (47%)						
IMP/MXF	1	21	1 (5%)	0 (0%)	0 (0%)	1 (5%)						

Antimicrobial combinations	Gradient methods*			Gradient methods*: concentration at which synergy was present			Disk methods*			Disk methods*: concentration at which synergy was present		
	Studies N	Isolates n	Synergy n (%)	≤ breakpoints n (%)	> breakpoints n (%)	Unclear n (%)	Studies N	Isolates n	Synergy n (%)	≤ breakpoints n (%)	> breakpoints n (%)	Unclear n (%)
IMP/NET	1	21	0 (0%)	0 (0%)	0 (0%)	0 (0%)						
IMP/CST	1	41	19 (46%)	0 (0%)	0 (0%)	19 (46%)						
IMP/DOX	1	21	0 (0%)	0 (0%)	0 (0%)	0 (0%)						
IMP/RIF	1	17	0 (0%)	0 (0%)	0 (0%)	0 (0%)	1	1	1 (100%)	0 (0%)	1(100%)	0 (0%)
**MEM-based**												
MEM/SUL	2	61	23 (38%)	0 (0%)	0 (0%)	23 (38%)						
MEM/CFS	1	19	10 (53%)	0 (0%)	0 (0%)	10 (53%)						
MEM/TZB	1	54	19 (35%)	0 (0%)	0 (0%)	19 (35%)						
MEM/MXF	1	21	0 (0%)	0 (0%)	0 (0%)	0 (0%)						
MEM/CZA							1	11	2 (18%)	0 (0%)	0 (0%)	2 (18%)
MEM/NET	1	21	0 (0%)	0 (0%)	0 (0%)	0 (0%)						
MEM/CST	1	41	35 (85%)	2 (5%)	0 (0%)	33 (81%)						
MEM/DOX	1	21	0 (0%)	0 (0%)	0 (0%)	0 (0%)						
MEM/RIF	1	17	0 (0%)	0 (0%)	0 (0%)	0 (0%)						
**DOR-based**												
DOR/CFS	1	19	4 (21%)	0 (0%)	0 (0%)	4 (21%)						
DOR/MXF	1	21	0 (0%)	0 (0%)	0 (0%)	0 (0%)						
DOR/NET	1	21	0 (0%)	0 (0%)	0 (0%)	0 (0%)						
DOR/DOX	1	21	0 (0%)	0 (0%)	0 (0%)	0 (0%)						
DOR/RIF	1	17	0 (0%)	0 (0%)	0 (0%)	0 (0%)						
**CZA- or AVI-based**												
CZA/MEM							1	11	2 (18%)	0 (0%)	0 (0%)	2 (18%)
**NET-based**												
NET/IMP	1	21	0 (0%)	0 (0%)	0 (0%)	0 (0%)						

Antimicrobial combinations	Gradient methods*			Gradient methods*: concentration at which synergy was present			Disk methods*			Disk methods*: concentration at which synergy was present		
	**Studies N**	**Isolates n**	**Synergy n (%)**	**≤ breakpoints n (%)**	**> breakpoints n (%)**	**Unclear n (%)**	**Studies N**	**Isolates n**	**Synergy n (%)**	**≤ breakpoints n (%)**	**> breakpoints n (%)**	**Unclear n (%)**
NET/MEM	1	21	0 (0%)	0 (0%)	0 (0%)	0 (0%)						
NET/DOR	1	21	0 (0%)	0 (0%)	0 (0%)	0 (0%)						
**CST-based**												
CST/IMP	1	41	19 (46%)	0 (0%)	0 (0%)	19 (46%)						
CST/MEM	1	41	35 (85%)	2 (5%)	0 (0%)	33 (81%)						
CST/RIF	2	45	37 (82%)	0 (0%)	0 (0%)	37 (82%)						
CST/FA							1	3	3 (100%)	0 (0%)	3 (100%)	0 (0%)
CST/DAP	1	4	0 (0%)	0 (0%)	0 (0%)	0 (0%)						
**PMB-based**												
PMB/TGC	1	4	0 (0%)	0 (0%)	0 (0%)	0 (0%)						
PMB/RIF	1	3	1 (33%)	0 (0%)	0 (0%)	1 (33%)						
PMB/VAN	1	3	3 (100%)	3 (100%)	0 (0%)	0 (0%)	1	3	3 (100%)	3 (100%)	0 (0%)	0 (0%)
**DOX-based**												
DOX/IMP	1	21	0 (0%)	0 (0%)	0 (0%)	0 (0%)						
DOX/MEM	1	21	0 (0%)	0 (0%)	0 (0%)	0 (0%)						
DOX/DOR	1	21	0 (0%)	0 (0%)	0 (0%)	0 (0%)						
**TGC-based**												
TGC/PMB	1	4	0 (0%)	0 (0%)	0 (0%)	0 (0%)						
TGC/RIF	1	3	1 (33%)	0 (0%)	0 (0%)	1 (33%)						
**RIF-based**												
RIF/SAM	1	7	0 (0%)	0 (0%)	0 (0%)	0 (0%)						
RIF/CFS	1	7	0 (0%)	0 (0%)	0 (0%)	0 (0%)						
RIF/IMP	1	17	0 (0%)	0 (0%)	0 (0%)	0 (0%)	1	1	1 (100%)	0 (0%)	1 (100%)	0 (0%)
RIF/MEM	1	17	0 (0%)	0 (0%)	0 (0%)	0 (0%)						
RIF/DOR	1	17	0 (0%)	0 (0%)	0 (0%)	0 (0%)						

Antimicrobial combinations	Gradient methods*			Gradient methods*: concentration at which synergy was present			Disk methods*			Disk methods*: concentration at which synergy was present		
	Studies N	Isolates n	Synergy n (%)	≤ breakpoints n (%)	> breakpoints n (%)	Unclear n (%)	Studies N	Isolates n	Synergy n (%)	≤ breakpoints n (%)	> breakpoints n (%)	Unclear n (%)
RIF/CST	2	45	37 (82%)	0 (0%)	0 (0%)	37 (82%)						
RIF/PMB	1	3	1 (33%)	0 (0%)	0 (0%)	1 (33%)						
RIF/TGC	1	3	1 (33%)	0 (0%)	0 (0%)	1 (33%)						
**FA-based**												
FA/CST							1	3	3 (100%)	0 (0%)	3 (100%)	0 (0%)
**VAN-based**												
VAN/PMB	1	3	3 (100%)	3 (100%)	0 (0%)	0 (0%)	1	3	3 (100%)	3 (100%)	0 (0%)	0 (0%)
**DAP-based**												
DAP/CST	1	4	0 (0%)	0 (0%)	0 (0%)	0 (0%)						
**Sum**	30	650	164 (25%)	5 (1%)	0 (0%)	159 (24%)	4	18	9 (50%)	3 (17%)	4 (22%)	2 (11%)

%= 100*n/N

* Including gradient/agar and disk/agar methods

Highlighted in green= combinations shown to be synergistic at concentrations equal to or lower than established breakpoints of resistance.

4.3 Summary Table (sum of all studies): Proportion of synergy and clinically relevant synergy in agar dilution and dynamic in vitro PK/PD models

Antimicrobial combinations	Agar dilution			Agar dilution: concentration at which synergy was present			Dynamic in vitro PK/PD studies		
	Studies N	Isolates n	Synergy n (%)	≤ breakpoints n (%)	> breakpoints n (%)	Unclear n (%)	Studies N	Isolates n	Synergy n (%)
**SUL-based**									
SUL/IMP	1	4	4 (100%)	4 (100%)	0 (0%)	0 (0%)			
SUL/MEM	1	4	4 (100%)	4 (100%)	0 (0%)	0 (0%)			
SUL/AVI	1	38	35 (92%)	35 (92%)	0 (0%)	0 (0%)			
**SAM-based**									
SAM/MEM							1	1	0 (0%)
SAM/DOR							1	3	0 (0%)
SAM/PMB							1	1	0 (0%)
SAM/TGC							1	1	0 (0%)
**FEP-based**									
FEP/AMK							1	1	0 (0%)
**LVX-based**									
LVX/AMK							1	1	0 (0%)
**ETP-based**									
ETP/IMP							1	3	0 (0%)
**IMP-based**									
IMP/SUL	1	4	4 (100%)	4 (100%)	0 (0%)	0 (0%)			
IMP/ETP							1	3	0 (0%)
IMP/CST							1	1	0 (0%)
**MEM-based**									
MEM/SUL	1	4	4 (100%)	4 (100%)	0 (0%)	0 (0%)			

Antimicrobial combinations	Agar dilution			Agar dilution: concentration at which synergy was present			Dynamic in vitro PK/PD studies		
	**Studies N**	**Isolates n**	**Synergy n (%)**	**≤ breakpoints n (%)**	**> breakpoints n (%)**	**Unclear n (%)**	**Studies N**	**Isolates n**	**Synergy n (%)**
MEM/SAM							1	1	0 (0%)
MEM/PMB							1	1	0 (0%)
**DOR-based**									
DOR/SAM							1	3	0 (0%)
DOR/TGC							1	2	0 (0%)
**CZA- or AVI-based**									
AVI/SUL	1	38	35 (92%)	35 (92%)	0 (0%)	0 (0%)			
**AMK-based**									
AMK/FEP							1	1	0 (0%)
AMK/LVX							1	1	0 (0%)
**CST-based**									
CST/IMP							1	1	0 (0%)
CST/RIF							1	1	1 (100%)
CST/DAP							1	1	0 (0%)
**PMB-based**									
PMB/SAM							1	1	0 (0%)
PMB/MEM							1	1	0 (0%)
**TGC-based**									
TGC/SAM							1	1	0 (0%)
TGC/DOR							1	2	0 (0%)
**RIF-based**									
RIF/CST							1	1	1 (100%)
**DAP-based**									
DAP/CST							1	1	0 (0%)

Antimicrobial combinations	Agar dilution			Agar dilution: concentration at which synergy was present			Dynamic in vitro PK/PD studies		
	Studies N	Isolates n	Synergy n (%)	≤ breakpoints n (%)	> breakpoints n (%)	Unclear n (%)	Studies N	Isolates n	Synergy n (%)
**Triple combinations**									
PMB/SAM/MEM							1	1	1 (100%)
**Sum**	3	46	43 (94%)	43 (94%)	0 (0%)	0 (0%)	13	18	2 (11%)

%= 100*n/N

Highlighted in green= combinations shown to be synergistic at concentrations equal to or lower than established breakpoints of resistance, or active in dynamic in vitro PK/PD models.

4.4 Proportion of synergy and assessment of clinical relevance of observed synergy in checkerboard assays (study level data)

Study-combinations	Synergy % (n/N)	Synergy at concentrations ≤ breakpoints % (n/N)	Synergy at concentrations > breakpoints % (n/N)	Synergy but unclear if at concentration ≤ breakpoints % (n/N)	Notes
**Cebrero-Cangueiro T, 2021 [1] and Nordmann P, 2020 [2]**					
MEM/IMP	29 (6/21)	0 (0/21)	29 (6/21)	0 (0/21)	Checkerboard: Based on reported FICIs synergy was present at the following MEM/IMP concentrations: 32/16, 16/8, 16/8, 32/32, 16/8, 16/4 mg/L Animal model: Similar mortality and bacterial clearance comparing meropenem monotherapy to combination therapy. Decreased bacterial loads with combination vs monotherapy.
**Cheng J 2021 [3]**					
CST/RFB	100 (3/3)	0 (0/3)	0 (0/3)	100 (3/3)	Concentrations at which synergy was observed are not reported
CST/RIF	100 (2/2)	0 (0/2)	0 (0/2)	100 (2/2)	
**Nwabor OF 2021 [4]**					
FOF/IMP	0 (0/2)	NA	NA	NA	No synergy
FOF/MEM	0 (0/1)	NA	NA	NA	No synergy
FOF/DOR	100 (3/3)	0 (0/3)	100 (3/3)	0 (0/3)	Synergy at: 128/2, 128/4 and 512/4 mg/L.
FOF/TOB	50 (1/2)	0 (0/2)	50 (1/2)	0 (0/2)	Synergy at 128/2 mg/L.
FOF/GEN	100 (2/2)	0 (0/2)	100 (2/2)	0 (0/2)	Synergy at 128/8 and 128/64 mg/L.
FOF/CIP	50 (1/2)	0 (0/2)	50 (1/2)	0 (0/2)	Synergy at 128/2 or 64/8 mg/L (2 possible MIC combinations based on the reported FICI).

Study-combinations	Synergy % (n/N)	Synergy at concentrations ≤ breakpoints % (n/N)	Synergy at concentrations > breakpoints % (n/N)	Synergy but unclear if at concentration ≤ breakpoints % (n/N)	Notes
FOF/LVX	0 (0/1)	NA	NA	NA	No synergy
FOF/TGC	75 (3/4)	25 (1/3)	50 (2/3)	0 (0/3)	Synergy at 128/0.5, 32/1 and 128/0.5 mg/L).
**Armengol E, 2020 [5]**					
RIF/LZD	0 (0/3)	NA	NA	NA	No synergy
**Li J, 2020 [6]**					
CST/MEM	80 (4/5)	40 (2/5)	20 (1/5)	20 (1/5)	Based on reported MICs and FICIs synergy at: AB075: 1/8 or 0.5/16 mg/L, AB084: 0.58 mg/L, AB118: 1/2 or 0.5/4 mg/L, AB133: 1/36 mg/L.
CST/LVX	100 (1/1)	100 (1/1)	0 (0/1)	0 (0/1)	Based on reported MICs and FICIs synergy at: AB075: 1/2 or 0.5/4 mg/L.
**Limsrivanichakorn S, 2020 [7]**					
CFS/MXF	5 (4/80)	0 (0/80)	0 (0/80)	5 (4/80)	Unclear at which concentration synergy was present
**Mohd Sazlly Lim S 2020 [8], 2021 [9], 2021 [10,85]**					
SUL/FOF	74 (37/50)	2 (1/50)	72 (36/50)	0 (0/50)	Synergy at: 0.5/256 (n=1), 4/128 (n=1), 8/32 (n=3), 8/64 (n=1), 8/128 (n=3), 16/32 (n=7), 16/64 (n=6), 16/128 (n=1), 32/8 (n=3), 32/32 (n=2), 32/64 (n=6), 32/128 (n=1), 64/8 (n=1), 64/64 (n= 1) mg/L.
SUL/MEM	56 (28/50)	0 (0/50)	56 (28/50)	0 (0/50)	Synergy at: 4/16 (n=1), 16/8 (n=2), 16/16 (n=1), 32/1 (n=1), 32/4 (n=1), 32/8 (n=2), 32/16 (n=4), 32/32 (n=5), 32/128 (n=1), 64/1 (n=2), 64/4 (n=1), 64/8 (n=2), 64/16

Study-combinations	Synergy % (n/N)	Synergy at concentrations ≤ breakpoints % (n/N)	Synergy at concentrations > breakpoints % (n/N)	Synergy but unclear if at concentration ≤ breakpoints % (n/N)	Notes
					(n=2), 64/32 (n=2), 64/128 (n=1).
FOF/MEM	28 (14/50)	0 (0/50)	28 (14/50)	0 (0/50)	Synergy at: 8/128 (n=2), 32/16 (n=2), 64/4 (n=1), 64/8 (n=1), 128/1 (n=1), 128/8 (n=2), 128/16 (n=1), 256/4 (n=1), 256/8 (n=2), 512/1 (n=1) mg/L.
FOF/RIF	24 (12/50)	0 (0/50)	0 (0/50)	24 (12/50)	Data not available. Combination MIC50 and MIC90 64/2 and 256/8 mg/L
MEM/RIF	20 (10/50)	0 (0/50)	0 (0/50)	20 (10/50)	Data not available. Combination MIC50 and MIC90 32/2 and 64/4mg/L
RIF/SUL	20 (10/50)	0 (0/50)	0 (0/50)	20 (10/50)	Data not available. Combination MIC50 and MIC90 2/32 and 4/64 mg/L
**Ghaith D, 2019 [13]**					
CST/RIF	61 (14/23)	22 (5/23)	39 (9/23)	0 (0/23)	Synergy at the following concentrations: 4/2, 16/1, 1/0.5, 0.5/0.5, 16/1, 0.5/0.5, 0.5/0.5, 2/16, 8/2, 2/0.5, 0.5/16, 0.5/16, 0.5/8, and 4/2 mg/L
**Mengucci TC 2019 [15], 2016 [16]**					
PMB/MEM	100 (3/3)	100 (3/3)	0 (0/3)	0 (0/3)	Synergy at; 0.5/0.5, 0.5/0.5 and 0.5/4 mg/L
PMB/SUL	67 (2/3)	67 (2/3)	0 (0/3)	0 (0/3)	Synergy at; 2/2 and 0.5/4 mg/L
PMB/FOF	0 (0/3)	NA	NA	NA	No synergy
FOF/MEM	17 (1/6)	17 (1/6)	0 (0/6)	0 (0/6)	Synergy at 32/2 mg/L
SUL/MEM	67 (4/6)	33 (2/6)	33 (2/6)	0 (0/6)	Synergy at 8/16, 4/8, 8/32 and 2/8 mg/L
PMB/FOF/MEM	100 (3/3)	100 (3/3)	0 (0/3)	0 (0/3)	Synergy at; 0.125/0.25/2, 0.125/4/8 and 0.125/1/16 mg/L
PMB/SUL/MEM	100 (3/3)	100 (3/3)	0 (0/3)	0 (0/3)	Synergy at; 0.5/2/2, 0.5/2/8 and 0.5/2/4 mg/L

Study-combinations	Synergy % (n/N)	Synergy at concentrations ≤ breakpoints % (n/N)	Synergy at concentrations > breakpoints % (n/N)	Synergy but unclear if at concentration ≤ breakpoints % (n/N)	Notes
**Oliva A, 2019 [17]**					
CST/MEM	100 (1/1)	100 (1/1)	0 (0/1)	0 (0/1)	Synergy at 0.5/16mg/L
CST/RIF	100 (2/2)	50 (1/2)	50 (1/2)	0 (0/2)	Synergy at 0.5/0.5 and 0.5/64mg/L.
CST/VAN	50 (1/2)	50 (1/2)	50 (1/2)	0 (0/2)	Synergy at 0.5/8 mg/L
**Ozger HS, 2019 [18]**					
CST/ERV	33 (1/3)	0 (0/3)	33 (1/3)	0 (0/3)	Synergy at 32/1 mg/L
**Phee LM, 2015 [19] and 2019 [20]**					
CST/FA	100 (3/3)	33 (1/3)	67 (1/3)	0 (0/3)	Synergy against all 3 isolates: against 2 of the 3 isolates at CST concentration >2mg/l (growth inhibition probability was <20% in PK/PD modelling against 1 of these isolates) and at 0.0625/4 mg/L against the other isolate.
**Shinohara DR, 2019 [22]**					
PMB/VAN	100 (3/3)	100 (3/3)	0 (3/3)	0 (3/3)	Synergy at 0.5/16, 4/2 and 0.5/8 mg/L
**Wang J, 2019 [23]**					
MEM/VAN	60 (3/5)	20 (1/5)	0 (0/5)	40 (2/5)	Synergy present against 3 of 5 isolates, against 1 isolate at concentrations ≤ breakpoints, while against the other 2 isolates it was not possible to confirm whether synergy was present at concentrations ≤ breakpoints.
SAM/MEM	40 (2/5)	40 (2/5)	0 (0/5)	0 (0/5)	Based on reported FICIs and MICs synergy was

Study-combinations	Synergy % (n/N)	Synergy at concentrations ≤ breakpoints % (n/N)	Synergy at concentrations > breakpoints % (n/N)	Synergy but unclear if at concentration ≤ breakpoints % (n/N)	Notes
					present at concentrations below breakpoints (exact concentrations not available).
MEM/TZB	40 (2/5)	0 (0/5)	0 (0/5)	40 (2/5)	Not possible to extract whether synergy was present at concentrations ≤ breakpoints.
CST/MEM	80 (4/5)	40 (2/5)	0 (0/5)	40 (2/5)	Synergy in checkerboard against 4 of 5 isolates, against 2 isolates at concentrations ≤ breakpoints, while against the other 2 isolates it was not possible to confirm whether synergy was present at concentrations ≤ breakpoints.
**Chen F, 2018 [24]**					
MEM/AMK	35 (12/34)	0 (0/34)	0 (0/34)	35 (12/34)	Concentrations at which synergy was present are not reported
MEM/CIP	9 (3/34)	0 (0/34)	0 (0/34)	9 (3/34)	
AMK/CIP	12 (4/34)	0 (0/34)	0 (0/34)	12 (4/34)	
**Singham-In U, 2018 [25]**					
MEM/FOF	0 (0/22)	NA	NA	NA	No synergy
MEM/AMK	100 (2/2)	50 (1/2)	0 (0/2)	50 (1/2)	Synergy against both isolates, against one at concentrations ≤ breakpoints, against the other unclear if ≤ breakpoints
IMP/FOF	65 (15/23)	0 (0/23)	0 (0/23)	65 (15/23)	Concentrations at which synergy was present are not reported.
IMP/AMK	50 (1/2)	0 (0/2)	0 (0/2)	50 (1/2)	Unclear if synergy was present at concetrations ≤ breakpoints
**Zhu W, 2018 [26]**					

Study-combinations	Synergy % (n/N)	Synergy at concentrations ≤ breakpoints % (n/N)	Synergy at concentrations > breakpoints % (n/N)	Synergy but unclear if at concentration ≤ breakpoints % (n/N)	Notes
CST/IMP	67 (2/3)	67 (2/3)	0 (0/2)	0 (0/2)	Based on FICIs <0.5 and reported MICs synergy was present at <1/4 mg/L
CST/DOR	0 (0/2)	NA	NA	NA	No synergy
CST/FOF	0 (0/3)	NA	NA	NA	No synergy
CST/CFS	0 (0/3)	NA	NA	NA	No synergy
CFS/IMP	69 (11/16)	56 (9/16)	12.5 (2/16)	0 (0/16)	Based on reported FICIs and MICs synergy was present at: 14A: 64/4, 14B: <32/4, 14D: <8/4, 14F: 8/4, 14G: <32/4, 14H: 64/4, 14I: 16/4, 14J: <16/4, 14L: 16/4, 14N: <16/4, 14O: 32/4 mg/L.
IMP/FOF	55 (11/20)	25 (5/20)	25 (5/20)	5 (1/20)	Based on reported FICIs and MICs synergy was present at: 14B: <32/4, 14C: 64/4, 14D: 64/4, 14E: <64/4, 14G: 32/4, 14H: 32/4, 14I: 32/4, 14K: 32/4, 14L: 64/4, 14M: 64/4, 14O: 64/4 mg/L.
**Wei W, 2017 [30]**					
CST/LVX	0 (0/1)	NA	NA	NA	No synergy
**Wei WJ, 2017 [31]**					
CST/CHL	100 (2/2)	50 (1/2)	50 (1/2)	0 (0/2)	Synergy at 0.5/64 and 0.5/2 mg/L
**Bae S, 2016 [32]**					
CST/RIF	100 (9/9)	22 (2/9)	0 (0/9)	78 (7/9)	
CST/TEC	44 (4/9)	0 (0/9)	0 (0/9)	44 (4/9)	Only presence (FICI≤0.5) or not of synergy is reported. Based on the available data it was possible to confirm synergy at concentrations below breakpoints in only few cases (CST/RIF 2/9, CST/MEM 1/9)
CST/VAN	78 (7/9)	0 (0/9)	0 (0/9)	78 (7/9)	
CST/MEM	33 (3/9)	11 (1/9)	0 (0/9)	22 (2/9)	
CST/ATM	44 (4/9)	0 (0/9)	0 (0/9)	44 (4/9)	
CST/CAZ	44 (4/9)	0 (0/9)	0 (0/9)	44 (4/9)	
CST/SAM	0 (0/8)	0 (0/8)	0 (0/8)	0 (0/8)	

Study-combinations	Synergy % (n/N)	Synergy at concentrations ≤ breakpoints % (n/N)	Synergy at concentrations > breakpoints % (n/N)	Synergy but unclear if at concentration ≤ breakpoints % (n/N)	Notes
CST/SXT	13 (1/7)	0 (0/7)	0 (0/7)	13 (1/7)	
CST/AMK	33 (3/6)	0 (0/6)	0 (0/6)	33 (3/6)	
CST/AZM	13 (1/8)	0 (0/8)	0 (0/8)	13 (1/8)	
CST/TGC	0 (0/9)	NA	NA	NA	
**Bowler SL, 2016 [33]**					
CST/FA	100 (3/3)	0 (0/3)	0 (0/3)	100 (3/3)	Only FICI range and MIC range are reported and it was not possible to extract if synergy was present at concentrations below breakpoints
**Laishram S, 2016 [35]**					
SUL/MEM	32 (16/50)	0 (0/50)	0 (0/50)	32 (16/50)	Only FICI range and MIC range are reported and it was not possible to extract if synergy was present at concentrations below breakpoints
**Leite GC, 2016 [36]**					
CST/RIF	100 (4/4)	50 (2/4)	0 (0/4)	50 (2/4)	
CST/VAN	100 (7/7)	0 (0/7)	0 (0/7)	100 (7/7)	
CST/TGC	0 (0/1)	NA	NA	NA	
CST/FOF	0 (0/6)	NA	NA	NA	
CST/GEN	0 (0/1)	NA	NA	NA	Based on reported MICs and FICIs
CST/MEM	100 (7/7)	0 (0/7)	100 (7/7)	0 (0/7)	
CST/IMP	100 (7/7)	0 (0/7)	100 (7/7)	0 (0/7)	
FOF/GEN	91 (10/11)	27 (3/11)	64 (7/11)	0 (0/11)	
FOF/AMK	96 (26/27)	41 (11/27)	55 (15/27)	0 (0/27)	
**Yang H, 2016 [38]**					
CST/VAN	100 (1/1)	0 (0/1)	0 (0/1)	100 (1/1)	Based on a reported FICI of 0.188 synergy was

Study-combinations	Synergy % (n/N)	Synergy at concentrations ≤ breakpoints % (n/N)	Synergy at concentrations > breakpoints % (n/N)	Synergy but unclear if at concentration ≤ breakpoints % (n/N)	Notes
					present at either 0.5/16 or 1/32 mg/L
**Yang YS, 2016 [39]**					
MEM/MIN	0 (0/3)	NA	NA	NA	No synergy
CFS/MIN	0 (0/2)	NA	NA	NA	No synergy
**García-Salguero C, 2015 [42]**					
AMK/IMP	100 (2/2)	0 (0/2)	100 (2/2)	0 (0/2)	Synergy at: Ab26: 64/32 or 16/128 mg/L, Ab66: 128/128 mg/L
AMK/MEM	100 (2/2)	0 (0/2)	100 (2/2)	0 (0/2)	Synergy at: Ab26: 64/32 or 32/64 mg/L, Ab66: 128/16 mg/L
AMK/FOF	0 (0/2)	NA	NA	NA	No synergy
PLZ/IMP	75 (6/8)	0 (0/8)	63 (5/8)	13 (1/8)	Synergy at: Ab2: 2/16 mg/L, AB26: 4/128 mg/L, Ab66: 4/64 or 2/128 mg/L, AB80: 8/2 or 2/8 mg/L, AB102: 2/16 mg/L, AB 113: 2/4 or 1/8 mg/L
PLZ/MEM	50 (2/4)	0 (0/4)	50 (2/4)	0 (0/4)	Synergy at: Ab26: 4/64 mg/L, Ab125: 16/32 mg/L
PLZ/FOF	11 (1/9)	0 (0/9)	11 (1/9)	0 (0/9)	Synergy at: Ab80: 8/16 mg/L
**Marie MA, 2015 [43]**					
SUL/MEM	44 (24/54)	0 (0/54)	0 (0/54)	44 (24/54)	Exact FICIs or combination MICs not reported.
MEM/TZB	41 (22/54)	0 (0/54)	0 (0/54)	41 (22/54)	Exact FICIs or combination MICs not reported.
**Vourli S, 2015 [45]**					
CST/MEM	100 (2/2)	0 (0/2)	100 (2/2)	0 (0/2)	Synergy at MEM concentrations 16-64mg/L and CST concentrations 0.0625-1.
CST/SAM	0 (0/2)	NA	NA	NA	No synergy
SAM/MEM	0 (0/5)	NA	NA	NA	No synergy
**Majewski P, 2014**					

Study-combinations	Synergy % (n/N)	Synergy at concentrations ≤ breakpoints % (n/N)	Synergy at concentrations > breakpoints % (n/N)	Synergy but unclear if at concentration ≤ breakpoints % (n/N)	Notes
**[47]**					
IMP/RIF	40 (4/10)	0 (0/10)	20 (2/10)	20 (2/10)	Synergy at: Isolate 12 (FICI=0.5): 16/1 mg/L, Isolate 34 (FICI=0.5): 16/1 mg/L, Isolate 53 (FICI=0.375): 8/0.5 or 4/1 mg/L, Isolate 91 : 8/0.5 or 4/1 mg/L,
**Percin D, 2014 [50]**					
CST/VAN	90 (9/10)	0 (0/10)	0 (0/10)	90 (9/10)	MICs for each isolate not available.
**Sun Y, 2014 [51]**					
CFS/MEM	0 (0/11)	NA	NA	NA	No synergy
MEM/AMK	0 (0/9)	NA	NA	NA	No synergy
MEM/CIP	9 (1/11)	0 (0/11)	9 (1/11)	0 (0/11)	Synergy at 16/32 mg/L
MEM/AZM	0 (0/12)	NA	NA	NA	No synergy
**Wang Y, 2014 [52]**					
IMP/RIF	67 (12/16)	61 (11/16)	6 (1/16)	0 (0/16)	Synergy at: 1/2, 2/2, 0.5/1, 2/1, 2/1, 4/4, 2/1, 2/1, 4/1, 8/4, 8/0.25, 1/1, 16/8, 8/1 and 16/2 mg/L
**Cetin ES, 2013 [53]**					
RIF/SAM	100 (7/7)	57 (4/7)	14 (1/7)	29 (2/7)	Synergy at: Isolate 5: 1/1 or 0.125/8 mg/L, Isolate 11: 0.5/4 mg/L, Isolate 12: 1/2 or 0.125/16 mg/L, Isolate 14: 4/4 or 0.5/32 mg/L, Isolate 15: 1/1 or 0.125/8 mg/L, Isolate 16: 1/2 or 0.125/16 mg/L, Isolate 20: 1/8 mg/L
RIF/CFS	29 (2/7)	14 (1/7)	0 (0/7)	14 (1/7)	Synergy at: Isolate 11: 1/16 mg/l, isolate 20: 64/0.5 or 32/1 mg/L
**O'Hara JA, 2013 [56]**					
CST/DOR	67 (2/3)	33 (1/3)	33 (1/3)	0 (0/3)	Synergy at: JA637 (FICI 0.31): 64/2 or 16/8 mg/L, JA566 (FICI 0.13): 0.25/2 mg/L
CST/VAN	100 (3/3)	0 (0/3)	67 (2/3)	33 (1/3)	Synergy at: JA637 (FICI 0.28): 64/8 or 8/64 mg/L,

Study-combinations	Synergy % (n/N)	Synergy at concentrations ≤ breakpoints % (n/N)	Synergy at concentrations > breakpoints % (n/N)	Synergy but unclear if at concentration ≤ breakpoints % (n/N)	Notes
					JA566 (FICI 0.14): 0.5/4 or 0.0625/32 mg/L, JA942 (FICI 0.25): 32/32 mg/L
DOR/VAN	0 (0/3)	NA	NA	NA	No synergy
**Principe L, 2013 [57]**					
DOR/TGC	100 (3/3)	33 (1/3)	0 (0/3)	67 (2/3)	Synergy at; ≤8/2, ≤16/2, ≤32/1.
DOR/AMK	25 (1/4)	0 (0/4)	0 (0/4)s	25 (1/4)	Synergy at ≤16/64 mg/L
DOR/CST	0 (0/1)	NA	NA	NA	
SAM/DOR	0 (0/1)	NA	NA	NA	
DOR/RIF	0 (0/1)	NA	NA	NA	
**Deveci A, 2012 [59]**					
SUL/CAZ	70 (7/10)	10 (1/10)	60 (6/10)	0 (0/10)	Synergy at: 16/32, 8/32, 32/2, 32/2, 64/4, 32/2 and 8/4 mg/L,
SUL/MEM	0 (0/9)	NA	NA	NA	
SUL/CRO	40 (4/10)	0 (0/10)	40 (4/10)	0 (0/10)	Synergy at: 16/512, 256/64, 16/64, 32/128 mg/L
SUL/CIP	80 (8/10)	20 (2/10)	60 (6/10)	0 (0/10)	Synergy at: 8/0.5, 16/2, 16/8, 128/1, 64/1, 128/0.5, 64/1, 8/1 mg/L
SUL/GEN	80 (8/10)	20 (2/10)	60 (6/10)	0 (0/10)	Synergy at: 8/16, 8/8, 8/64, 16/16, 32/8, 32/8, 32/8, 8/8 mg/L
SUL/FEP	44 (4/9)	0 (0/9)	44 (4/9)	0 (0/9)	Synergy at: 64/4, 64/4, 64/4, 32/4 mg/L
**Vidaillac C, 2012 [61]**					
CST/VAN	100 (1/1)	100 (1/1)	0 (0/1)	0 (0/1)	FICI 0.18 → Synergy at: 1/8 or 0.5/16 mg/L
CST/TMP	100 (1/1)	0 (0/1)	100 (1/1)	0 (0/1)	FICI 0.37 → Synergy at: 2/4 or 1/8 mg/L
CST/SXT	100 (1/1)	100 (1/1)	0 (0/1)	0 (0/1)	FICI 0.20 (?, <0.25) → Synergy at: <2/2/38 mg/L
**Santimaleeworagun**					

Study-combinations	Synergy % (n/N)	Synergy at concentrations ≤ breakpoints % (n/N)	Synergy at concentrations > breakpoints % (n/N)	Synergy but unclear if at concentration ≤ breakpoints % (n/N)	Notes
**W, 2011 [62]**					
SUL/IMP	0 (0/6)	NA	NA	NA	No synergy
SUL/FOF	67 (4/6)	33 (2/6)	17 (1/6)	17 (1/6)	Synergy at: AB164 (FICI 0.48<0.5): <8/32 mg/L, AB167 (FICI 0.37): 8/32 or 4/64 mg/L, AB198 (FICI 0.28): 8/4 or 1/32 mg/L, AB313 (FICI 0.47<0.5): <16/128 mg/L
**Tan TY, 2011 [64]**					
PMB/TGC	75 (3/4)	0 (0/4)	50 (2/4)	25 (1/4)	Based on reported FICIs and MICs synergy was present in CHBD at the following concentrations: For isolate 18351: 4/0.5 or 1/2 mg/dl. For isolate 9447: 4/1 mg/L. For isolate 11171: 4/1 mg/L. No synergy in TKA at 2/2 mg/L.
PMB/RIF	100 (3/3)	33 (1/3)	33 (1/3)	33 (1/3)	Based on reported FICIs and MICs synergy was present in CHBD at the following concentrations: For isolate 27640: 2/1 or 0.5/4 mg/LFor isolate 9447: 1/1 or 0.5/2 mg/dl. For isolate 11171: 4/1 mg/L. For isolate 11171: 4/8 mg/L.
TGC/RIF	33 (1/3)	0 (0/3)	33 (1/3)	0 (0/3)	Based on reported FICIs and MICs synergy was present in CHBD at 4/4 or 2/8 mg/L.
**Principe L, 2009 [73]**					
TGC/LVX	22 (4/18)	0 (0/18)	22 (4/18)	0 (0/18)	Synergy present in checkerboard at: 0.25/4 (isolate 5, 11 and 75), 2/4 (isolate 16).
TGC/TZP	0 (0/18)	NA	NA	NA	No synergy
TGC/AMK	14 (2/14)	7 (1/14)	7 (1/14)	0 (1/14)	Synergy present in checkerboard at: 1/16 (isolate 11) and 1/64 mg/L (isolate 71)

Study-combinations	Synergy % (n/N)	Synergy at concentrations ≤ breakpoints % (n/N)	Synergy at concentrations > breakpoints % (n/N)	Synergy but unclear if at concentration ≤ breakpoints % (n/N)	Notes
TGC/IMP	8 (1/12)	0 (0/12)	8 (1/12)	0 (0/12)	Synergy present in checkerboard at 0.25/8 mg/L
TGC/RIF	0 (0/13)	NA	NA	NA	No synergy
SAM/TGC	0 (0/6)	NA	NA	NA	No synergy
**Sader HS, 2005 [77]**					
SAM/FEP	100 (2/2)	50 (1/2)	50 (1/2)	0 (0/2)	Combination MICs: 8/4/32 and 16/8/16 mg/L
**Sader HS, 2005 [78]**					
ATM/FEP	0 (0/3)	NA	NA	NA	No synergy at 8/8 mg/L
ATM/CAZ	0 (0/4)	NA	NA	NA	No synergy at 8/8 mg/L
ATM/MEM	0 (0/3)	NA	NA	NA	No synergy at 8/4 mg/L
SAM/ATM	0 (0/1)	NA	NA	NA	No synergy at 8/4/8 mg/L
**Fernández-Cuenca F, 2003 [83]**					
AZM/IMP	0 (0/2)	NA	NA	NA	No synergy
AZM/CAZ	33 (1/3)	0 (0/3)	33 (1/3)	0 (0/3)	Synergy at 64/128 mg/L
AZM/AMK	0 (0/2)	NA	NA	NA	No synergy
AZM/CIP	0 (0/3)	NA	NA	NA	No synergy

4.5 Proportion of synergy and assessment of clinical relevance of observed synergy in time-kill assays (study level data)

Study-combinations	Synergy % (n/N)	Synergy at concesntrations ≤ breakpoints % (n/N)	Synergy at concentrations > breakpoints % (n/N)	Synergy but unclear if at concentration ≤ breakpoints % (n/N)	Concentrations at which synergy was evaluated
**Nwabor OF 2021 [4]**					TKA conducted at the following concentrations: FOF 1xMIC, other at ½ and ¼ x MIC
FOF/DOR		NA	NA	NA	No synergy
FOF/CIP		NA	NA	NA	No synergy
FOF/TGC					Synergy at 128/2 and 128/1 mg/L
**Li J, 2020 [6]**					TKA only conducted at 0.5x and 1x MIC
CST/MEM	100 (1/1)	0 (0/1)	100 (1/1)	0 (0/1)	Synergy at: 4/32 and 4/16 mg/L.
CST/LVX	100 (1/1)	0 (0/1)	100 (1/1)	0 (0/1)	Synergy at: 4/16 and 4/8 mg/L.
**Mohd Sazlly Lim S 2020 [8], 2021 [9], 2021 [10,85]**					
SUL/FOF	50 (2/4)	0 (0/4)	50 (2/4)	0 (0/4)	Synergy at 128/128 (n=2) mg/L. Based on semi-mechanistic PK/PD modelling, the SUL/FOF regimen at best demonstrated a probability of target attainment of 2-log10 kill at 24 h of 72%.
SUL/MEM	100 (2/2)	0 (0/2)	100 (2/2)	0 (0/2)	Synergy at 64/32 and 128/64 mg/L. Based on semi-mechanistic PK/PD modelling, the MEM/SUL regimen at best demonstrated a probability of target attainment of 2-log10 kill at 24 h of 34%.

Study-combinations	Synergy % (n/N)	Synergy at concesntrations ≤ breakpoints % (n/N)	Synergy at concentrations > breakpoints % (n/N)	Synergy but unclear if at concentration ≤ breakpoints % (n/N)	Concentrations at which synergy was evaluated
**Rodriguez CH, 2020 [11]**					
SUL/AVI	100 (1/1)	100 (1/1)	0 (0/1)	0 (0/1)	Bactericidal activity at SUL/AVI concentrations 8/4mg/L
**Gaudereto JJ, 2019 [12]**					
CZA/MEM	0 (0/11)	NA	NA	NA	TKA conducted at 1x and 0.5x MIC. No synergy.
**Mataraci Kara E, 2019 [14]**					
CST/CZA	100 (2/2)	0 (0/2)	100 (2/2)	0 (0/2)	Synergy assessed in TKA only 1x or 4x the MIC
CZA/LVX	75 (3/4)	0 (0/4)	75 (3/4)	0 (0/4)	
CZA/TGC	75 (3/4)	0 (0/4)	75 (3/4)	0 (0/4)	
CZA/TOB	50 (1/2)	0 (0/2)	50 (1/2)	0 (0/2)	
CZA/MEM	75 (3/4)	0 (0/4)	75 (3/4)	0 (0/4)	
**Oliva A, 2019 [17]**					
CST/MEM	100 (1/1)	0 (0/1)	100 (1/1)	0 (0/1)	Synergy at 4/64 mg/L
CST/RIF	100 (2/2)	0 (0/2)	100 (2/2)	0 (0/2)	Synergy at 4/2 and 4/256 mg/L
CST/VAN	100 (2/2)	0 (0/2)	100 (2/2)	0 (0/2)	Synergy at at 4/16 and 256/16 mg/L
**Phee LM, 2015 [19] and 2019 [20]**					
CST/FA	100 (1/1)	0 (0/1)	100 (1/1)	0 (0/1)	Synergy at 2/16 mg/L
**Poulakou G, 2019 [21]**					
CST/DAP	0 (0/1)	NA	NA	NA	No synergy

Study-combinations	Synergy % (n/N)	Synergy at concesntrations ≤ breakpoints % (n/N)	Synergy at concentrations > breakpoints % (n/N)	Synergy but unclear if at concentration ≤ breakpoints % (n/N)	Concentrations at which synergy was evaluated
**Shinohara DR, 2019 [22]**					
PMB/VAN	67 (2/3)	0 (0/3)	67 (2/3)	0 (0/3)	TKA only conducted at 0.5 x MIC (8/128, 8/128 and 2/128 mg/L)
**Wang J, 2019 [23]**					
CST/MEM	100 (2/2)	0 (0/2)	100 (2/2)	0 (0/2)	Synergy only assessed at 4/16 and 8/16mg/L.
**Singham-In U, 2018 [25]**					
IMP/FOF	89 (8/9)	0 (0/9)	89 (8/9)	0 (0/9)	Concentrations used in TKA were above breakpoints
**Lenhard JR, 2017 [27,28]**					
PMB/MEM	0 (0/2)	NA	NA	NA	TKA conducted at: SAM 132/70 mg/L, MEM 55mg/L, PMB 1.5 mg/L
PMB/SAM	0 (0/2)	NA	NA	NA	
MEM/SAM	0 (0/2)	NA	NA	NA	
PMB/MEM/SAM	50 (1/2)	0 (0/2)	50 (1/2)	0 (0/2)	
**Wei W, 2017 [30]**					
CST/LVX	0 (0/1)	NA	NA	NA	No synergy
**Wei WJ, 2017 [31]**					
CST/CHL	100 (2/2)	0 (0/2)	100 (2/2)	0 (0/2)	Synergy at 2/32mg/L
**Bowler SL, 2016 [33]**					
CST/FA	67 (2/3)	0 (0/3)	67 (2/3)	0 (0/3)	TKA conducted at; CST 2 mg/L, VAN 20 mg/L, DOR 8 mg/L, FA 8 mg/L
CST/VAN	33 (1/3)	33 (1/3)	0 (0/3)	0 (0/3)	

Study-combinations	Synergy % (n/N)	Synergy at concesntrations ≤ breakpoints % (n/N)	Synergy at concentrations > breakpoints % (n/N)	Synergy but unclear if at concentration ≤ breakpoints % (n/N)	Concentrations at which synergy was evaluated
CST/DOR	33 (1/3)	33 (1/3)	0 (0/3)	0 (0/3)	
**Laishram S, 2016 [35]**					
SUL/MEM	58 (29/50)	0 (0/50)	8 (4/50)	50 (25/50)	Based on concentrations used (0.5x MIC) and reported MIC50 synergy was present at above breakpoints in TKA against at least 4 isolates.
**Park GC, 2016 [37]**					
CST/DOR	59 (10/17)	59 (10/17)	0 (0/17)	0 (0/17)	TKA assays were conducted at the following concentrations: CST 2mg/L, TGC 2mg/L, DOR 8mg/L
CST/TGC	58 (7/12)	58 (7/12)	0 (0/12)	0 (0/12)	
TGC/DOR	11 (5/46)	11 (5/46)	0 (0/46)	0 (0/46)	
**Yang H, 2016 [38]**					
CST/VAN	0 (0/1)	0 (0/1)	0 (0/1)	0 (0/1)	No synergy at 2/20 mg/L
**Yang YS, 2016 [39]**					
MEM/MIN	0 (0/3)	NA	NA	NA	No synergy at
CFS/MIN	50 (1/2)	0 (0/2)	50 (1/2)	0 (0/2)	Synergy at 16/16/16 mg/L
**García-Salguero C, 2015 [42]**					
PLZ/FOF	0 (0/2)	NA	NA	NA	No synergy at 4/64 mg/L. Regrowth at 24 hours, despite initial bactericidal killing
**Rodriguez CH, 2015 [44]**					
IMP/MIN	0 (0/1)	NA	NA	NA	TKA conducted at the following concentrations: IMP 8mg/L, MIN 4 mg/L, RIF 4 mg/L
IMP/RIF	0 (0/1)	NA	NA	NA	

Study-combinations	Synergy % (n/N)	Synergy at concesntrations ≤ breakpoints % (n/N)	Synergy at concentrations > breakpoints % (n/N)	Synergy but unclear if at concentration ≤ breakpoints % (n/N)	Concentrations at which synergy was evaluated
**Galani I, 2014 [46]**					
CST/DAP	0 (0/4)	NA	NA	NA	TKA conducted at 0.25, 0.5 and 1x MIC
**Nastro M, 2014 [48]**					
CST/RIF	100 (4/4)	0 (0/4)	100 (4/4)	0 (0/4)	Synergy at RIF 4 mg/L, CST 2 mg/L
**Oleksium LM, 2014 [49]**					
CST/DOR	83 (5/6)	83 (5/6)	0 (0/6)	0 (0/6)	TKA conducted at the following concentrations: CST 2mg/L, DOR 8mg/L, SUL 4mg/L
CST/SUL	33 (2/6)	33 (2/6)	0 (0/6)	0 (0/6)	
CST/DOR/SUL	100 (6/6)	100 (6/6)	0 (0/6)	0 (0/6)	
SUL/DOR	24 (4/17)	24 (4/17)	0 (0/17)	0 (0/17)	
**Percin D, 2014 [50]**					
CST/VAN	100 (10/10)	100 (10/10)	0 (0/10)	0 (0/10)	TKA was conducted at VAN 20 mg/L and CST 2 mg/L.
**O'Hara JA, 2013 [56]**					
CST/DOR	100 (3/3)	100 (3/3)	0 (0/3)	0 (0/3)	TKA conducted at: CST 2mg/L, DOR 8mg/L, VAN 20 mg/L.
CST/VAN	67 (2/3)	67 (2/3)	0 (0/3)	0 (0/3)	
DOR/VAN	0 (0/3)	NA	NA	NA	
CST/VAN/DOR	100 (3/3)	100 (3/3)	0 (0/3)	0 (0/3)	
**Queenan AM, 2013 [58]**					
DOR/CIP	0 (0/1)	NA	NA	NA	TKA conducted at maximum clinically achievable concentrations (DOR 18.8 mg/L, CIP 4.56 mg/L, LVX 11.5 mg/L).
DOR/LVX	0 (0/1)	NA	NA	NA	
**Peck KR, 2012 [60]**					

Study-combinations	Synergy % (n/N)	Synergy at concesntrations ≤ breakpoints % (n/N)	Synergy at concentrations > breakpoints % (n/N)	Synergy but unclear if at concentration ≤ breakpoints % (n/N)	Concentrations at which synergy was evaluated
CST/IMP	0 (0/2)	NA	NA	NA	TKA only conducted at 0.5x and 1xMIC. Graphs available only for CST/IMP. Discordant results comparing the table to the TKA curves (reported synergy in the table but no synergy examining the TKA curve).
CST/RIF	100 (1/1)	0 (0/1)	0 (0/1)	100 (1/1)	
SAM/IMP	100 (6/6)	0 (0/6)	0 (0/6)	100 (6/6)	
**Vidaillac C, 2012 [61]**					
CST/VAN	100 (1/1)	0 (0/1)	100 (1/1)	0 (0/1)	TKA conducted only at 0.25x (CST 2 mg/L, VAN 32 mg/L, TMP 8 mg/L and SXT 4/76.5 mg/L) and 0.5x (CST 8 mg/L, VAN 64 mg/L, TMP 16 mg/L and SXT 8/153 mg/L) the MIC
CST/TMP	100 (1/1)	0 (0/1)	100 (1/1)	0 (0/1)	
CST/SXT	100 (1/1)	0 (0/1)	100 (1/1)	0 (0/1)	
**Pachón-Ibáñez ME, 2011 [63]**					
RIF/IMP	100 (2/2)	0 (0/2)	100 (2/2)	0 (0/2)	TKA conducted only at 1x MIC and at Cmax (i.e. at concentrations > breakpoints)
RIF/SUL	100 (2/2)	0 (0/2)	100 (2/2)	0 (0/2)	
**Santimaleeworagun W, 2011 [62]**					
SUL/FOF	83 (5/6)	0 (0/6)	83 (5/6)	0 (0/6)	TKA was only conducted at SUL 1xMIC (i.e. above breakpoints of resistance).
**Tan TY, 2011 [64]**					
PMB/TGC	0 (0/4)	NA	NA	NA	2/2 mg/L
PMB/RIF	33 (1/3)	33 (1/3)	0 (0/3)	0 (0/3)	2/2 mg/L

Study-combinations	Synergy % (n/N)	Synergy at concesntrations ≤ breakpoints % (n/N)	Synergy at concentrations > breakpoints % (n/N)	Synergy but unclear if at concentration ≤ breakpoints % (n/N)	Concentrations at which synergy was evaluated
TGC/RIF	0 (0/3)	NA	NA	NA	2/2 mg/L
**Pankuch GA, 2010 [67]**					
DOR/LVX	60 (3/5)	0 (0/5)	60 (3/5)	0 (0/5)	Synergy at 4/16, 8/16 and 16/18 mg/L
DOR/AMK	100 (2/2)	0 (0/2)	100 (2/2)	0 (0/2)	Synergy at 32/64 and 8/512 mg/L
DOR/CST	100 (4/4)	0 (0/4)	100 (4/4)	0 (0/4)	Synergy at 4/2, 4/8, 8/4 and 8/2 mg/L
**Rodriguez CH, 2010 [68]**					
IMP/RIF	0 (0/4)	NA	NA	NA	8/4 mg/L.
IMP/GEN	0 (0/2)	NA	NA	NA	8/4 mg/L.
**Urban C, 2010 [69]**					
DOR/RIF	40 (2/5)	20 (1/5)	20 (1/5)	0 (0/5)	TKA conducted at 1/4 x MIC. Synergy at: 2/8 and 4/8 mg/L
**Yuan Z, 2010 [70] and Lim TP, 2008 [71]**					
AMK/LVX	0 (0/1)	NA	NA	NA	
AMK/FEP	100 (1/1)	0 (0/1)	100 (1/1)		Synergy was defined based on the interactive index method. Based on the available graphs it seems that >2 log CFU/ml reduction were only observed at concentrations > breakpoints
FEP/LVX	100 (1/1)	0 (0/1)	100 (1/1)		
**Lim TP, 2009 [72]**					
MEM/RIF	100 (2/2)	0 (0/2)	100 (2/2)	0 (0/2)	Synergy at 64/2 mg/L
TGC/MEM	0 (0/1)	NA	NA	NA	No synergy at 2/64 mg/L

Study-combinations	Synergy % (n/N)	Synergy at concesntrations ≤ breakpoints % (n/N)	Synergy at concentrations > breakpoints % (n/N)	Synergy but unclear if at concentration ≤ breakpoints % (n/N)	Concentrations at which synergy was evaluated
TGC/RIF	100 (1/1)	100 (1/1)	0 (0/1)	0 (0/1)	Synergy at 2/2 mg/L
**Principe L, 2009 [73]**					
TGC/LVX	0 (0/2)	NA	NA	NA	Regrowth in TKA at 0.25/4 mg/L
TGC/AMK	100 (1/1)	100 (1/1)	0 (0/1)	0 (0/1)	Synergy in TKA at 1/64 mg/L
TGC/IMP	0 (0/1)	NA	NA	NA	Regrowth in TKA at 0.25/8 mg/L
**Lee CH, 2008 [75]**					
SUL/MEM	50 (1/2)	0 (0/2)	50 (1/2)	0 (0/2)	TKA conducted only at 1xMIC. Synergy at 16/64 mg/L
**Lee NY, 2007 [76]**					
SUL/IMP	0 (0/4)	NA	NA	NA	Although only partial synergy (FICIs 0.56-0.75) is reported in checkerboard the combination MIC in agar dilution were ≤ breakpoints; 8/0.5, 8/2, 8/2, 8/2 mg/L.
SUL/MEM	0 (0/4)	NA	NA	NA	Although only partial synergy (FICIs 0.56-0.75) is reported in checkerboard the combination MIC in agar dilution were ≤ breakpointss; 8/1, 8/4, 8/4, 8/4 mg/L.
**Choi JY, 2004 [79]**					
SUL/IMP	100 (1/1)	0 (0/1)	100 (1/1)	0 (0/1)	TKA conducted at 0.5x and 1x MIC. Synergy at 32/8 mg/L
**Jung R, 2004 [80]**					
FEP/MXF	100 (2/2)	0 (0/2)	100 (2/2)	0 (0/2)	TKA conducted at 0.5x and 1x MIC. Synergy at 32/8 and 32/4 mg/L.
**Montero A, 2004 [81]**					

Study-combinations	Synergy % (n/N)	Synergy at concesntrations ≤ breakpoints % (n/N)	Synergy at concentrations > breakpoints % (n/N)	Synergy but unclear if at concentration ≤ breakpoints % (n/N)	Concentrations at which synergy was evaluated
IMP/RIF	100 (2/2)	0 (0/2)	100 (2/2)	0 (0/2)	TKA conducted at 1/2 to 1/32 x MIC. Synergy at 2/4 mg/L (strain D) and 32/2mg/L (Strain E). Strain D: No differences compared to monotherapy in the mouse model. Strain E: Significantly reduced lung bacterial counts, no significant reduction of bacteraemia, similar survival (100% with the combination + 100% with RIF monotherapy).
IMP/SUL	0 (0/1)	NA	NA	NA	TKA conducted at 1/2 to 1/32 x MIC. No synergy in TKA. Not evaluated in the animal model.
**Yoon J, 2004** [82]					
PMB/IMP	100 (1/1)	0 (0/1)	100 (1/1)	0 (0/1)	Synergy at 2/8 mg/L
**Roussel-Delvallez M, 1996** [84]					
IMP/SUL/AMK	0 (0/8)	NA	NA	NA	TKA was conducted at following concentrations: TIM 112 mg/L, TZP 100 mg/L, IMP 25 mg/L, AMK 15 mg/L. Only mean killing is reported. Initial bactericidal killing was observed with some combinations-strains but "regrowth was observed for all strains at 24 hours".
IMP/TIM/AMK	0 (0/8)	NA	NA	NA	
TZP/SUL/AMK	0 (0/8)	NA	NA	NA	
TZP/TIM/AMK	0 (0/8)	NA	NA	NA	

4.6 Proportion of synergy and assessment of clinical relevance of observed synergy in studies using dynamic in vitro PK/PD models (study level data)

Study-combinations	Method	Synergy % (n/N)	Comments
**Lenhard JR, 2017 [27,28]**			
PMB/MEM	HFIM	0 (0/1)	Doses simulating human regimens were used (PMB 3.33mg/kg then 1.43mg/kg every 12 hours, MEM 2gr every 8 hour as 3h-infusions, SAM 8/4g every 8 hours as 3h-infusions).
PMB/SAM		0 (0/1)	
MEM/SAM		0 (0/1)	
PMB/MEM/SAM		100 (1/1)	
**Yuan Z, 2010 [70] and Lim TP, 2008 [71]**			
AMK/LVX	HFIM	0 (0/1)	Regrowth despite initial killing at 4 hours.
AMK/FEP		0 (0/1)	Regrowth despite initial killing at 4 hours.
**Córdoba J, 2015 [41]**			
CST/IMP	Other dynamic in vitro PK/PD model	0 (0/1)	Simulation of human treatment regimens
CST/DAP		100 (1/1)	
IMP/ETP		0 (0/3)	
RIF/CFS		0 (0/7)	
**Housman ST, 2013 [54]**			Simulated regimens: SAM 9g q8h (3h inf), DOR 2gr q8h (4h inf), TGC 200mg q12h (0.5h inf).
TGC/DOR	Other dynamic in vitro PK/PD model	0 (0/2)	No synergy.
SAM/DOR		0 (0/3)	Increased killing with SAM/DOR vs monotherapies against all 3 strains but with regrowth by 24 hours.
SAM/TGC		0 (0/1)	No synergy.
**Lee HJ, 2013 [55]**			
CST/RIF	Other dynamic in vitro PK/PD model	100 (1/1)	Regimens mimicking human serum concentration after usual doses in critically-ill patients.

4.7 Proportion of synergy and assessment of clinical relevance of observed synergy in studies using animal models (study level data)

Study-combinations	Method	Outcomes assessed	Synergy % (n/N)	Comments
**Cebrero-Cangueiro T, 2021 [1] and Nordmann P, 2020 [2]**				
MEM/IMP	Intraperitoneal infection mouse model	Survival, sterilization of cultures, bacterial loads	0 (0/2)	Decreased bacterial loads with combination vs monotherapy. But similar mortality and bacterial clearance comparing meropenem monotherapy to combination therapy.
**Poulakou G, 2019 [21]**				
CST/DAP	Intraperitoneal infection mouse model	Survival and bacterial loads	100 (1/1)	The combination significantly improved survival and reduced bacterial loads in tissues compared to monotherapies.
**Wei W, 2017 [30]**				
CST/LVX	G. mellonella model	Survival	0 (0/1)	Same survival comparing combination therapy to monotherapy
**Yang H, 2016 [38]**				
CST/VAN	*G. mellonella* model	Survival	100 (1/1)	Survival rate in *G. mellonella* model higher than monotherapies, but high survival even with monotherapies.
**O'Hara JA, 2013 [56]**				
CST/DOR	*G. mellonella* model (simulation of human doses)	Survival	0 (0/3)	No synergy
CST/VAN			0 (0/3)	
DOR/VAN			100 (3/3)	The clinical relevance of the *G. mellonella* model is unclear because of mechanisms of action likely not relevant to humans; high survival even with DOR and VAN monotherapies, and high survival with DOR/VAN despite lack of in vitro synergy
CST/VAN/DOR			100 (3/3)	
**Queenan AM, 2013 [58]**				

Study-combinations	Method	Outcomes assessed	Synergy % (n/N)	Comments
DOR/CIP	intraperitoneal infection mouse model	Survival	0 (0/1)	No synergy
DOR/LVX			100 (1/1)	Improved survival in the mouse model (the isolate had relatively low MICs: DOR 16 mg/L and LVX 8 mg/L).
**Pachón-Ibáñez ME, 2011 [63]**				
RIF/IMP	Pneumonia mouse model	Survival, sterilization of cultures, bacterial loads	0 (0/2)	In the animal model survival with RIF/IMP (80 and 33%) and RIF/SUL (60 and 53%) did not differ significantly compared to RIF monotherapy (73 and 40%). Lung clearance and blood culture sterilization was higher against one of the two strains with RIF/SUL.
RIF/SUL			50 (1/2)	
**Pachón-Ibáñez ME, 2010 [66]**				
SUL/IMP	Pneumonia and meningitis mouse models	Survival, sterilization of cultures, bacterial loads	100 (1/1)	Higher survival and bacterial clearance in animal model compared to monotherapies.
RIF/IMP			0 (0/1)	Survival not improved comparing RIF monotherapy (71%) to combination therapy (60%), despite improved bacterial clearance.
RIF/SUL			0 (0/1)	Survival not improved comparing RIF monotherapy (71%) to combination therapy (47%), despite improved bacterial clearance.
**Yuan Z, 2010 [70] and Lim TP, 2008 [71]**				
AMK/LVX	Pneumonia mouse model	Survival, bacterial loads	0 (0/1)	Similar survival with AMK monotherapy.
AMK/FEP			1 (1/1)	Improved survival and reduction of tissue bacterial burden in the mouse model.
FEP/LVX			0 (0/1)	Similar survival with FEP monotherapy.
**Song YC, 2009 [74]**				
IMP/RIF	Pneumonia mouse model	Survival, sterilization of cultures,	100 (3/3)	Synergistic (≥2Δlog reduction in lung baterial loads compared to RIF monotherapy) against all 3 strains, but

Study-combinations	Method	Outcomes assessed	Synergy % (n/N)	Comments
		bacterial loads		100% survival with both monotherapy and combination.
RIF/AMK			0 (0/1)	Not better than monotherapy
IMP/AMK			0 (0/1)	Not better than monotherapy
**Montero A, 2004** [81]				
IMP/RIF	Mouse pneumonia model	Survival, sterilization of cultures, bacterial loads	50 (1/2)	Strain D: No differences compared to monotherapy in the mouse model. Strain E: Significantly reduced lung bacterial counts, no significant reduction of bacteraemia, similar survival (100% with the combination + 100% with RIF monotherapy).

4.8 Proportion of synergy and assessment of clinical relevance of observed synergy in other methods (study level data)

**Study-combinations**	**Method**	**Synergy % (n/N)**	**Synergy at concentrations ≤ breakpoints % (n/N)**	**Synergy at concentrations > breakpoints % (n/N)**	**Synergy but unclear if at concentration ≤ breakpoints % (n/N)**	**Concentrations at which synergy was evaluated**
**Limsrivanichakorn S, 2020 [7]**						
CFS/MXF	E-test fixed ratio method	2.5 (2/80)	0 (0/80)	0 (0/80)	2.5 (2/80)	Unclear at which concentration synergy was present
**Rodriguez CH, 2020 [11]**						
SUL/AVI	Agar dilution	92 (35/38)	92 (35/38)	0 (0/38)	0 (0/38)	Avibactam at a fixed concetration of 4mg/L reduced the MIC of SUL to ≤4mg/L in 35 of the 38 eligible isolates.
**Gaudereto JJ, 2019 [12]**						
CZA/MEM	DDST	1.8 (2/11)	0 (0/11)	0 (0/11)	1.8 (2/11)	
**Shinohara DR, 2019 [22]**						
PMB/VAN	Agar/disk	100 (3/3)	100 (3/3)	0 (0/3)	0 (0/3)	Agar containing CST at 0.5 x MIC (8, 8 and 4 mg/L)
PMB/VAN	Agar/gradient	100 (3/3)	100 (3/3)	0 (0/3)	0 (0/3)	
**Madadi-Goli N, 2017 [29]**						
TGC/LVX	Fixed-ratio E-test method	0 (0/7)	NA	NA	NA	No synergy
SAM/LVX		100 (7/7)	71 (5/7)	29 (2/7)	0 (0/7)	Combination MICs: 3/3, 4/4, 4/4, 4/4, 4/4, 6/6, 6/6 mg/L

Study-combinations	Method	Synergy % (n/N)	Synergy at concentrations ≤ breakpoints % (n/N)	Synergy at concentrations > breakpoints % (n/N)	Synergy but unclear if at concentration ≤ breakpoints % (n/N)	Concentrations at which synergy was evaluated
SAM/TGC		0 (0/7)	NA	NA	NA	No synergy
**Bae S, 2016 [32]**						MCBT conducted at concentrations equal to the breakpoints of resistance; concentrations: CST at 2 mg/L, SAM at 16/8 mg/L, AMK at 16 mg/L, AZM at 4 mg/L, ATM at 16 mg/L, CAZ at 16 mg/L, MEM at 8 mg/L, RIF at 2 mg/L, TGC at 2 mg/L, SXT at 4/76 mg/L, VAN at 4 mg/L, and TEC at 16 mg/L.
SAM/RIF	MCBT	13 (1/8)	13 (1/8)	0 (0/8)	0 (0/8)	
SAM/SXT		14 (1/7)	14 (1/7)	0 (0/7)	0 (0/7)	
SAM/TEC		13 (1/8)	13 (1/8)	0 (0/8)	0 (0/8)	
AMK/CAZ		17 (1/6)	17 (1/6)	0 (0/6)	0 (0/6)	
AMK/SXT		17 (1/6)	17 (1/6)	0 (0/6)	0 (0/6)	
AZM/CAZ		13 (1/8)	13 (1/8)	0 (0/8)	0 (0/8)	
AZM/SXT		17 (1/6)	17 (1/6)	0 (0/6)	0 (0/6)	
AZM/TEC		13 (1/8)	13 (1/8)	0 (0/8)	0 (0/8)	
ATM/CAZ		11 (1/9)	11 (1/9)	0 (0/9)	0 (0/9)	
ATM/SXT		14 (1/7)	14 (1/7)	0 (0/7)	0 (0/7)	
ATM/TEC		11 (1/9)	11 (1/9)	0 (0/9)	0 (0/9)	
CAZ/MEM		11 (1/9)	11 (1/9)	0 (0/9)	0 (0/9)	
CAZ/RIF		11 (1/9)	11 (1/9)	0 (0/9)	0 (0/9)	
CAZ/TGC		11 (1/9)	11 (1/9)	0 (0/9)	0 (0/9)	
CAZ/SXT		14 (1/7)	14 (1/7)	0 (0/7)	0 (0/7)	
CAZ/VAN		11 (1/9)	11 (1/9)	0 (0/9)	0 (0/9)	
MEM/RIF		11 (1/9)	11 (1/9)	0 (0/9)	0 (0/9)	
MEM/SXT		14 (1/7)	14 (1/7)	0 (0/7)	0 (0/7)	
MEM/TEC		11 (1/9)	11 (1/9)	0 (0/9)	0 (0/9)	
RIF/SXT		14 (1/7)	14 (1/7)	0 (0/7)	0 (0/7)	
SXT/VAN		14 (1/7)	14 (1/7)	0 (0/7)	0 (0/7)	
AMK/RIF		33 (2/6)	33 (2/6)	0 (0/6)	0 (0/6)	

Study-combinations	Method	Synergy % (n/N)	Synergy at concentrations ≤ breakpoints % (n/N)	Synergy at concentrations > breakpoints % (n/N)	Synergy but unclear if at concentration ≤ breakpoints % (n/N)	Concentrations at which synergy was evaluated
CAZ/TEC		22 (2/9)	22 (2/9)	0 (0/9)	0 (0/9)	
CST/RIF		100 (9/9)	100 (9/9)	0 (0/9)	0 (0/9)	
CST/TEC		100 (9/9)	100 (9/9)	0 (0/9)	0 (0/9)	
CST/VAN		89 (9/9)	89 (9/9)	0 (0/9)	0 (0/9)	
CST/MEM		89 (9/9)	89 (9/9)	0 (0/9)	0 (0/9)	
CST/ATM		89 (9/9)	89 (9/9)	0 (0/9)	0 (0/9)	
CST/CAZ		67 (6/9)	67 (6/9)	0 (0/9)	0 (0/9)	
CST/SAM		63 (5/8)	63 (5/8)	0 (0/8)	0 (0/8)	
CST/SXT		38 (3/8)	38 (3/8)	0 (0/8)	0 (0/8)	
CST/AMK		67 (4/7)	67 (4/7)	0 (0/7)	0 (0/7)	
CST/AZM		50 (4/8)	50 (4/8)	0 (0/8)	0 (0/8)	
CST/TGC		0 (0/9)	NA	NA	NA	
**Hong DJ, 2016 [34]**						
CST/MEM	Fixed-ratio E-test method	85 (35/41)	5 (2/41)	0 (0/41)	81 (33/41)	Based on reported MICs and FICIs ≤ 0.5 it was possible to confirm synergy at concentrations ≤ breakpoints in only 2 cases (with the CST/MEM combination)
CST/IMP		46 (19/41)	0 (0/41)	0 (0/41)	46 (19/41	
CST/RIF		81 (33/41)	0 (0/41)	0 (0/41)	81 (33/41)	
**Yavaş S, 2016 [40]**						
SUL/MEM	Fixed-ratio E-test method	14 (1/7)	0 (0/7)	0 (0/7)	14 (1/7)	FICI=0.5 but MICs not reported for the specific isolate
**Marie MA, 2015 [43]**						

**Study-combinations**	**Method**	**Synergy % (n/N)**	**Synergy at concentrations ≤ breakpoints % (n/N)**	**Synergy at concentrations > breakpoints % (n/N)**	**Synergy but unclear if at concentration ≤ breakpoints % (n/N)**	**Concentrations at which synergy was evaluated**
SUL/MEM	E-test/agar method	41 (22/54)	0 (0/54)	0 (0/54)	41 (22/54)	Exact FICIs or combination MICs not reported. Agar contained SUL or TZB at ½ x MIC.
MEM/TZB		35 (19/54)	0 (0/54)	0 (0/54)	35 (19/54)	
**Phee LM, 2015 [19]**						
CST/FA	Disk/agar method	100 (3/3)	0 (0/3)	100 (3/3)	0 (0/3)	Agar containing CST at 0.5 xMIC i.e. 256 (FA zone diameter 21.5mm), 2 (FA zone diameter 16mm) and 256 mg/L (FA zone diameter 21.5mm).
**Galani I, 2014 [46]**						
CST/DAP	E-test/agar method	0 (0/4)	NA	NA	NA	Daptomycin MIC determined by E-test in agar containing CST at 0.25 and 0.5 x MIC.
**Nastro M, 2014 [48]**						
CST/RIF	E-test/agar method	100 (4/4)	0 (0/4)	100 (4/4)	0 (0/4)	CST MIC determined by E-test in agar containing RIF at 4 mg/L.
**Wang Y, 2014 [52]**						
IMP/RIF	double-disk synergy	100 (1/1)	0 (0/1)	100 (1/1)	0 (0/1)	The sizes of the inhibition zones increased to 13.6 mm when 4 ml/L imipenem (6.2 mm) was combined with 4 mg/L of rifampicin (9.0 mm)
**Cetin ES, 2013 [53]**						
RIF/SAM	Perpendicular E-test method	0 (0/7)	NA	NA	NA	No synergy
RIF/CFS		0 (0/7)	NA	NA	NA	

**Study-combinations**	**Method**	**Synergy % (n/N)**	**Synergy at concentrations ≤ breakpoints % (n/N)**	**Synergy at concentrations > breakpoints % (n/N)**	**Synergy but unclear if at concentration ≤ breakpoints % (n/N)**	**Concentrations at which synergy was evaluated**
**Tan TY, 2011 [64]**						
PMB/TGC	Perpendicular E-test method	0 (0/4)	NA	NA	NA	No synergy
PMB/RIF		33 (1/3)	0 (0/3)	0 (0/3)	33 (1/3)	Clinical relevance with the perpendicular E-test method difficult to judge (not possible to estimate exact combination MICs)
TGC/RIF		33 (1/3)	0 (0/3)	0 (0/3)	33 (1/3)	
**Kiratisin P, 2010 [65]**						
CFS/DOR	Perpendicular E-test method	21 (4/19)	0 (0/19)	0 (0/19)	21 (4/19)	Clinical relevance with the perpendicular E-test method difficult to judge (not possible to estimate exact combination MICs)
DOR/DOX		0 (0/21)	NA	NA	NA	
DOR/RIF		0 (0/17)	NA	NA	NA	
DOR/NET		0 (0/21)	NA	NA	NA	
DOR/MXF		0 (0/21)	NA	NA	NA	
CFS/IMP		47 (9/19)	0 (0/19)	0 (0/19)	47 (9/19)	
IMP/DOX		0 (0/21)	NA	NA	NA	
IMP/RIF		0 (0/17)	NA	NA	NA	
IMP/NET		0 (0/21)	NA	NA	NA	
IMP/MXF		5 (1/21)	0 (0/21)	0 (0/21)	5 (1/21)	
CFS/MEM		53 (10/19)	0 (0/19)	0 (0/19)	53 (10/19)	
MEM/DOX		0 (0/21)	NA	NA	NA	
MEM/RIF		0 (0/17)	NA	NA	NA	
MEM/NET		0 (0/21)	NA	NA	NA	
MEM/MXF		0 (0/21)	NA	NA	NA	

**Study-combinations**	**Method**	**Synergy % (n/N)**	**Synergy at concentrations ≤ breakpoints % (n/N)**	**Synergy at concentrations > breakpoints % (n/N)**	**Synergy but unclear if at concentration ≤ breakpoints % (n/N)**	**Concentrations at which synergy was evaluated**
**Lee NY, 2007 [76]**						
SUL/IMP	Agar dilution	100 (4/4)	100 (4/4)	0 (0/4)	0 (0/4)	Combined MIC: 8/0.5, 8/2, 8/2, 8/2 mg/L
SUL/MEM		100 (4/4)	100 (4/4)	0 (0/4)	0 (0/4)	Combined MIC: 8/1, 8/4, 8/4, 8/4 mg/L

5 Explanations for articles excluded as irrelevant

Following a reviewer's suggestion, reasons for retrieval of irrelevant articles by the search strategy used are discussed here.

Specifically, the search terms (synerg* [ti] OR combin* [ti] OR "Drug Combinations"[Mesh] OR "Drug Synergism"[Mesh] OR "Drug Therapy, Combination"[Mesh]), resulted in retrieval of the following types of non-relevant articles:

1) Irrelevant articles containing the term "synerg*" in the title, e.g.
   a. Articles evaluating synergistic interactions between different bacterial species (e.g. PMID: 25467269)
   b. "Synergistic effect of thermophilic temperature and biosurfactant produced by Acinetobacter calcoaceticus BU03 on the biodegradation of phenanthrene in bioslurry system." (PMID: 21530078)
   c. "Distinct effector-binding sites enable synergistic transcriptional activation by BenM, a LysR-type regulator." (PMID: 17291527)
2) Irrelevant articles containing the term "combin*" in the title, e.g.
   a. "Combined microbial degradation of crude oil under alkaline conditions by Acinetobacter baumannii and Talaromyces sp." (PMID: 33485133)
   b. "Combined Effects Of Low Incubation Temperature, Minimal Growth Medium, And Low Hydrodynamics Optimize Acinetobacter baumannii Biofilm Formation" (PMID: 31814741)
3) Articles evaluating beta-lactam/ beta-lactamase combinations (e.g. PMID: 27312582)
4) Articles evaluating the combination trimethoprim/sulfamethoxazole (e.g. PMID: 31427295)
5) Articles evaluating the effect of combination therapy on metabolomics (e.g. PMID: 33387481)
6) Articles evaluating the effect of combination therapy on persistence (e.g. PMID: 31818819)

6 List of potentially relevant non-English articles excluded

Chinese:

1) Mao HB, He M, He SN. [Significance of Lipopolysaccharide Lipid A Gene Mutation of Extensively Drug-resistant Acinetobacter baumanii on Polymyxin Resistance and Its Influence on Treatment]. **Sichuan Da Xue Xue Bao Yi Xue Ban**. 2021 Jan;52(1):124-128. doi: 10.12182/20210160208.
2) Lu Y, Zhang Y, Zhou H, Yu F, Sun S, Rui Y. [Combined drug sensitivity test of 50 strains of extensively drug-resistant Acinetobacter baumannii]. **Nan Fang Yi Ke Da Xue Xue Bao. 2014** Nov;34(11):1697-701.
3) Zhao C, Xie W, Zhang W, Ye Z, Wu H. [Mechanism of drug resistance of carbapenems-resistant Acinetobacter baumannii and the application of a combination of drugs in vitro]. **Zhonghua Shao Shang Za Zhi. 2014** Apr;30(2):166-70.
4) Ai Y, Dou SS, Lu SJ. Study on in vitro drug sensitivity of minocycline combined with 11 antibiotics against Multidrug resistant Acinetobacter baumannii. **Chinese Journal of New Drugs. 2018** - Volume 27, Issue 6, pp. 702-707
5) Ke Q, Lü Y, Wang F. In vitro activity of meropenem in combination with sulbactam or cefoperazone-sulbactam against multidrug resistant acinetobacter baumannii. **Chinese Journal of Infection and Chemotherapy**. **2015** - Volume 15, Issue 6, pp. 548-551
6) Bai Y, Sun Y, Wang J, Liu X, Wen K, Niu H, Cao J, Tang MJ, Wang R. In vitro antibacterial activity of colistin in combination with other antibacterials against the 73 strains of multidrug-resistant acinetobacter baumannii. **Chinese Pharmaceutical Journal. 2015** - Volume 50, Issue 5, pp. 427-430
7) Wang TS, Su JR. Combined antimicrobial susceptibility test against pan-drug-resistant Acinetobacter baumannii with E-test and microdilution checkerboard assay. **Chinese Journal of Microbiology and Immunology (China), 2013** - Volume 33, Issue 2, pp. 144-147
8) Wang TS, Su JR. Different activities of antimicrobial combinations against multidrug resistant Acinetobacter baumannii in vitro. **Chinese Journal of Microbiology and Immunology (China). 2011** - Volume 31, Issue 10, pp. 898-902
9) Xie F, Ding YJ, Zhou SD. Effect of two antibiotics combination on multi-drug resistant acinetobacter calcoaceticus-baumannii. **Chinese Pharmaceutical Journal. 2010** - Volume 45, Issue 6, pp. 476-478

Turkish:

1) Akyüz S, Parlak M, Güdücüoğlu H. [In-vitro Activity of Ceftolozane-Tazobactam in Combination with Various Antibiotics Against Multidrug-resistant Acinetobacter baumannii Isolated from Intensive Care Patients]. **Mikrobiyol Bul. 2020** Jan;54(1):154-162. doi: 10.5578/mb.68981.
2) Zarakolu P, Ayaz ÇM, Metan G. [Various antibiotic combinations against carbapenem resistant Acinetobacter baumannii infections and in vitro synergy test results (2002-2016)]. **Mikrobiyol Bul. 2018** Apr;52(2):190-197. doi: 10.5578/mb.61903.

3) Çetinkol Y, Telli M, Altunçekiç Yıldırım A, Çalgın MK. [Evaluation of the efficacy of colistin/sulbactam combination on carbapenem-resistant Acinetobacter baumannii strains]. **Mikrobiyol Bul. 2016** Jul;50(3):460-5. doi: 10.5578/mb.26289.
4) Turk Dagi H, Kus H, Arslan U, Tuncer I. [In vitro synergistic activity of sulbactam in combination with imipenem, meropenem and cefoperazone against carbapenem-resistant Acinetobacter baumannii isolates]. **Mikrobiyol Bul. 2014** Apr;48(2):311-5. doi: 10.5578/mb.7104.
5) Cıkman A, Ceylan MR, Parlak M, Karahocagil MK, Berktaş M. [Evaluation of Colistin-Ampicillin/Sulbactam Combination Efficacy in Imipenem-Resistant Acinetobacter baumannii Strains]. **Mikrobiyol Bul. 2013** Jan;47(1):147-51. doi: 10.5578/mb.4523.
6) Ozseven AG, Sesli Çetin E, Ozseven L. [Do different interpretative methods used for evaluation of checkerboard synergy test affect the results?]. **Mikrobiyol Bul. 2012** Jul;46(3):410-20.

<u>French</u>:

1) Elkhaïli H, Pompei D, Linger L, Kamili N, Monteil H, Jehl F. [Kinetics of bactericidal activity of cefepime and cefpirome alone or combined with gentamicin, amikacin or ciprofloxacin against Acinetobacter baumannii, Stenotrophomonas maltophilia and Enterobacter cloacae hyperproductive in cephalosporinase]. **Pathol Biol (Paris). 1996** May;44(5):367-73.
2) Hercouet H, Bousser J, Donnio PY, Avril JL. [In vitro effect of antibiotics against hospital strains of Acinetobacter baumanii]. **Pathol Biol (Paris). 1989** Jun;37(5 Pt 2):612-6.
3) Le Noc P, Croize J, Bryskier A, Le Noc D. [In vitro antibacterial activity of cefpirome in combination with 4 aminoglycosides and 2 fluoroquinolones]. **Pathol Biol (Paris). 1988** Jun;36(5 Pt 2):762-7.
4) Berardi-Grassias L, Bigel ML, Macheboeuf F, Boisivon A, Chauvière P, Guiomar C. [In vitro study of the pefloxacine-ceftriaxone combination against 18 strains of Acinetobacter]. **Pathol Biol (Paris). 1986** May;34(5):436-9.
5) Kitzis MD, Goldstein FW, Labia R, Acar JF. [Activity of sulbactam and clavulanic acid, alone and combined, on Acinetobacter calcoaceticus]. **Ann Microbiol (Paris). 1983** Mar-Apr 1983;134A(2):163-8.

<u>Korean</u>:

1) Sung H, Choi SJ, Yoo S, Kim MN. [In vitro antimicrobial synergy against imipenem-resistant Acinetobacter baumannii]. **Korean J Lab Med. 2007** Apr;27(2):111-7. doi: 10.3343/kjlm.2007.27.2.111.
2) Hang JJ, Kim MN, Lee K, Hong SB, Lim CM, Koh Y. The comparative efficacy of colistin monotherapy and combination therapy based on in vitro antimicrobial synergy in ventilator-associated pneumonia caused by multi-drug resistant Acinetobacter baumannii. **Tuberculosis and Respiratory Diseases. 2009** - Volume 67, Issue 3, pp. 212-220

__Japanese__:

1) Deguchi K. [Investigation in vitro synergism on ampicillin with cloxacillin and dibekacin against glucose non-fermenting gram-negative bacilli and Serratia (author's transl)]. **Jpn J Antibiot. 1978** Oct;31(10):610-3.

7 References

1. Cebrero-Cangueiro, T.; Nordmann, P.; Carretero-Ledesma, M.; Pachón, J.; Pachón-Ibáñez, M.E. Efficacy of dual carbapenem treatment in a murine sepsis model of infection due to carbapenemase-producing Acinetobacter baumannii. *J. Antimicrob. Chemother.* **2021**, *76*, 680-683, doi:10.1093/jac/dkaa487.
2. Nordmann, P.; Perler, J.; Kieffer, N.; Poirel, L. In-vitro evaluation of a dual carbapenem combination against carbapenemase-producing Acinetobacter baumannii. *J. Infect.* **2020**, *80*, 121-142, doi:10.1016/j.jinf.2019.10.003.
3. Cheng, J.; Yan, J.; Reyna, Z.; Slarve, M.; Lu, P.; Spellberg, B.; Luna, B. Synergistic Rifabutin and Colistin Reduce Emergence of Resistance When Treating Acinetobacter baumannii. *Antimicrob. Agents Chemother.* **2021**, *65*, doi:10.1128/aac.02204-20.
4. Nwabor, O.F.; Terbtothakun, P.; Voravuthikunchai, S.P.; Chusri, S. Evaluation of the Synergistic Antibacterial Effects of Fosfomycin in Combination with Selected Antibiotics against Carbapenem–Resistant Acinetobacter baumannii. *Pharmaceuticals* **2021**, *14*, 185.
5. Armengol, E.; Asunción, T.; Viñas, M.; Sierra, J.M. When Combined with Colistin, an Otherwise Ineffective Rifampicin-Linezolid Combination Becomes Active in Escherichia coli, Pseudomonas aeruginosa, and Acinetobacter baumannii. *Microorganisms* **2020**, *8*, doi:10.3390/microorganisms8010086.
6. Li, J.; Fu, Y.; Zhang, J.; Zhao, Y.; Fan, X.; Yu, L.; Wang, Y.; Zhang, X.; Li, C. The efficacy of colistin monotherapy versus combination therapy with other antimicrobials against carbapenem-resistant Acinetobacter baumannii ST2 isolates. *J. Chemother.* **2020**, *32*, 359-367, doi:10.1080/1120009x.2020.1764282.
7. Limsrivanichakorn, S.; Ngamskulrungroj, P.; Leelaporn, A. Activity of Antimicrobial Combinations Against Extensively Drug-Resistant Acinetobacter baumannii as Determined by Checkerboard Method and E-test. *Siriraj Medical Journal* **2020**, *72*, 214-218, doi:https://doi.org/10.33192/Smj.2020.29.
8. Mohd Sazlly Lim, S.; Naicker, S.; Ayfan, A.K.; Zowawi, H.; Roberts, J.A.; Sime, F.B. Non-polymyxin-based combinations as potential alternatives in treatment against carbapenem-resistant Acinetobacter baumannii infections. *Int. J. Antimicrob. Agents* **2020**, *56*, 106115, doi:10.1016/j.ijantimicag.2020.106115.
9. Mohd Sazlly Lim, S.; Heffernan, A.J.; Roberts, J.A.; Sime, F.B. Semi-mechanistic PK/PD modelling of fosfomycin and sulbactam combination against carbapenem-resistant Acinetobacter baumannii. *Antimicrob. Agents Chemother.* **2021**, doi:10.1128/aac.02472-20.
10. Mohd Sazlly Lim, S.; Heffernan, A.J.; Roberts, J.A.; Sime, F.B. Pharmacodynamic Analysis of Meropenem and Fosfomycin Combination Against Carbapenem-Resistant Acinetobacter baumannii in Patients with Normal Renal Clearance: Can It Be a Treatment Option? *Microb Drug Resist* **2021**, *27*, 546-552, doi:10.1089/mdr.2020.0197.
11. Rodriguez, C.H.; Brune, A.; Nastro, M.; Vay, C.; Famiglietti, A. In vitro synergistic activity of the sulbactam/avibactam combination against extensively drug-resistant

Acinetobacter baumannii. *J. Med. Microbiol.* **2020**, *69*, 928-931, doi:https://doi.org/10.1099/jmm.0.001211.
12. Gaudereto, J.J.; Perdigão Neto, L.V.; Leite, G.C.; Ruedas Martins, R.; Boas do Prado, G.V.; Rossi, F.; Guimarães, T.; Levin, A.S.; Costa, S.F. Synergistic Effect of Ceftazidime-Avibactam with Meropenem against Panresistant, Carbapenemase-Harboring Acinetobacter baumannii and Serratia marcescens Investigated Using Time-Kill and Disk Approximation Assays. *Antimicrob. Agents Chemother.* **2019**, *63*, doi:10.1128/aac.02367-18.
13. Ghaith, D.; Hassan, R.; Dawoud, M.E.E.; Eweis, M.; Metwally, R.; Zafer, M. Effect of rifampicin-colistin combination against XDR Acinetobacter baumannii harbouring bla(OXA 23)-like gene and showed reduced susceptibility to colistin at Cairo University Hospital, Cairo, Egypt. *Infect Dis (Lond)* **2019**, *51*, 308-311, doi:10.1080/23744235.2018.1558369.
14. Mataracı Kara, E.; Yılmaz, M.; Özbek Çelik, B. In vitro activities of ceftazidime/avibactam alone or in combination with antibiotics against multidrug-resistant Acinetobacter baumannii isolates. *J Glob Antimicrob Resist* **2019**, *17*, 137-141, doi:10.1016/j.jgar.2018.12.004.
15. Menegucci, T.C.; Fedrigo, N.H.; Lodi, F.G.; Albiero, J.; Nishiyama, S.A.B.; Mazucheli, J.; Carrara-Marroni, F.E.; Voelkner, N.M.F.; Gong, H.; Sy, S.K.B.; et al. Pharmacodynamic Effects of Sulbactam/Meropenem/Polymyxin-B Combination Against Extremely Drug Resistant Acinetobacter baumannii Using Checkerboard Information. *Microb Drug Resist* **2019**, *25*, 1266-1274, doi:10.1089/mdr.2018.0283.
16. Menegucci, T.C.; Albiero, J.; Migliorini, L.B.; Alves, J.L.; Viana, G.F.; Mazucheli, J.; Carrara-Marroni, F.E.; Cardoso, C.L.; Tognim, M.C. Strategies for the treatment of polymyxin B-resistant Acinetobacter baumannii infections. *Int. J. Antimicrob. Agents* **2016**, *47*, 380-385, doi:10.1016/j.ijantimicag.2016.02.007.
17. Oliva, A.; Garzoli, S.; De Angelis, M.; Marzuillo, C.; Vullo, V.; Mastroianni, C.M.; Ragno, R. In-Vitro Evaluation of Different Antimicrobial Combinations with and without Colistin Against Carbapenem-Resistant Acinetobacter Baumannii. *Molecules* **2019**, *24*, doi:10.3390/molecules24050886.
18. Ozger, H.S.; Cuhadar, T.; Yildiz, S.S.; Demirbas Gulmez, Z.; Dizbay, M.; Guzel Tunccan, O.; Kalkanci, A.; Simsek, H.; Unaldi, O. In vitro activity of eravacycline in combination with colistin against carbapenem-resistant A. baumannii isolates. *J. Antibiot. (Tokyo)* **2019**, *72*, 600-604, doi:10.1038/s41429-019-0188-6.
19. Phee, L.M.; Betts, J.W.; Bharathan, B.; Wareham, D.W. Colistin and Fusidic Acid, a Novel Potent Synergistic Combination for Treatment of Multidrug-Resistant Acinetobacter baumannii Infections. *Antimicrob. Agents Chemother.* **2015**, *59*, 4544-4550, doi:10.1128/aac.00753-15.
20. Phee, L.M.; Kloprogge, F.; Morris, R.; Barrett, J.; Wareham, D.W.; Standing, J.F. Pharmacokinetic-pharmacodynamic modelling to investigate in vitro synergy between colistin and fusidic acid against MDR Acinetobacter baumannii. *J. Antimicrob. Chemother.* **2019**, *74*, 961-969, doi:10.1093/jac/dky524.
21. Poulakou, G.; Renieris, G.; Sabrakos, L.; Zarkotou, O.; Themeli-Digalaki, K.; Perivolioti, E.; Kraniotaki, E.; Giamarellos-Bourboulis, E.J.; Zavras, N. Daptomycin as adjunctive treatment for experimental infection by Acinetobacter baumannii with resistance to colistin. *Int. J. Antimicrob. Agents* **2018**, doi:10.1016/j.ijantimicag.2018.10.024.

22. Shinohara, D.R.; Menegucci, T.C.; Fedrigo, N.H.; Migliorini, L.B.; Carrara-Marroni, F.E.; Maria Dos Anjos, M.; Cardoso, C.L.; Nishiyama, S.A.B.; Tognim, M.C.B. Synergistic activity of polymyxin B combined with vancomycin against carbapenem-resistant and polymyxin-resistant Acinetobacter baumannii: first in vitro study. *J. Med. Microbiol.* **2019**, *68*, 309-315, doi:10.1099/jmm.0.000920.
23. Wang, J.; Ning, Y.; Li, S.; Wang, Y.; Liang, J.; Jin, C.; Yan, H.; Huang, Y. Multidrug-resistant Acinetobacter baumannii strains with NDM-1: Molecular characterization and in vitro efficacy of meropenem-based combinations. *Exp. Ther. Med.* **2019**, *18*, 2924-2932, doi:10.3892/etm.2019.7927.
24. Chen, F.; Wang, L.; Wang, M.; Xie, Y.; Xia, X.; Li, X.; Liu, Y.; Cao, W.; Zhang, T.; Li, P.; et al. Genetic characterization and in vitro activity of antimicrobial combinations of multidrug-resistant Acinetobacter baumannii from a general hospital in China. *Oncol. Lett.* **2018**, *15*, 2305-2315, doi:10.3892/ol.2017.7600.
25. Singkham-In, U.; Chatsuwan, T. In vitro activities of carbapenems in combination with amikacin, colistin, or fosfomycin against carbapenem-resistant Acinetobacter baumannii clinical isolates. *Diagn. Microbiol. Infect. Dis.* **2018**, *91*, 169-174, doi:10.1016/j.diagmicrobio.2018.01.008.
26. Zhu, W.; Wang, Y.; Cao, W.; Cao, S.; Zhang, J. In vitro evaluation of antimicrobial combinations against imipenem-resistant Acinetobacter baumannii of different MICs. *J Infect Public Health* **2018**, *11*, 856-860, doi:10.1016/j.jiph.2018.07.006.
27. Lenhard, J.R.; Thamlikitkul, V.; Silveira, F.P.; Garonzik, S.M.; Tao, X.; Forrest, A.; Soo Shin, B.; Kaye, K.S.; Bulitta, J.B.; Nation, R.L.; et al. Polymyxin-resistant, carbapenem-resistant Acinetobacter baumannii is eradicated by a triple combination of agents that lack individual activity. *J. Antimicrob. Chemother.* **2017**, *72*, 1415-1420, doi:10.1093/jac/dkx002.
28. Lenhard, J.R.; Smith, N.M.; Bulman, Z.P.; Tao, X.; Thamlikitkul, V.; Shin, B.S.; Nation, R.L.; Li, J.; Bulitta, J.B.; Tsuji, B.T. High-Dose Ampicillin-Sulbactam Combinations Combat Polymyxin-Resistant Acinetobacter baumannii in a Hollow-Fiber Infection Model. *Antimicrob. Agents Chemother.* **2017**, *61*, e01268-01216, doi:10.1128/aac.01268-16.
29. Madadi-Goli, N.; Moniri, R.; Bagheri-Josheghani, S.; Dasteh-Goli, N. Sensitivity of levofloxacin in combination with ampicillin-sulbactam and tigecycline against multidrug-resistant Acinetobacter baumannii. *Iran J Microbiol* **2017**, *9*, 19-25.
30. Wei, W.; Yang, H.; Hu, L.; Ye, Y.; Li, J. Activity of levofloxacin in combination with colistin against Acinetobacter baumannii: In vitro and in a Galleria mellonella model. *J. Microbiol. Immunol. Infect.* **2017**, *50*, 821-830, doi:10.1016/j.jmii.2015.10.010.
31. Wei, W.J.; Yang, H.F. Synergy against extensively drug-resistant Acinetobacter baumannii in vitro by two old antibiotics: colistin and chloramphenicol. *Int. J. Antimicrob. Agents* **2017**, *49*, 321-326, doi:10.1016/j.ijantimicag.2016.11.031.
32. Bae, S.; Kim, M.-C.; Park, S.-J.; Kim, H.S.; Sung, H.; Kim, M.-N.; Kim, S.-H.; Lee, S.-O.; Choi, S.-H.; Woo, J.H.; et al. In Vitro Synergistic Activity of Antimicrobial Agents in Combination against Clinical Isolates of Colistin-Resistant Acinetobacter baumannii. *Antimicrob. Agents Chemother.* **2016**, *60*, 6774-6779, doi:10.1128/AAC.00839-16.
33. Bowler, S.L.; Spychala, C.N.; McElheny, C.L.; Mettus, R.T.; Doi, Y. In Vitro Activity of Fusidic Acid-Containing Combinations against Carbapenem-Resistant Acinetobacter

baumannii Clinical Strains. *Antimicrob. Agents Chemother.* **2016**, *60*, 5101, doi:10.1128/aac.01124-16.
34. Hong, D.J.; Kim, J.O.; Lee, H.; Yoon, E.J.; Jeong, S.H.; Yong, D.; Lee, K. In vitro antimicrobial synergy of colistin with rifampicin and carbapenems against colistin-resistant Acinetobacter baumannii clinical isolates. *Diagn. Microbiol. Infect. Dis.* **2016**, *86*, 184-189, doi:10.1016/j.diagmicrobio.2016.07.017.
35. Laishram, S.; Anandan, S.; Devi, B.Y.; Elakkiya, M.; Priyanka, B.; Bhuvaneshwari, T.; Peter, J.V.; Subramani, K.; Balaji, V. Determination of synergy between sulbactam, meropenem and colistin in carbapenem-resistant Klebsiella pneumoniae and Acinetobacter baumannii isolates and correlation with the molecular mechanism of resistance. *J. Chemother.* **2016**, *28*, 297-303, doi:10.1080/1120009x.2016.1143261.
36. Leite, G.C.; Oliveira, M.S.; Perdigao-Neto, L.V.; Rocha, C.K.; Guimaraes, T.; Rizek, C.; Levin, A.S.; Costa, S.F. Antimicrobial Combinations against Pan-Resistant Acinetobacter baumannii Isolates with Different Resistance Mechanisms. *PLoS One* **2016**, *11*, e0151270, doi:10.1371/journal.pone.0151270.
37. Park, G.C.; Choi, J.A.; Jang, S.J.; Jeong, S.H.; Kim, C.M.; Choi, I.S.; Kang, S.H.; Park, G.; Moon, D.S. In Vitro Interactions of Antibiotic Combinations of Colistin, Tigecycline, and Doripenem Against Extensively Drug-Resistant and Multidrug-Resistant Acinetobacter baumannii. *Ann. Lab. Med.* **2016**, *36*, 124-130, doi:10.3343/alm.2016.36.2.124.
38. Yang, H.; Lv, N.; Hu, L.; Liu, Y.; Cheng, J.; Ye, Y.; Li, J. In vivo activity of vancomycin combined with colistin against multidrug-resistant strains of Acinetobacter baumannii in a Galleria mellonella model. *Infect Dis (Lond)* **2016**, *48*, 189-194, doi:10.3109/23744235.2015.1103894.
39. Yang, Y.S.; Lee, Y.; Tseng, K.C.; Huang, W.C.; Chuang, M.F.; Kuo, S.C.; Lauderdale, T.L.; Chen, T.L. In Vivo and In Vitro Efficacy of Minocycline-Based Combination Therapy for Minocycline-Resistant Acinetobacter baumannii. *Antimicrob. Agents Chemother.* **2016**, *60*, 4047-4054, doi:10.1128/aac.02994-15.
40. Yavaş, S.; Yetkin, M.A.; Kayaaslan, B.; Baştuğ, A.; Aslaner, H.; But, A.; Kanyilmaz, D.; Sari, B.; Akinci, E.; Bodur, H. Investigating the in vitro synergistic activities of several antibiotic combinationsagainst carbapenem-resistant Acinetobacter baumannii isolates. *Turk J Med Sci* **2016**, *46*, 892-896, doi:10.3906/sag-1408-14.
41. Córdoba, J.; Coronado-Álvarez, N.M.; Parra, D.; Parra-Ruiz, J. In Vitro Activities of Novel Antimicrobial Combinations against Extensively Drug-Resistant Acinetobacter baumannii. *Antimicrob. Agents Chemother.* **2015**, *59*, 7316-7319, doi:10.1128/aac.00493-15.
42. García-Salguero, C.; Rodríguez-Avial, I.; Picazo, J.J.; Culebras, E. Can Plazomicin Alone or in Combination Be a Therapeutic Option against Carbapenem-Resistant Acinetobacter baumannii? *Antimicrob. Agents Chemother.* **2015**, *59*, 5959-5966, doi:10.1128/AAC.00873-15.
43. Marie, M.A.; Krishnappa, L.G.; Alzahrani, A.J.; Mubaraki, M.A.; Alyousef, A.A. A prospective evaluation of synergistic effect of sulbactam and tazobactam combination with meropenem or colistin against multidrug resistant Acinetobacter baumannii. *Bosn. J. Basic Med. Sci.* **2015**, *15*, 24-29, doi:10.17305/bjbms.2015.526.
44. Rodríguez, C.H.; Nastro, M.; Vay, C.; Famiglietti, A. In vitro activity of minocycline alone or in combination in multidrug-resistant Acinetobacter baumannii isolates. *J. Med. Microbiol.* **2015**, *64*, 1196-1200, doi:10.1099/jmm.0.000147.

45. Vourli, S.; Frantzeskaki, F.; Meletiadis, J.; Stournara, L.; Armaganidis, A.; Zerva, L.; Dimopoulos, G. Synergistic interactions between colistin and meropenem against extensively drug-resistant and pandrug-resistant Acinetobacter baumannii isolated from ICU patients. *Int. J. Antimicrob. Agents* **2015**, *45*, 670-671, doi:https://doi.org/10.1016/j.ijantimicag.2015.02.005.
46. Galani, I.; Orlandou, K.; Moraitou, H.; Petrikkos, G.; Souli, M. Colistin/daptomycin: an unconventional antimicrobial combination synergistic in vitro against multidrug-resistant Acinetobacter baumannii. *Int. J. Antimicrob. Agents* **2014**, *43*, 370-374, doi:10.1016/j.ijantimicag.2013.12.010.
47. Majewski, P.; Wieczorek, P.; Ojdana, D.; Sacha, P.T.; Wieczorek, A.; Tryniszewska, E.A. In vitro activity of rifampicin alone and in combination with imipenem against multidrug-resistant Acinetobacter baumannii harboring the blaOXA-72 resistance gene. *Scand. J. Infect. Dis.* **2014**, *46*, 260-264, doi:10.3109/00365548.2013.865141.
48. Nastro, M.; Rodríguez, C.H.; Monge, R.; Zintgraff, J.; Neira, L.; Rebollo, M.; Vay, C.; Famiglietti, A. Activity of the colistin-rifampicin combination against colistin-resistant, carbapenemase-producing Gram-negative bacteria. *J. Chemother.* **2014**, *26*, 211-216, doi:10.1179/1973947813y.0000000136.
49. Oleksiuk, L.M.; Nguyen, M.H.; Press, E.G.; Updike, C.L.; O'Hara, J.A.; Doi, Y.; Clancy, C.J.; Shields, R.K. In vitro responses of Acinetobacter baumannii to two- and three-drug combinations following exposure to colistin and doripenem. *Antimicrob. Agents Chemother.* **2014**, *58*, 1195-1199, doi:10.1128/aac.01779-13.
50. Percin, D.; Akyol, S.; Kalin, G. In vitro synergism of combinations of colistin with selected antibiotics against colistin-resistant Acinetobacter baumannii. *GMS Hyg Infect Control* **2014**, *9*, Doc14, doi:10.3205/dgkh000234.
51. Sun, Y.; Wang, L.; Li, J.; Zhao, C.; Zhao, J.; Liu, M.; Wang, S.; Lu, C.; Shang, G.; Jia, Y.; et al. Synergistic efficacy of meropenem and rifampicin in a murine model of sepsis caused by multidrug-resistant Acinetobacter baumannii. *Eur. J. Pharmacol.* **2014**, *729*, 116-122, doi:10.1016/j.ejphar.2014.02.015.
52. Wang, Y.; Bao, W.; Guo, N.; Chen, H.; Cheng, W.; Jin, K.; Shen, F.; Xu, J.; Zhang, Q.; Wang, C.; et al. Antimicrobial activity of the imipenem/rifampicin combination against clinical isolates of Acinetobacter baumannii grown in planktonic and biofilm cultures. *World J. Microbiol. Biotechnol.* **2014**, *30*, 3015-3025, doi:10.1007/s11274-014-1728-7.
53. Cetin, E.S.; Tekeli, A.; Ozseven, A.G.; Us, E.; Aridogan, B.C. Determination of in vitro activities of polymyxin B and rifampin in combination with ampicillin/sulbactam or cefoperazone/sulbactam against multidrug-resistant Acinetobacter baumannii by the E-test and checkerboard methods. *Jpn. J. Infect. Dis.* **2013**, *66*, 463-468, doi:10.7883/yoken.66.463.
54. Housman, S.T.; Hagihara, M.; Nicolau, D.P.; Kuti, J.L. In vitro pharmacodynamics of human-simulated exposures of ampicillin/sulbactam, doripenem and tigecycline alone and in combination against multidrug-resistant Acinetobacter baumannii. *J. Antimicrob. Chemother.* **2013**, *68*, 2296-2304, doi:10.1093/jac/dkt197.
55. Lee, H.J.; Bergen, P.J.; Bulitta, J.B.; Tsuji, B.; Forrest, A.; Nation, R.L.; Li, J. Synergistic activity of colistin and rifampin combination against multidrug-resistant Acinetobacter baumannii in an in vitro pharmacokinetic/pharmacodynamic model. *Antimicrob. Agents Chemother.* **2013**, *57*, 3738-3745, doi:10.1128/aac.00703-13.

56. O'Hara, J.A.; Ambe, L.A.; Casella, L.G.; Townsend, B.M.; Pelletier, M.R.; Ernst, R.K.; Shanks, R.M.Q.; Doi, Y. Activities of vancomycin-containing regimens against colistin-resistant Acinetobacter baumannii clinical strains. *Antimicrob. Agents Chemother.* **2013**, *57*, 2103-2108, doi:10.1128/AAC.02501-12.
57. Principe, L.; Capone, A.; Mazzarelli, A.; D'Arezzo, S.; Bordi, E.; Di Caro, A.; Petrosillo, N. In vitro activity of doripenem in combination with various antimicrobials against multidrug-resistant Acinetobacter baumannii: possible options for the treatment of complicated infection. *Microb Drug Resist* **2013**, *19*, 407-414, doi:10.1089/mdr.2012.0250.
58. Queenan, A.M.; Davies, T.A.; He, W.; Lynch, A.S. Assessment of the combination of doripenem plus a fluoroquinolone against non-susceptible Acinetobacter baumannii isolates from nosocomial pneumonia patients. *J. Chemother.* **2013**, *25*, 141-147, doi:10.1179/1973947813y.0000000074.
59. Deveci, A.; Coban, A.Y.; Acicbe, O.; Tanyel, E.; Yaman, G.; Durupinar, B. In vitro effects of sulbactam combinations with different antibiotic groups against clinical Acinetobacter baumannii isolates. *J. Chemother.* **2012**, *24*, 247-252, doi:10.1179/1973947812y.0000000029.
60. Peck, K.R.; Kim, M.J.; Choi, J.Y.; Kim, H.S.; Kang, C.I.; Cho, Y.K.; Park, D.W.; Lee, H.J.; Lee, M.S.; Ko, K.S. In vitro time-kill studies of antimicrobial agents against blood isolates of imipenem-resistant Acinetobacter baumannii, including colistin- or tigecycline-resistant isolates. *J. Med. Microbiol.* **2012**, *61*, 353-360, doi:10.1099/jmm.0.036939-0.
61. Vidaillac, C.; Benichou, L.; Duval, R.E. In vitro synergy of colistin combinations against colistin-resistant Acinetobacter baumannii, Pseudomonas aeruginosa, and Klebsiella pneumoniae isolates. *Antimicrob. Agents Chemother.* **2012**, *56*, 4856-4861, doi:10.1128/aac.05996-11.
62. Santimaleeworagun, W.; Wongpoowarak, P.; Chayakul, P.; Pattharachayakul, S.; Tansakul, P.; Garey, K.W. In vitro activity of colistin or sulbactam in combination with fosfomycin or imipenem against clinical isolates of carbapenem-resistant Acinetobacter baumannii producing OXA-23 carbapenemases. *Southeast Asian J. Trop. Med. Public Health* **2011**, *42*, 890-900.
63. Pachón-Ibáñez, M.E.; Docobo-Pérez, F.; Jiménez-Mejias, M.E.; Ibáñez-Martínez, J.; García-Curiel, A.; Pichardo, C.; Pachón, J. Efficacy of rifampin, in monotherapy and in combinations, in an experimental murine pneumonia model caused by panresistant Acinetobacter baumannii strains. *Eur. J. Clin. Microbiol. Infect. Dis.* **2011**, *30*, 895-901, doi:10.1007/s10096-011-1173-6.
64. Tan, T.Y.; Lim, T.P.; Lee, W.H.; Sasikala, S.; Hsu, L.Y.; Kwa, A.L. In vitro antibiotic synergy in extensively drug-resistant Acinetobacter baumannii: the effect of testing by time-kill, checkerboard, and Etest methods. *Antimicrob. Agents Chemother.* **2011**, *55*, 436-438, doi:10.1128/aac.00850-10.
65. Kiratisin, P.; Apisarnthanarak, A.; Kaewdaeng, S. Synergistic activities between carbapenems and other antimicrobial agents against Acinetobacter baumannii including multidrug-resistant and extensively drug-resistant isolates. *Int. J. Antimicrob. Agents* **2010**, *36*, 243-246, doi:10.1016/j.ijantimicag.2010.04.011.
66. Pachón-Ibáñez, M.E.; Docobo-Pérez, F.; López-Rojas, R.; Domínguez-Herrera, J.; Jiménez-Mejias, M.E.; García-Curiel, A.; Pichardo, C.; Jiménez, L.; Pachón, J. Efficacy of rifampin and its combinations with imipenem, sulbactam, and colistin in experimental models of

infection caused by imipenem-resistant Acinetobacter baumannii. *Antimicrob. Agents Chemother.* **2010**, *54*, 1165-1172, doi:10.1128/aac.00367-09.
67. Pankuch, G.A.; Seifert, H.; Appelbaum, P.C. Activity of doripenem with and without levofloxacin, amikacin, and colistin against Pseudomonas aeruginosa and Acinetobacter baumannii. *Diagn. Microbiol. Infect. Dis.* **2010**, *67*, 191-197, doi:10.1016/j.diagmicrobio.2010.01.004.
68. Rodriguez, C.H.; De Ambrosio, A.; Bajuk, M.; Spinozzi, M.; Nastro, M.; Bombicino, K.; Radice, M.; Gutkind, G.; Vay, C.; Famiglietti, A. In vitro antimicrobials activity against endemic Acinetobacter baumannii multiresistant clones. *J Infect Dev Ctries* **2010**, *4*, 164-167, doi:10.3855/jidc.604.
69. Urban, C.; Mariano, N.; Rahal, J.J. In vitro double and triple bactericidal activities of doripenem, polymyxin B, and rifampin against multidrug-resistant Acinetobacter baumannii, Pseudomonas aeruginosa, Klebsiella pneumoniae, and Escherichia coli. *Antimicrob. Agents Chemother.* **2010**, *54*, 2732-2734, doi:10.1128/aac.01768-09.
70. Yuan, Z.; Ledesma, K.R.; Singh, R.; Hou, J.; Prince, R.A.; Tam, V.H. Quantitative assessment of combination antimicrobial therapy against multidrug-resistant bacteria in a murine pneumonia model. *J. Infect. Dis.* **2010**, *201*, 889-897, doi:10.1086/651024.
71. Lim, T.P.; Ledesma, K.R.; Chang, K.T.; Hou, J.G.; Kwa, A.L.; Nikolaou, M.; Quinn, J.P.; Prince, R.A.; Tam, V.H. Quantitative assessment of combination antimicrobial therapy against multidrug-resistant Acinetobacter baumannii. *Antimicrob. Agents Chemother.* **2008**, *52*, 2898-2904, doi:10.1128/aac.01309-07.
72. Lim, T.P.; Tan, T.Y.; Lee, W.; Sasikala, S.; Tan, T.T.; Hsu, L.Y.; Kwa, A.L. In vitro activity of various combinations of antimicrobials against carbapenem-resistant Acinetobacter species in Singapore. *J. Antibiot. (Tokyo)* **2009**, *62*, 675-679, doi:10.1038/ja.2009.99.
73. Principe, L.; D'Arezzo, S.; Capone, A.; Petrosillo, N.; Visca, P. In vitro activity of tigecycline in combination with various antimicrobials against multidrug resistant Acinetobacter baumannii. *Ann. Clin. Microbiol. Antimicrob.* **2009**, *8*, 18, doi:10.1186/1476-0711-8-18.
74. Song, J.Y.; Cheong, H.J.; Lee, J.; Sung, A.K.; Kim, W.J. Efficacy of monotherapy and combined antibiotic therapy for carbapenem-resistant Acinetobacter baumannii pneumonia in an immunosuppressed mouse model. *Int. J. Antimicrob. Agents* **2009**, *33*, 33-39, doi:10.1016/j.ijantimicag.2008.07.008.
75. Lee, C.H.; Tang, Y.F.; Su, L.H.; Chien, C.C.; Liu, J.W. Antimicrobial effects of varied combinations of meropenem, sulbactam, and colistin on a multidrug-resistant Acinetobacter baumannii isolate that caused meningitis and bacteremia. *Microb Drug Resist* **2008**, *14*, 233-237, doi:10.1089/mdr.2008.0840.
76. Lee, N.Y.; Wang, C.L.; Chuang, Y.C.; Yu, W.L.; Lee, H.C.; Chang, C.M.; Wang, L.R.; Ko, W.C. Combination carbapenem-sulbactam therapy for critically ill patients with multidrug-resistant Acinetobacter baumannii bacteremia: four case reports and an in vitro combination synergy study. *Pharmacotherapy* **2007**, *27*, 1506-1511, doi:10.1592/phco.27.11.1506.
77. Sader, H.S.; Jones, R.N. Comprehensive in vitro evaluation of cefepime combined with aztreonam or ampicillin/sulbactam against multi-drug resistant Pseudomonas aeruginosa and Acinetobacter spp. *Int. J. Antimicrob. Agents* **2005**, *25*, 380-384, doi:10.1016/j.ijantimicag.2005.01.011.

78. Sader, H.S.; Rhomberg, P.R.; Jones, R.N. In vitro activity of beta-lactam antimicrobial agents in combination with aztreonam tested against metallo-beta-lactamase-producing Pseudomonas aeruginosa and Acinetobacter baumannii. *J. Chemother.* **2005**, *17*, 622-627, doi:10.1179/joc.2005.17.6.622.
79. Choi, J.Y.; Park, Y.S.; Cho, C.H.; Park, Y.S.; Shin, S.Y.; Song, Y.G.; Yong, D.; Lee, K.; Kim, J.M. Synergic in-vitro activity of imipenem and sulbactam against Acinetobacter baumannii. *Clin. Microbiol. Infect.* **2004**, *10*, 1098-1101, doi:10.1111/j.1469-0691.2004.00987.x.
80. Jung, R.; Husain, M.; Choi, M.K.; Fish, D.N. Synergistic activities of moxifloxacin combined with piperacillin-tazobactam or cefepime against Klebsiella pneumoniae, Enterobacter cloacae, and Acinetobacter baumannii clinical isolates. *Antimicrob. Agents Chemother.* **2004**, *48*, 1055-1057, doi:10.1128/aac.48.3.1055-1057.2004.
81. Montero, A.; Ariza, J.; Corbella, X.; Doménech, A.; Cabellos, C.; Ayats, J.; Tubau, F.; Borraz, C.; Gudiol, F. Antibiotic combinations for serious infections caused by carbapenem-resistant Acinetobacter baumannii in a mouse pneumonia model. *J. Antimicrob. Chemother.* **2004**, *54*, 1085-1091, doi:10.1093/jac/dkh485.
82. Yoon, J.; Urban, C.; Terzian, C.; Mariano, N.; Rahal, J.J. In vitro double and triple synergistic activities of Polymyxin B, imipenem, and rifampin against multidrug-resistant Acinetobacter baumannii. *Antimicrob. Agents Chemother.* **2004**, *48*, 753-757, doi:10.1128/aac.48.3.753-757.2004.
83. Fernández-Cuenca, F.; Martínez-Martínez, L.; Pascual, A.; Perea, E.J. In vitro activity of azithromycin in combination with amikacin, ceftazidime, ciprofloxacin or imipenem against clinical isolates of Acinobacter baumannii. *Chemotherapy* **2003**, *49*, 24-26, doi:10.1159/000069774.
84. Roussel-Delvallez, M.; Wallet, F.; Delpierre, F.; Courcol, R.J. In vitro bactericidal effect of a beta-lactam+aminoglycoside combination against multiresistant Pseudomonas aeruginosa and Acinetobacter baumannii. *J. Chemother.* **1996**, *8*, 365-368, doi:10.1179/joc.1996.8.5.365.
85. Mohd Sazlly Lim, S.; Heffernan, A.J.; Zowawi, H.M.; Roberts, J.A.; Sime, F.B. Semi-mechanistic PK/PD modelling of meropenem and sulbactam combination against carbapenem-resistant strains of Acinetobacter baumannii. *Eur. J. Clin. Microbiol. Infect. Dis.* **2021**, *40*, 1943-1952, doi:10.1007/s10096-021-04252-z.
